# Structural aspects of lesional and non-lesional skin microbiota reveal key community changes in leprosy patients from India

**DOI:** 10.1038/s41598-020-80533-5

**Published:** 2021-02-08

**Authors:** Nitin Bayal, Sunil Nagpal, Mohammed Monzoorul Haque, Milind S. Patole, Yogesh Shouche, Shekhar C. Mande, Sharmila S. Mande

**Affiliations:** 1 National Centre for Cell Science, NCCS Complex, Pune, India; 2 BioSciences R&D, TCS Research, Tata Consultancy Services Ltd, Pune, India; 3 National Centre for Microbial Resources, NCCS, Pune, India; 4 Council of Scientific and Industrial Research, Anusandhan Bhawan, 2, Rafi Marg, New Delhi, India

**Keywords:** Data processing, DNA sequencing, Next-generation sequencing, Microbial communities, Skin diseases

## Abstract

Although skin is the primary affected organ in Leprosy, the role of the skin microbiome in its pathogenesis is not well understood. Recent reports have shown that skin of leprosy patients (LP) harbours perturbed microbiota which grants inflammation and disease progression. Herein, we present the results of nested Polymerase Chain Reaction-Denaturing Gradient Gel Electrophoresis (PCR-DGGE) which was initially performed for investigating the diversity of bacterial communities from lesional skin (LS) and non-lesional skin (NLS) sites of LP (n = 11). Further, we performed comprehensive analysis of 16S rRNA profiles corresponding to skin samples from participants (n = 90) located in two geographical locations i.e. Hyderabad and Miraj in India. The genus *Staphylococcus* was observed to be one of the representative bacteria characterizing healthy controls (HC; n = 30), which in contrast was underrepresented in skin microbiota of LP. Taxa affiliated to phyla Firmicutes and Proteobacteria were found to be signatures of HC and LS, respectively. Observed diversity level changes, shifts in core microbiota, and community network structure support the evident dysbiosis in normal skin microbiota due to leprosy. Insights obtained indicate the need for exploring skin microbiota modulation as a potential therapeutic option for leprosy.

## Introduction

Skin is the largest organ of human body and serves mainly as a physical barrier to protect the host from invading microorganisms and other environmental stresses^1,2^. Skin possesses microenvironment conducive for the growth of large number of microorganisms including bacteria, archaea, viruses, fungi and protists having symbiotic, saprophytic, commensal or opportunistic relationship. In addition to resisting infiltration by potential pathogens, these communities of microorganisms (collectively referred to as skin microbiota) also play diverse physiological roles that have a significant impact on the induction, training, and functioning of our immune system, response to external infections and physical injuries^3^. Increasing evidences in support for skin and microbiome links in health and disease have highlighted the importance of the skin microbiome and the essential role in physiology, immune responses and metabolism^4,5^. A number of recent studies have shown associations between compositional variations in skin microbiota with various types of dermatological disorders and pathological conditions like Psoriasis, Dermatitis, Eczema, Vitiligo, Leprosy etc^6–10^.

Among the mentioned disorders, leprosy in particular represents one of the most debilitating kind of skin infection that is present worldwide with approximately 250,000 new cases reported every year and around 2 million people suffer from disease-inflicted debilities^11^. Leprosy is associated with significant social stigma that renders the life of affected individuals generally quite difficult and isolated^12^. Leprosy is caused by *Mycobacterium leprae* or *Mycobacterium lepromatosis* with a chronic granulomatous infection of the peripheral nerves along with skin^13–16^. The disease is characterized by damage of the peripheral nerves, mucous membranes, eyes and skin. In lepromatous leprosy, the skin develops a large number of symmetrically distributed macules which are poorly defined with mild hypo-pigmentation and erythema^17^. Flesh-coloured erythematous papules and nodules may also be present on skin. Untreated skin especially on the face, thickens because of dermal infiltration giving rise to the “leonine facies”. While in tuberculoid leprosy, the skin develops one or a few lesions or plaques having well-defined edges. Hypo-pigmentation predominates over the erythema in case of the dark skin, while copper colour is usually observed in lighter skin^18^.

Two recent studies have revealed interesting aspects with respect to the structure of microbial communities present on LS sites or ‘affected skin’ and adjoining NLS or ‘unaffected’ skin of LP^19,20^. One of the objectives of these studies was to obtain a preliminary glimpse of bacterial genera as well as species that possibly modulate *M. leprae* infection on LS and NLS sites of LP. In the first study, analysis of the composition of skin microbiota in lepromatous LS (as well from adjoining NLS sites) was compared with similar skin sites of HC from Brazil population cohort^19^. The study also included comparison between the structure of microbial communities in samples taken from freshly diagnosed LP as well as those at various stages of multi-drug therapy regimen and post multi-drug therapy. Results indicated that irrespective of treatment, the diversity of skin microbiota in LP was significantly lower as compared to HC. The reduced microbial diversity observed in both treated and untreated individuals appeared to indicate either (a) a possible systemic level impact of the *M. leprae* intra-dermal infection on other co-inhabiting members of the skin microbial community or (b) changes occurring as a result of the ongoing therapeutic regimen.

The second study (published recently), put forward data (along with some preliminary analysis) corresponding to the structure of skin microbial communities in HC (n = 30) as well as LP (n = 60) from India^20^. It is pertinent to note here that 31,666 active leprosy cases have been identified in the year 2016 in India, out of which 3755 cases were in the paediatric age^21^. To account for variability arising as a result of various factors like food preferences, climatic differences and varying lifestyles within different regions of India, study participants (in the second study) were chosen from well-established leprosy research centres located in two geographically well-separated locations i.e. Hyderabad and Miraj from India. Both the mentioned centres offer leprosy treatments at the regional level and treatment protocols in both centres are followed in accordance with World Health Organisation guidelines.

In this study, we present results of nested PCR-DGGE and comprehensive computational analysis for the microbial diversity exploration present in skin swab samples collected from LS and NLS skin sites of LP provided that the information pertaining to the datasets is available in an earlier report^20^. Sequence data corresponding to the second research study is currently accessible at NCBI Sequence Read Archive (SRA) as BioProject: PRJNA505133 (Study: SRP187334). The overall objective was to catalogue and compare the compositional makeup of skin microbial communities present in HC with those on LS and NLS sites of LP. Such similarities or differences, if any, can potentially be used for generating preliminary hypotheses about the kind of interactions existing between disease causing microorganisms, other microbial co-inhabitants and the patho-physiology of the disease in LS.

## Results

**Figure 1:**
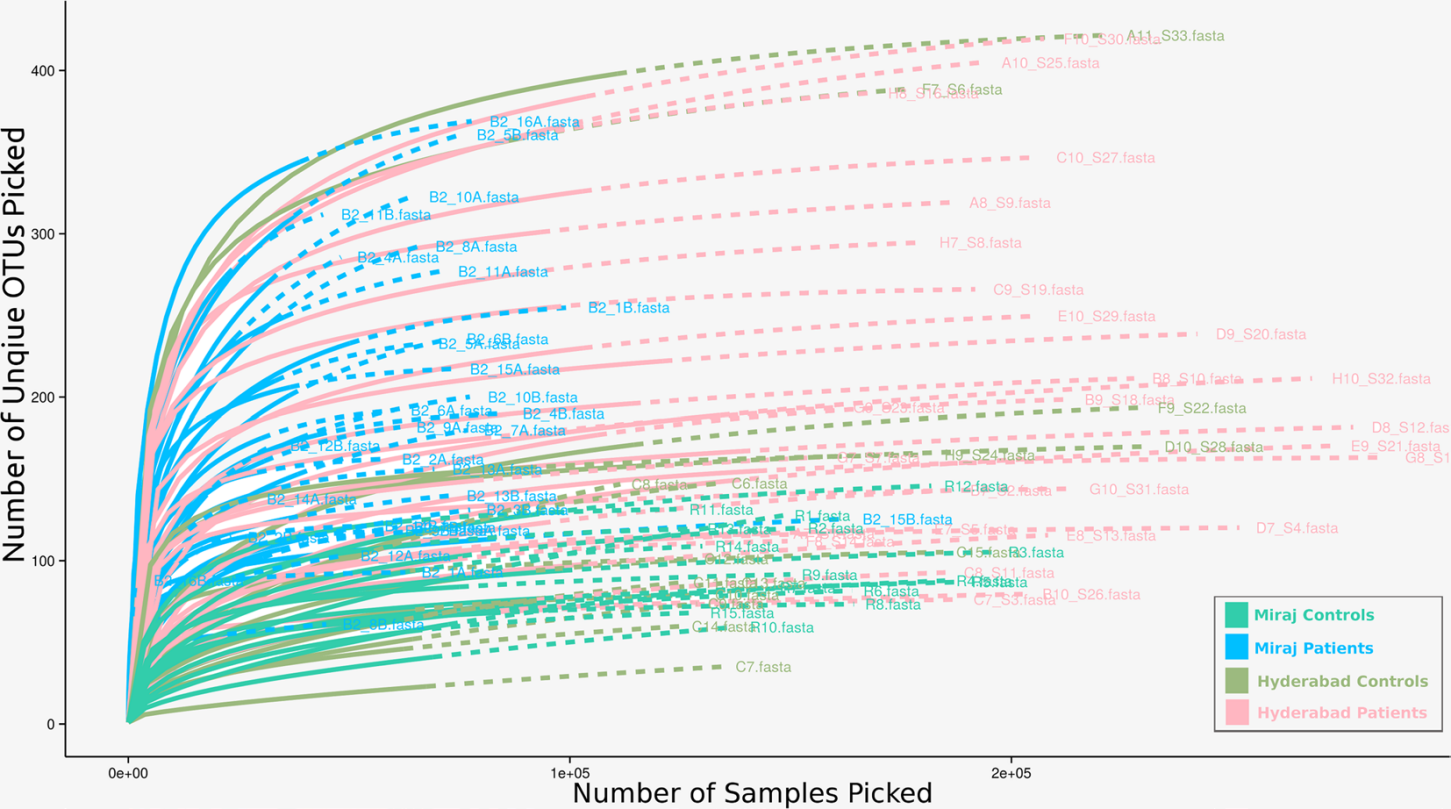
Rarefaction analysis of samples used in the present study. Rarefaction plot for all 88 samples analysed in the present study. Plot indicates reasonable sequencing coverage for most samples.

A comprehensive analysis was carried out on the available 16S rRNA gene sequencing datasets corresponding to 88 skin swab samples, comprising of 30 samples taken from HC (15 each from Hyderabad and Miraj) and a total of 58 samples from LP from both sampling locations. We had provided information pertaining to the datasets in an earlier report^20^. For convenience and reference, the microbial taxonomic abundance profiles have been (re)provided in Supplementary Files 1. Preliminary rarefaction analysis (Fig. 1) indicates sufficient sequencing depth for most of the samples. As a secondary validation, Good's coverage index was computed for all samples (Supplementary Table 1). Values for genus level abundance matrix i.e. ≥ 0.99 and OTU abundance matrix i.e. ≥ 0.90 were obtained for majority of the samples. Good’s coverage for few samples from Miraj indicated additional scope for sequencing. Detailed insights of the obtained results while addressing a few questions are provided as follows.

**Table 1:** Common differentiating genera between controls and patients from both geographies.

S.No.	Genus	BH corrected p-value < 0.05				
		Median abundance				
		Control Hyd	Patient Hyd	Control Miraj	Patient Miraj	Trend*
1	Staphylococcus	52.7520	3.8100	67.5150	1.9680	▼
2	Pseudomonas	0.6430	32.1445	0.0030	0.1020	▲
3	Paracoccus	0.9540	4.2155	0.1590	0.5775	▲
4	Acinetobacter	0.3350	0.7075	0.0030	1.1950	▲
5	Micrococcus	0.1400	0.5785	0.0840	1.3105	▲
6	Bacillus	0.0100	0.0945	0.0120	1.3855	▲
7	Enhydrobacter	0.0000	0.0755	0.0000	0.4515	▲
8	Limnobacter	0.0000	0.3605	0.0000	0.0205	▲
9	Gulbenkiania	0.0220	0.0000	0.2820	0.0000	▼
10	Janibacter	0.0020	0.0355	0.0080	0.1065	▲
11	Ornithinimicrobium	0.0010	0.0530	0.0040	0.0840	▲
12	Brevibacterium	0.0050	0.0495	0.0120	0.0540	▲
13	Solimonas	0.0000	0.1120	0.0000	0.0025	▲
14	Mycobacterium	0.0020	0.0645	0.0000	0.0200	▲
15	Delftia	0.0000	0.0160	0.0000	0.0680	▲
16	Alishewanella	0.0000	0.0055	0.0000	0.0635	▲
17	Dietzia	0.0050	0.0380	0.0030	0.0215	▲
18	Sphingomonas	0.0030	0.0275	0.0010	0.0245	▲
19	Serinicoccus	0.0000	0.0100	0.0000	0.0460	▲
20	Microbacterium	0.0020	0.0185	0.0050	0.0245	▲
21	Bosea	0.0070	0.0000	0.0280	0.0000	▼
22	Curvibacter	0.0000	0.0045	0.0000	0.0305	▲
23	Aeromonas	0.0000	0.0120	0.0000	0.0170	▲
24	Rhizobium	0.0020	0.0130	0.0050	0.0005	▲
25	Rheinheimera	0.0000	0.0105	0.0000	0.0070	▲
26	Ideonella	0.0000	0.0020	0.0000	0.0080	▲
27	Thermomonas	0.0000	0.0045	0.0000	0.0045	▲
28	Rhizorhapis	0.0000	0.0010	0.0000	0.0060	▲
29	Piscinibacter	0.0000	0.0005	0.0000	0.0015	▲

*Trend refers to the relative change in leprosy patients, as compared to healthy controls.

### Denaturating Gradient Gel Electrophoresis (DGGE) experiments indicate differences between microbial community structure in LS and NLS sites

DGGE is a molecular sequence dependent fingerprinting technique that allows characterization of the microbiota from large number of skin samples without pre-existing knowledge of its composition. The technique allows visual comparison of communities based on the number and position of DNA bands in DGGE gel. Image analysis of DGGE banding patterns also gives a phylogenetic relationship between different samples. Therefore, in this study, initial experiments were done to find out the differences in the microbiota between LS and NLS sites of LP. Skin samples obtained from a total of 11 LP (five from Hyderabad and six from Miraj) were analysed using DGGE technique. A representative DGGE gel image of PCR products obtained from amplification of total DNA extracted from skin swabs of LS and NLS of five LP from Hyderabad city is shown in Supplementary Fig. 1. The gel image showed that each subject group has distinct banding pattern and *Rf* values. DNA was extracted from a total of 196 distinct bands excised from DGGE gel. The PCR bands were selected for identification by Sanger DNA sequencing method. The sequences obtained were analysed using EZTaxon server (http://www.eztaxon.org/) to ascertain their closest bacterial species relative(s). A total of 169 sequences were identified up to species level (Supplementary Table 2). While the NLS microbiota for LP from both Hyderabad and Miraj indicated the presence of similar microbiota with bacteria belonging to genus *Canibacter*, *Corynebacterium*, *Cutibacterium*, *Janibacter*, *Moraxella* and *Staphylococcus,* samples of LP from Miraj were observed to additionally harbour *Bacillus*, *Methylobacterium*, *Microvirga*, *Paracoccus* and *Staphylococcus*. While LS sites were seen to have a more diverse microbial community as compared to that in NLS sites, there were few differences in the community structure for Hyderabad and Miraj subjects (Supplementary Table 2). Some of the important genera found in Hyderabad subjects were *Canibacter*, *Corynebacterium*, *Cutibacterium*, *Janibacter*, *Moraxella* and *Staphylococcus* while Miraj subjects had *Bacillus*, *Methylobacterium*, *Microvirga*, *Paracoccus* and *Staphylococcus* genera.

Although DGGE experiments showed differences between the microbial communities in LS and NLS sites, it is well known that the taxa identified by this technique typically constitute less than one percent of total bacterial community. Moreover, DGGE is a semi-quantitative technique which may result in generating multiple bands for the same species and also in some cases it may result in formation of hetero-duplexes thereby causing bias in analysis. In view of these limitations, Illumina MiSeq based Next generation sequencing for the V1–V3 region of 16S rRNA gene was performed to obtain detailed quantitative and reproducible taxonomic information about skin microbiota in the collected samples.

### Diversity and composition of skin microbiota in skin samples from HC and LP (analysed using 16S amplicon sequencing data)

#### (a) Beta diversity analysis indicates conserved microbial community structure in HC and geography-specific signatures in LP

**Figure 2:**
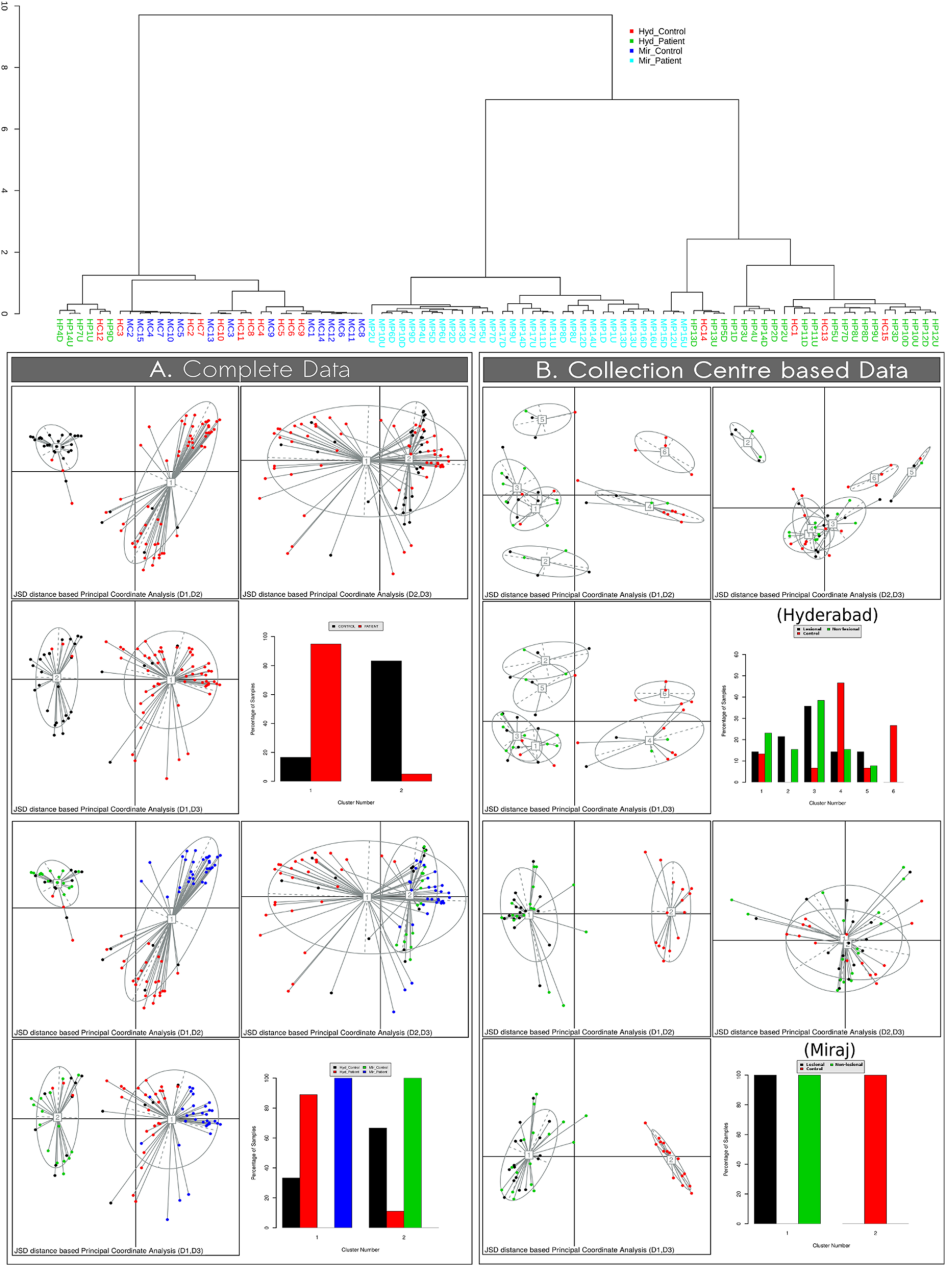
Principal component analysis (PCoA) of control, lesional and non-lesional bacterial communities. PCoA clustering of microbial abundance profiles (corresponding to all 88 samples) based on Jensen–Shannon (JSD) divergence metric. Results in **(A)** indicate formation of two clusters. Samples corresponding to controls (healthy participants) observed to group together in the same cluster irrespective of the geographical location (top portion of **A**). Samples from both LS and NLS sites of LP were observed to group together in the same cluster (which is spatially separated from the cluster having the control samples (bottom portion of **A**). LP samples were observed to spatially group together based on sampling location (Hyderabad or Miraj). A cladogram generated using Ward clustering is depicted in **(A)**. This indicates the extent of individual level similarities amongst samples from both geographic locations. Results in **(B)** indicate absence of unique microbiota signatures in LS and NLS sites of LP.

Given that samples were collected from two different geographical locations in India, we first performed ordination analysis of corresponding microbial abundance profiles in order to evaluate the pattern of beta-diversity that characterize various groups of samples from Hyderabad and Miraj (i.e. control samples from HC and patient samples from LP, both LS and NLS). Microbial abundance profiles (corresponding to all 88 samples) were clustered using principal coordinate analysis (PCoA) using Jansen-Shannon distance (JSD) metric. Interestingly, results indicate formation of two clusters, wherein samples corresponding to controls (HC) were observed to group together in the same cluster irrespective of the geographical location (Fig. 2, top portion of panel A). Although samples from both LS and NLS sites of LP were observed in the same cluster which is spatially separated from the cluster having HC samples, LP were observed to spatially group together based on their sampling location as well (Fig. 2, bottom portion of panel A). While the clustering of HC samples (irrespective of their geography) seems to suggest relatively higher uniformity in community structure of resident skin bacterial communities, the geography based spatial segregation of LP samples not only points towards inherent dysbiosis in skin microbiota due to leprosy, but also distinct geography-driven pattern of dysbiosis. The relatively tight grouping of HC samples as compared to the observed LP samples also seems to align closely with the Anna Karenina principle for animal microbiomes^22^.

As a next logical step, PCoA was performed with the aim of separately analysing the pattern of clustering within samples from Hyderabad or Miraj locations. The primary objective was to check existence of unique signatures for microbial abundance profiles corresponding to samples taken from LS and NLS sites of LP. Results depicted in Fig. 2 (panel B) and Supplementary Figs. 2 and 3 indicate an apparent absence of any unique signature (in both geographical locations). This seems to suggest that dysbiosis of the skin microbial community due to leprosy probably brings in a systemic level change in the cutaneous microbiota of the LP and appearance of characteristic lesions are likely manifestation of other local patho-physiological changes that accompany infection by the *Mycobacterium* pathogen. In this context, it is strikingly noted that the Ward clustering method based on Jensen-Shannon divergence (JSD) to cluster the observations of microbial taxonomic profiles generates a cladogram that shows individual level similarities amongst samples from both geographical locations (Fig. 2; Top panel).

**Figure 3:**
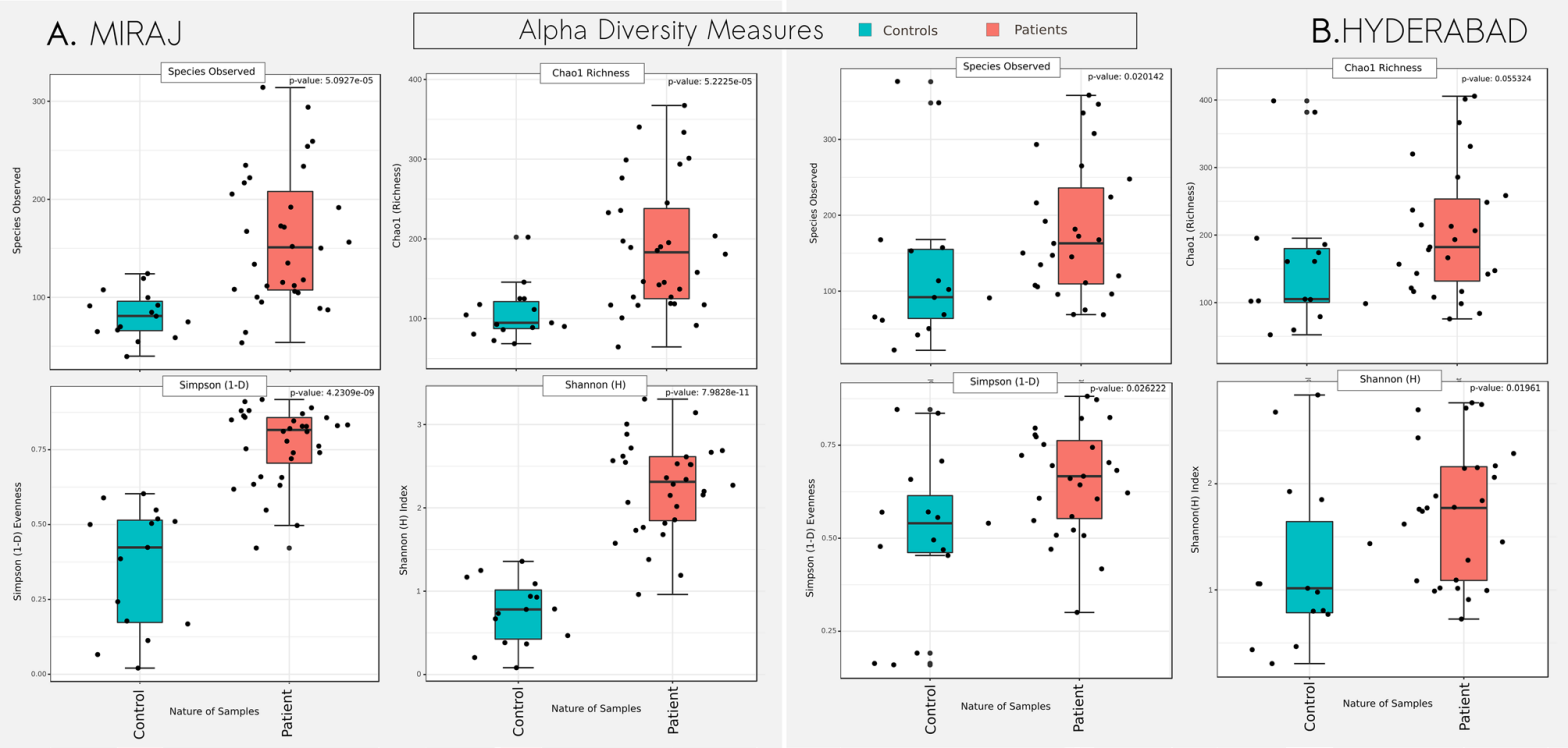
Alpha-diversity trends in skin microbiota samples obtained from healthy controls and leprosy patients. Box plots illustrating the comparison of diversity indices (S-Obs, Chao-1, Simpson1-D and Shannon) between skin microbiota samples obtained from HC and LP from Miraj (**A**) and Hyderabad (**B**).

#### (b) Alpha diversity analysis indicates lower species richness, evenness and overall diversity in HC as compared to LP

In order to check if leprosy infection impacts the microbial diversity of skin at overall community level, alpha-diversity metrics were generated for all samples and their comparisons were performed with two logical viewpoints. We first checked whether differences in diversity exist between samples taken from HC and LP (irrespective of geographical location of sample collection). Results from the analysis (Fig. 3) clearly indicate a statistically significant difference between diversity metrics computed from the set of HC and those from LP samples. Results further indicates that skin microbiota of HC possesses lower species richness, evenness and overall diversity. This observation is found to be consistent for both Hyderabad and Miraj datasets.

**Figure 4:**
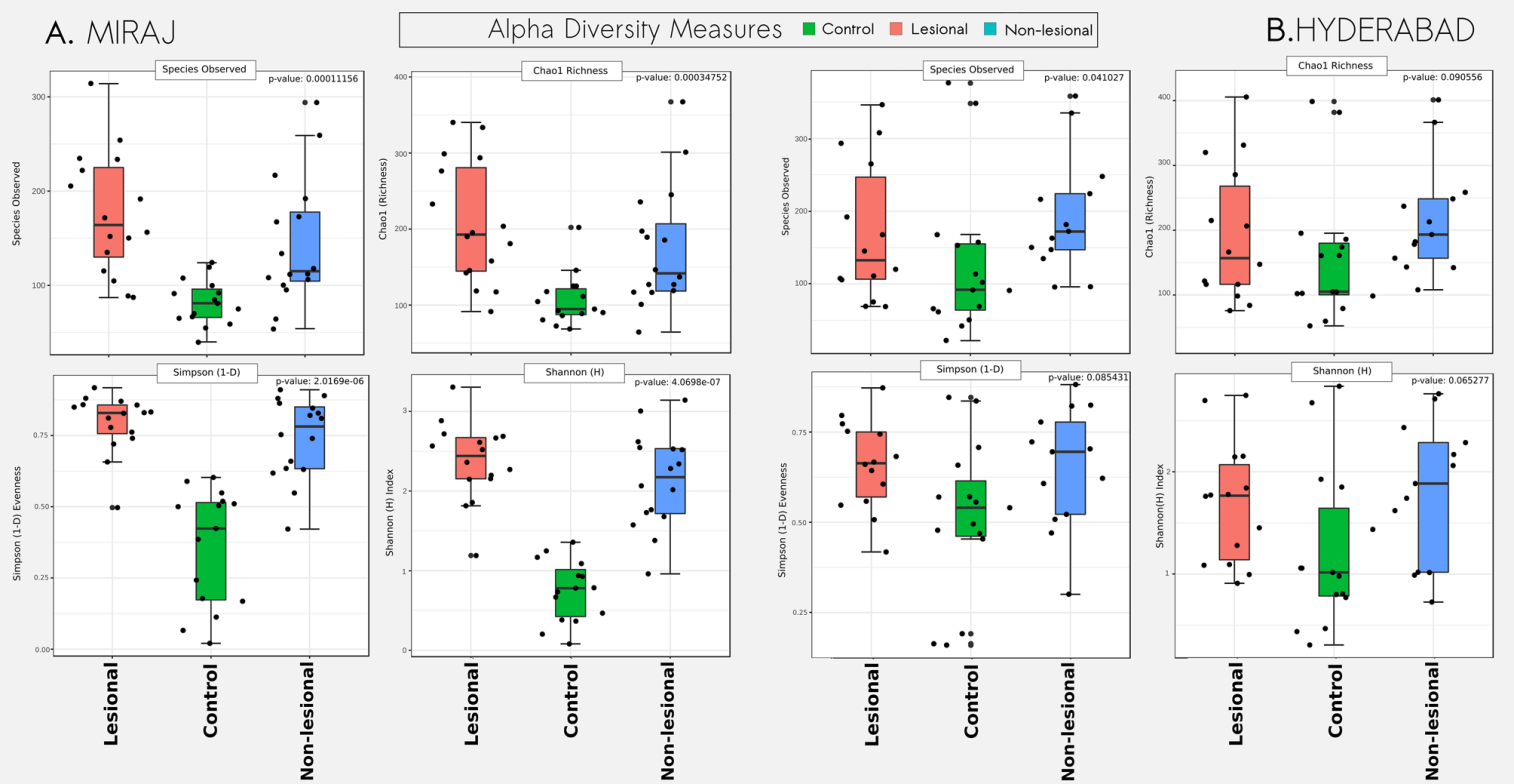
Alpha-diversity trends in microbiota samples obtained from healthy controls and lesional and non-lesional skin sites of leprosy patients. Box plots illustrating the comparison of diversity indices (S-Obs, Chao-1, Simpson1-D and Shannon) between skin microbiota samples obtained from HC and LS and NLS sites of LP from Miraj **(A)** and Hyderabad **(B)**.

Secondly, similar analysis was subsequently performed on microbial abundance profiles of HC with specific consideration to both LS and NLS samples from LP. Analysis (performed with samples from Hyderabad and Miraj) indicate that cutaneous microbiota of HC has significantly lower diversity as compared to samples from both LS and NLS of LP (Fig. 4). It may be noted that the differences between diversity metrics of paired samples i.e. LS and NLS of LP did not exhibit a statistically significant difference.

#### (c) Taxonomic analysis indicates presence of few taxa having distinct abundance trends between HC and LP (as well as between LS and NLS sites of LP)

Despite the distinct spatial clustering pattern for microbial abundance profiles of samples obtained from study participants in two geographical distinct locations, the profiles depict a reasonable amount of similarity with respect to taxa whose abundances are consistently high in respective samples and sample groups. *Acinetobacter*, *Corynebacterium*, *Kocuria*, *Micrococcus*, *Paracoccus*, *Propionibacterium*, *Staphlococcus* were observed to be the most abundant common taxa in samples from both Hyderabad and Miraj. All mentioned 'abundant' taxa were identified based on their relative median abundances in the respective sample sets.

#### (i) Skin microbial community composition specific to Hyderabad

**Figure 5:**
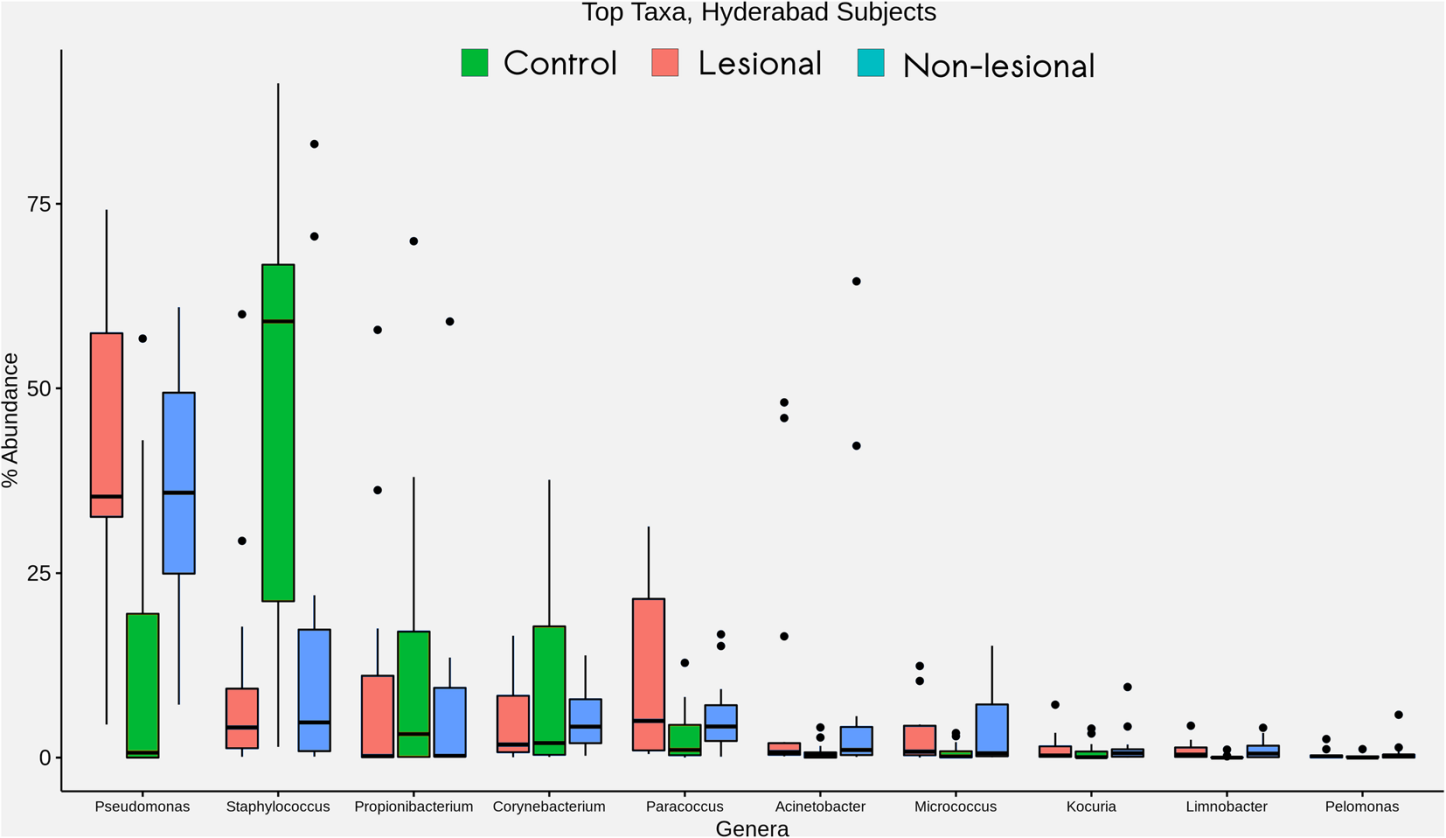
Taxonomic composition of cutaneous microbiome in study participants from Hyderabad. Box plots representing relative abundance analysis of most abundant bacterial taxa discovered in samples obtained from HC, LS and NLS sites of LP in Hyderabad.

Results from the present analyses (Fig. 5) indicate major differences between the abundance distribution pattern of *Brevundimonas*, *Limnobacter*, *Paracoccus*, *Pseudomonas*, *Staphlococcus* and *Streptococcus* in samples from HC and LP (both LS and NLS). *Limnobacter*, *Paracoccus* and *Pseudomonas* were observed to have a relatively higher abundance in skin microbiota of LP (both LS and NLS) as compared to those in HC. An opposite trend is observed for *Brevundimonas, Staphylococcus and Streptococcus,* that have higher relative abundances in samples collected from HC. At a higher taxonomic level, samples were observed to have taxa belonging to the following phyla—Actinobacteria, Bacteroidetes, Cyanobacteria, Deinococcus-Thermus, Firmicutes and Proteobacteria. Interestingly, the abundance pattern of phyla Proteobacteria and Firmicutes were observed to exhibit a completely opposite trend between samples collected from HC and LP (both LS and NLS).

#### (ii) Skin microbial community composition specific to Miraj

**Figure 6:**
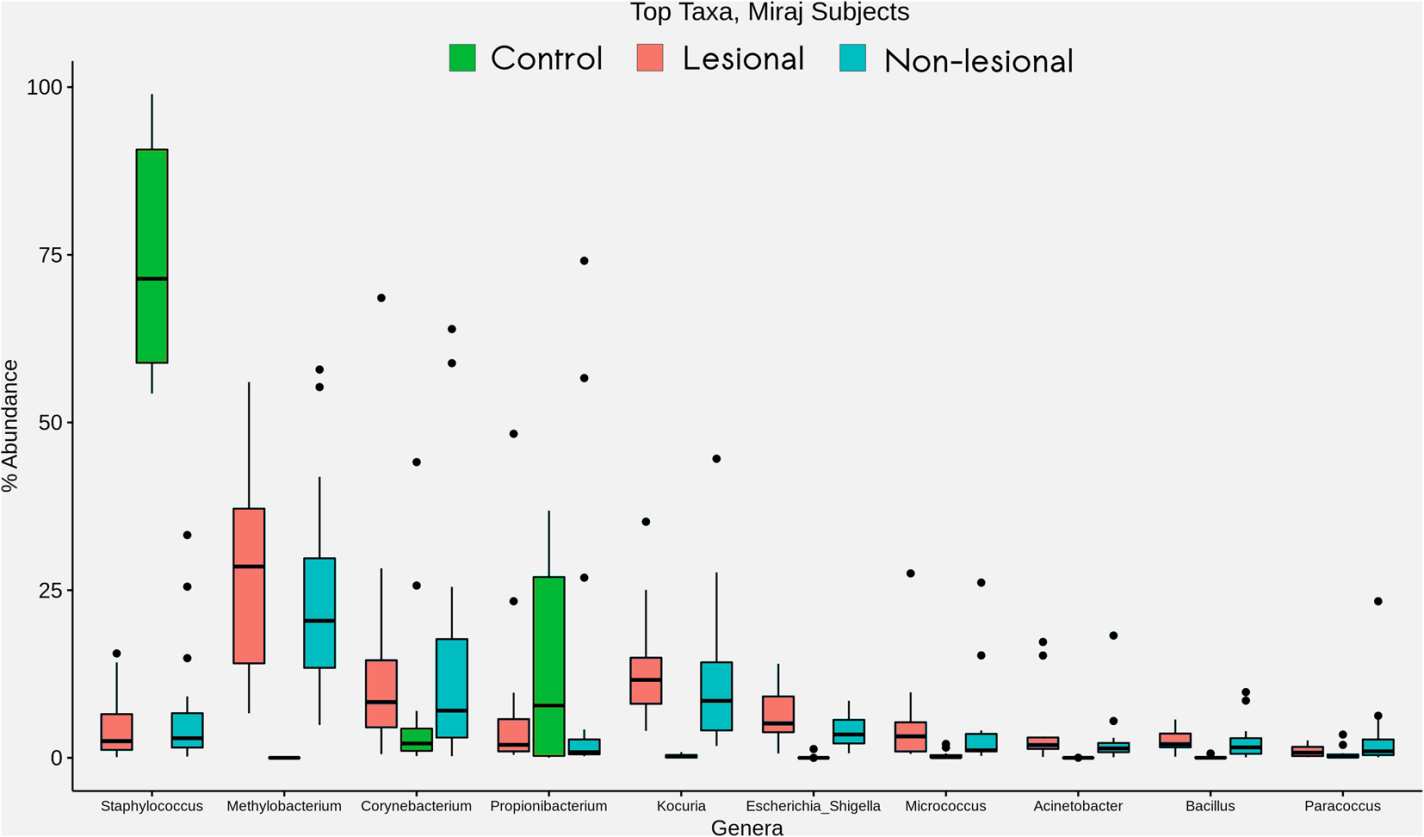
Taxonomic composition of cutaneous microbiome in study participants from Miraj. Box plots representing relative abundance analysis of most abundant bacterial taxa identified in samples obtained from HC, LS and NLS sites of LP in Miraj.

Major differences are observed between the abundance distribution pattern of *Corynebacterium, Kocuria, Methylobacterium, Propionibacterium and Staphylococcus* in samples from HC and samples from Miraj LP (both LS and NLS sites) (Fig. 6)*.* All mentioned taxa, except for *Staphylococcus* and *Propionibacterium,* are observed to have a relatively higher abundance in skin microbiota of LP (both LS and NLS sites) as compared to those in HC. An evident depletion in abundance of *Staphylococcus* genus has consistently been observed in samples from all the LP samples i.e. both LS and NLS.

Overall, the observed general composition of skin microbiota in the studied samples are consistent with the available literature reports for the skin microbiota^23^. Taxa belonging to the phyla Actinobacteria, Bacteriodetes, Firmicutes and Proteobacteria have been known to majorly constitute the skin microbiome that includes *Brevundimonas, Corynebaterium, Kocuria, Limnobacter, Paracoccus, Pseudomonas and Staphylococcus genera* to constitute a significant proportion of skin microbiota^24^.

### Core microbes identified in skin microbiota of HC and LP (as well as between LS and NLS sites of LP)

**Figure 7:**
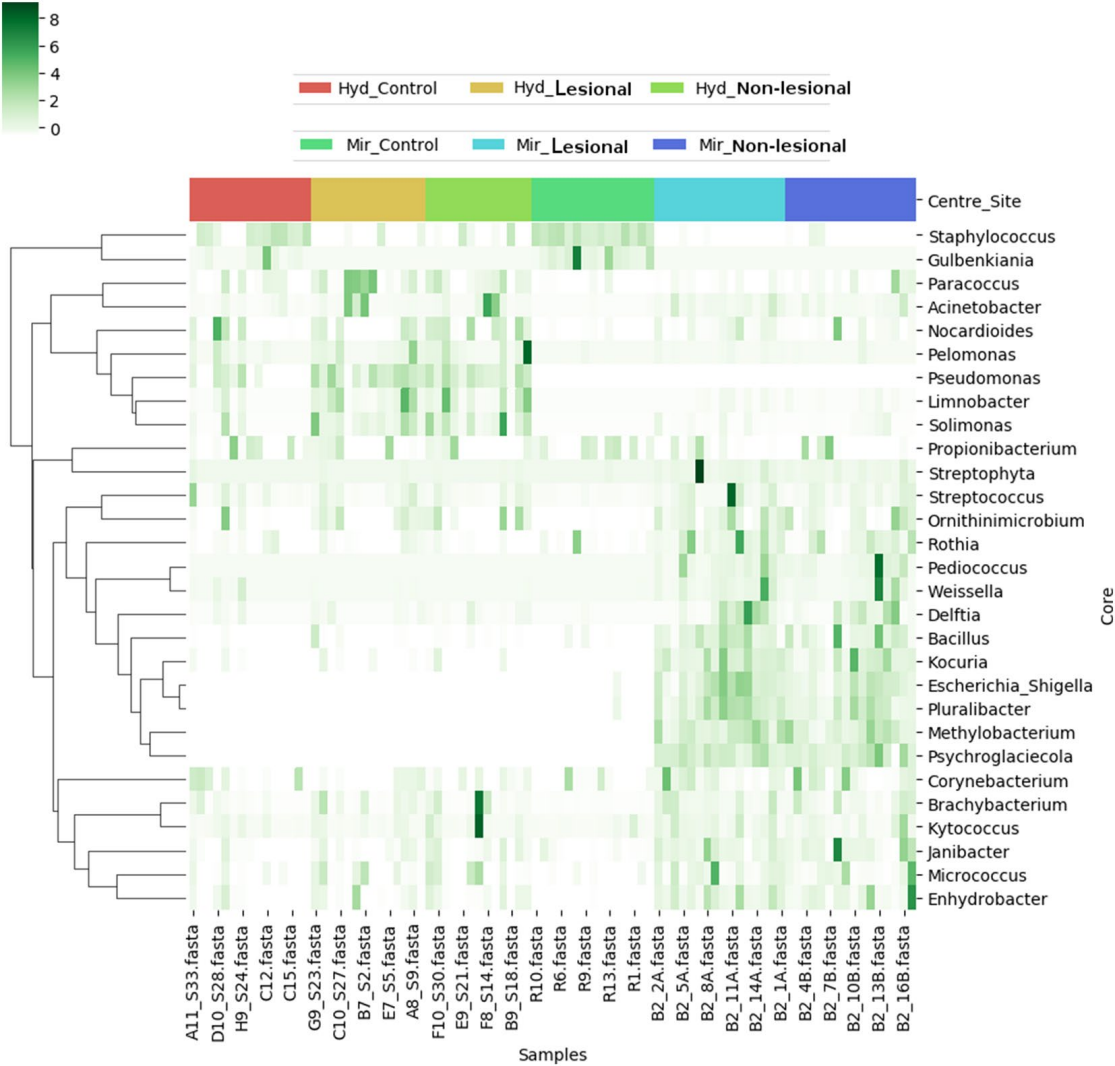
Core bacterial genera within and across various sample sets in both geographies. Core bacterial genera identified in samples obtained from HC, LS and NLS sites of LP from Hyderabad and Miraj. These genera were observed to be present in ≥ 70% of the samples in the respective sample set with minimum 0.1% relative abundance.

Core microbes within and across various sample sets in both geographies were ascertained using the previously described procedure^6^. Results depicted in Fig. 7 indicate a distinct set of core microbes characterising Hyderabad and Miraj specific samples respectively. The taxa *Brachybacterium, Corynebacterium, Enhydrobacter, Janibacter, Kytococcus, Micrococcus and Propionibacterium* (the branch at the lower end of the dendrogram depicted in Fig. 7) are nevertheless observed to constitute the common core for all samples irrespective of geographical location. Interestingly, *Gulbenkiana* and *Staphylococcus* are observed to constitute the dominant 'HC specific' core taxa in the skin microbiota of samples from both the locations. The abundances of two genera are distinctly reduced in the skin microbiota of all the LP samples. Furthermore, while *Acinetobacter, Paracoccus and Pseudomonas* constitute the patient-specific dominant core for Hyderabad subjects, *Bacillus* and *Kocuria* were observed to be dominant core taxa in subjects from Miraj. It is apposite to consider that the common set of core taxa between HC and LP represent previously reported skin-associated microbial taxa^6,19^. Taxa that were consistently present at very low abundance (0.001%-0.1% in at least 75% samples, i.e. rare core taxa) were also identified (Supplementary Figs. 4 and 5). A total of 40 taxa were identified to constitute the rare core taxa, with *Methylobacterium, Brevundimonas, Brachybacterium, Streptophyta, Delftia, Janibacter, Deinococcus, Brevibacterium, Anaerococcus, Nocardioides, Luteimonas, Roseomonas, Rubellimicrobium, Mycobacterium* being the top 15 population level core rare taxa across samples.

### Statistical analysis reveals presence of disease- and geography-specific taxonomic signatures in skin microbiota of HC and LP

**Figure 8:**
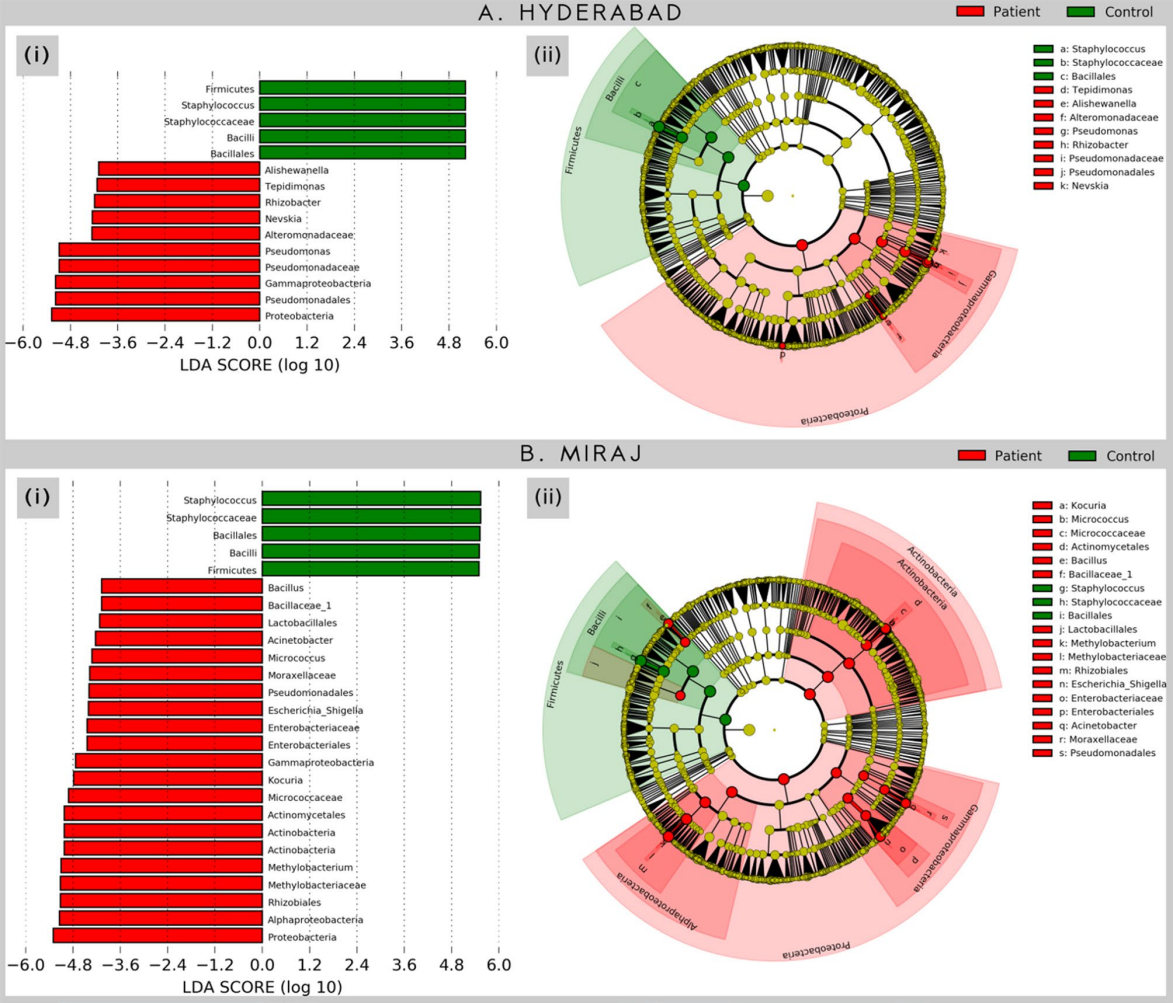
Taxa identified by LefSe to have significantly different abundance between various sample groups. Taxa with significantly different abundance (identified using LEfSe). Taxa with significantly different abundance were identified using an LDA cut-off of > 4 at a p-value of 0.05. The cladogram in this figure illustrates the phylogenetic relationship amongst the significantly differentiating microbial taxa.

To identify disease and/or geography-specific bacteria, we analysed respective sample sets using LefSe^55^ package (LDA effect size with LDA cut-off > 4; Wilcoxon p value cut-off of 0.05). Results indicate 15 taxa (including 6 genera, 3 families, 2 orders and 2 classes, 2 phyla) to have a statistically significant difference in their relative abundance between the compared sample groups in datasets pertaining to Hyderabad group. A much larger set of differentiating taxa is observed for Miraj group (26 taxa). Figure 8 represents these details pertaining to taxa identified by LefSe. A common aspect to skin microbiota composition in samples from all the LP samples is the significant depletion of taxa that belong to phylum Firmicutes and a significantly enhanced abundance of taxa from Proteobacteria phylum (irrespective of geographical location). The relative abundance of *Bacillales*, *Bacilli* and *Staphylococcus* (respectively) were observed to be significantly depleted in the skin microbiota of LP from both Hyderabad and Miraj for order, class and genus levels. In addition, the abundances of taxa corresponding to order Pseudomonadales were observed to be have statistically significant higher abundance in LP samples from both the locations. Supplementary Figs. 6 and 7 depict the results of LefSe obtained by comparing microbial abundance profiles corresponding to LS and NLS of LP from Hyderabad and Miraj respectively. The genera *Gaiella*, *Legionella*, *Pseudonocardia* and *Sphinobium* are identified to have a significant difference in their abundances between the LS and NLS of LP from Hyderabad. Although these genera are observed to have a low abundance (< 0.05%), they seem to be significantly over-represented in LS as compared to NLS (Supplementary Fig. 6). In the case of sample sets corresponding to subjects from Miraj, the only common genus observed to have a significantly different abundance (in LS versus NLS sample sets) is *Pseudonocardia*. Interestingly, in contrast to the results described for Hyderabad, most of the genera observed to be differentiating between LS and NLS of Miraj subjects are not sparsely abundant. They rather have a reasonably good abundance (0.5–4%) in the analysed samples (Supplementary Fig. 7). Table 1 provides a summary of the genera observed to have significantly different median abundance (BH corrected p-value < 0.05) between controls and patients (common in both geographies). As apparent, while *Staphylococcus, Gulbenkiania and Bosea* are observed to be depleted in LP, all other genera exhibit an increase in their abundance on the skin of LP as compared to that of HC. Notable dominating taxa of the latter group pertain to *Pseudomonas, Paracoccus, Acinetobacter and Micrococcus, while Bacillus, Enhydrobacter, Limnobacter, Janibacter, Ornithinimicrobium, Brevibacterium, Solimonas and Mycobacterium* pertain to the sparse or rare differentiating taxa. Supplementary Fig. 8 represents a box-plot view for the common differentiating taxa as summarized in Table 1.

### Network analysis reveals signature differences in microbial co-occurrence patterns in data sets corresponding to HC and LP

**Figure 9:**
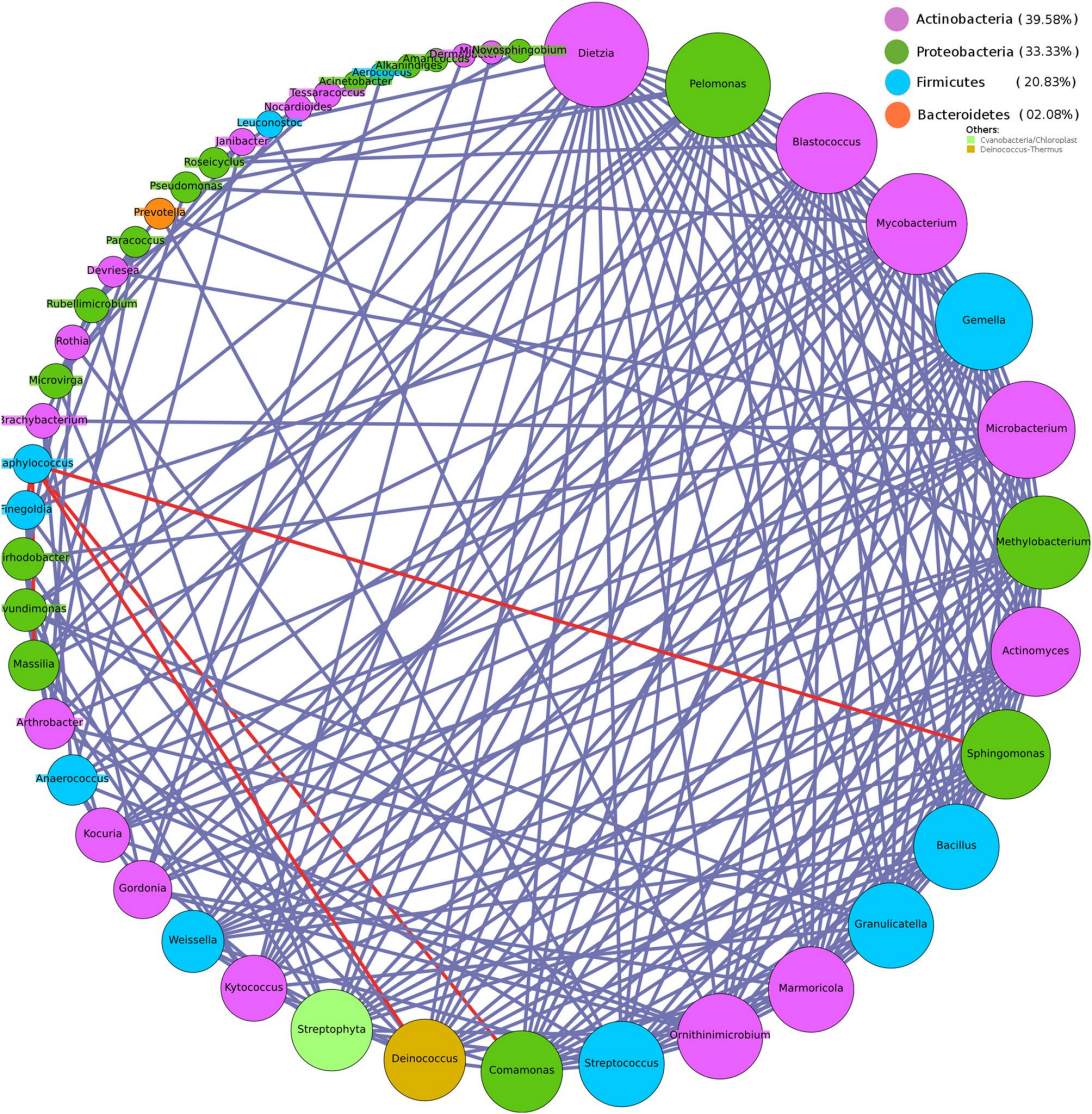
Degree sorted community network structure for samples pertaining to Healthy (control) set from Hyderabad location. Nodes were colored according to their phylum affiliation. Genera belonging to Actinobacteria and Proteobacteria phyla were not only observed to predominate the network in terms of membership but were also observed to be the top degree nodes of the co-occurrence network. Graphs were generated using Gephi v 0.9.2.

**Figure 10:**
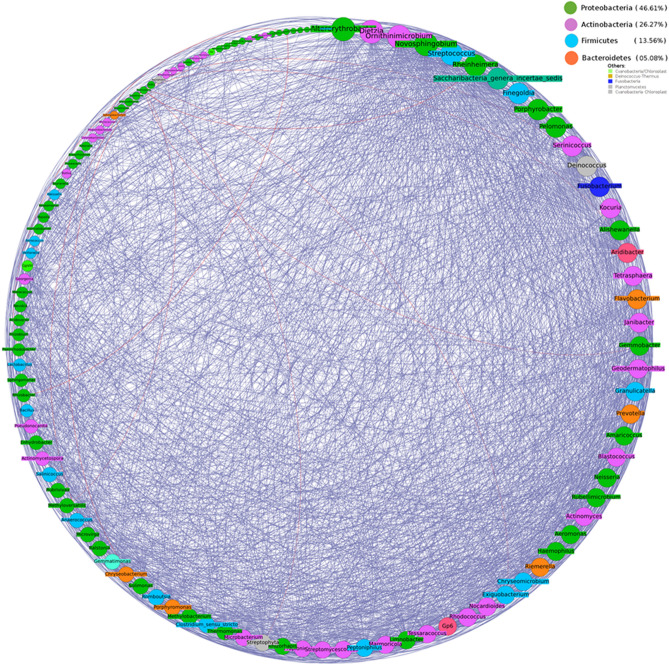
Degree sorted community network structure for samples pertaining to Leprosy affected individuals from Hyderabad location. Nodes were colored according to their phylum affiliation. Genera belonging to Proteobacteria phylum were observed to predominate the network in terms of membership (> 45% members belonged to Proteobacteria). Top degree nodes of this co-occurrence network were observed to be affiliated to Proteobacteria, Firmicutes and Actinobacteria. This network was observed to be highly dense in terms of the total edges, as opposed to the network pertaining to Control set from the same location. Graphs were generated using Gephi v 0.9.2.

**Figure 11:**
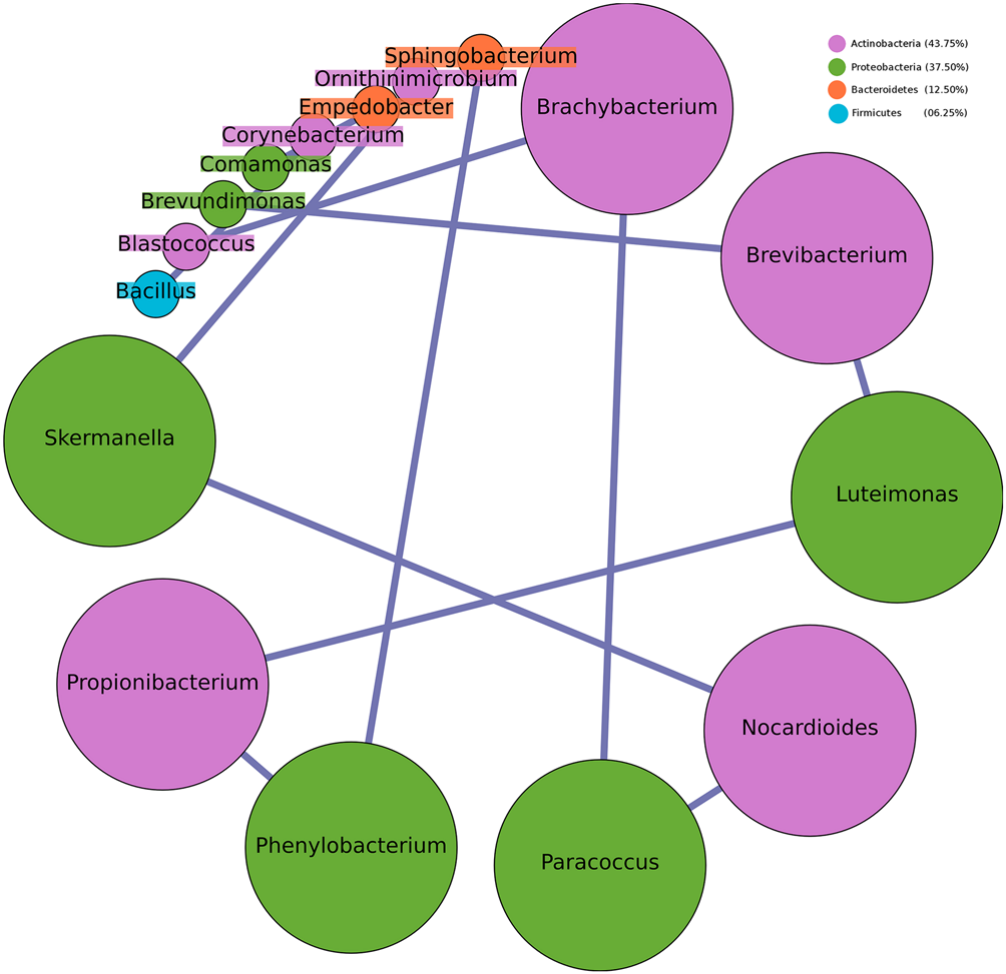
Degree sorted community network structure for samples pertaining to Healthy (control) set from Miraj location. Nodes were colored according to their phylum affiliation. Genera belonging to Actinobacteria phylum were observed to predominate the network in terms of membership. Top degree nodes of the co-occurrence network were observed to be affiliated to Actinobacteria and Proteobacteria. Graphs were generated using Gephi v 0.9.2.

**Figure 12:**
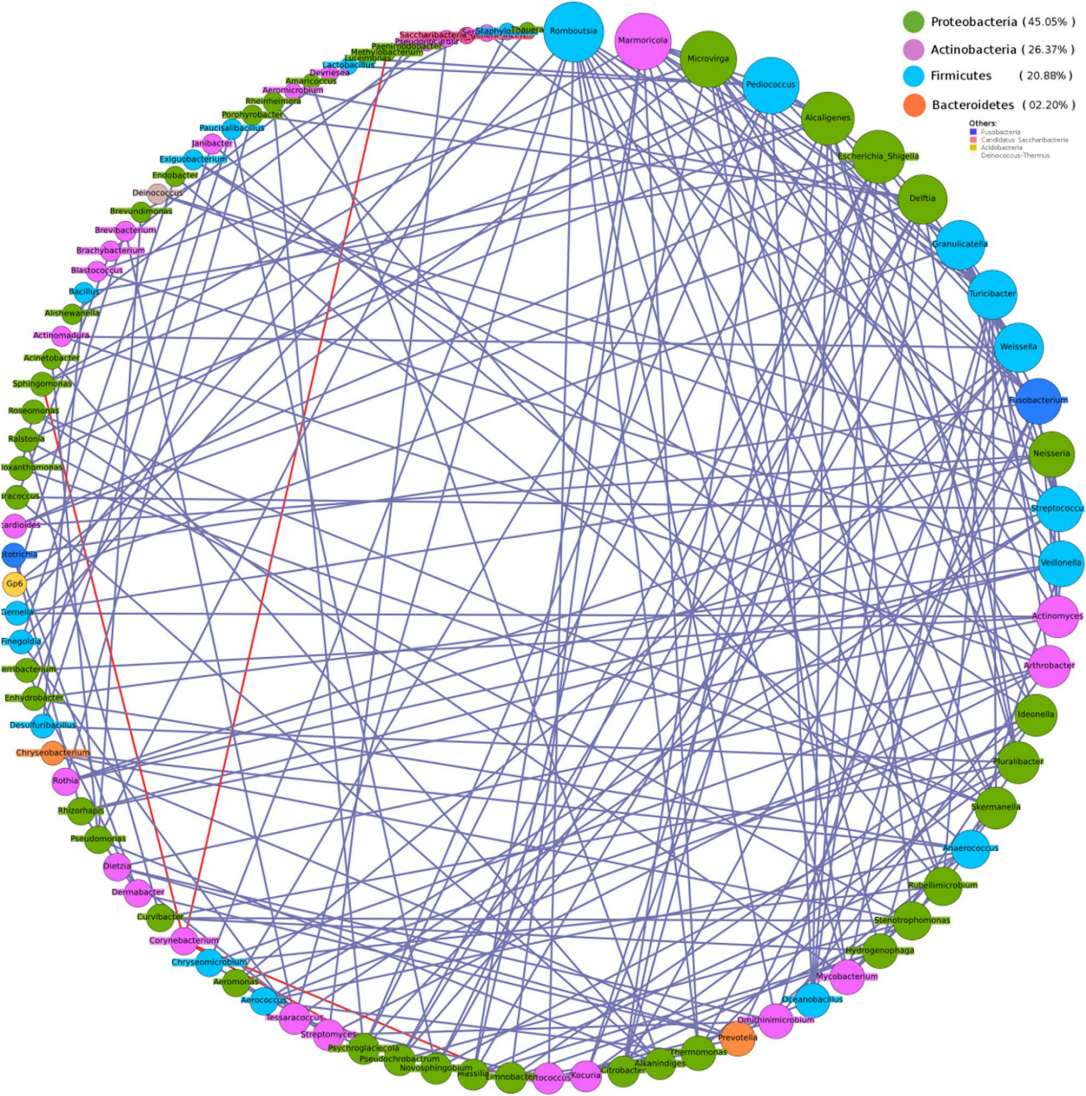
Degree sorted community network structure for samples pertaining to Leprosy affected individuals from Miraj location. Nodes were colored according to their phylum affiliation. Genera belonging to Proteobacteria phylum were observed to predominate the network in terms of membership. Top degree nodes of the co-occurrence network were observed to be affiliated to Firmicutes and Proteobacteria. This network was observed to be relatively dense in terms of the total edges, as opposed to the network pertaining to Control set from the same location. Graphs were generated using Gephi v 0.9.2.

Intra-community network analysis was performed to evaluate correlations between individual members of the microbial community and to assess potential associations between them. Various network properties (nodes, edges, density, diameter etc.) and centrality measures (degree and betweenness) of the resulting networks were analysed and compared. Results generated by individual analysis of data from both Hyderabad and Miraj primarily indicate an overall increase in network density in co-occurrence networks built using microbial taxonomic profiles corresponding to samples from LP as compared to similar networks built using samples from HC (Figs. 9, 10, 11, 12). A comparison of networks built individually using datasets corresponding to HC and LP samples collected from Hyderabad indicate an apparent shift of overall membership of network community from Actinobacteria and Proteobacteria in controls to predominantly Proteobacteria in LP from Hyderabad (Figs. 9 and 10). Similar analysis of samples from Miraj location, exhibit similar trend, wherein the phylum affiliation of most of the community members pertaining to the control set is observed to be Actinobacteria (Fig. 11). Co-occurrence network membership is observed to be dominated by Proteobacteria in the LP (Fig. 12). An observation also pertains to stark reduction in degree of interactions of the genus *Staphylococcus* in subject specific networks of Hyderabad location. Owing to small sample size for site specific samples for each geographical location, network analysis for LS and NLS samples were avoided.

## Discussion

The proportion of new cases of leprosy that involves disfigurement ranges from 6 to 21% and causes for this disability were inflammation and ulceration^25^. One of the noteworthy reasons of skin inflammatory reaction in LP was shown to be correlated with the presence of certain opportunistic bacteria on the skin^26^. Normal skin microbiota and its dysbiosis is now known to play an important role in different cutaneous inflammatory and infectious diseases^4,5^.

*M. leprae *infection is established by adhesion, invasion and proliferation of Schwann cells present in the peripheral nervous system. This leads to development of anesthetic skin patch and peripheral nerve thickening^27–29^. The skin lesions thus developed due to nerve damage and loss of sensory perception has gross micro-vascular dysfunction and significant alterations in blood capillary network and these changes are attributed to inflammation^30^. Such altered blood flow may result in altered hydration levels of skin that changes in resident microbial community structure. Such changes have been previously reported by different staining procedures of Slit-skin smear and skin sections of leprosy lesions. Leprosy lesions have acid fast *M. leprae* and a large number of non-acid-fast bacteria which were found to be leprosy derived corynebacteria which was later named as *Corynebacterium tuberculostearicum*^31^. Culturable analysis of skin and ulcers of leprosy have shown presence of opportunistic mycobacteria, *E. coli*, *Klebsiella*, *Proteus mirabilis*, *Proteus spp.*, *Peptostreptococcus, Pseudomonas aeruginosa* and *Staphylococcus aureus*^32,33^. It is now recognized that traditional culture methods have severe limitations in identifying total content of microbial communities. DGGE gel images described in this work provided a definitive evidence for the diverse nature of microbial composition of LS and NLS. Despite having shortfall of DGGE technique, the taxa identified by Sanger Sequencing method showed promising results for presence of unique set of both LS and NLS microbiota. Therefore, 16S rRNA amplicon sequencing with the next-generation sequencing technology was used to profile the skin microbiota of LP demonstrated that LS has much more phylum-level diversity and lesser number of taxa than the HC^10^. The alpha-diversity indices in case of HC from Brazil showed skin microbiome was more diverse than that of LP^19^. Alpha diversity analysis indicated that the skin microbiota of HC from Indian subcontinent possesses significantly lower species richness and diversity.

Nonetheless the outcome from both Indian and Brazilian studies shared many resemblances. Although it is reported that LP have significant number of *M. leprae* and mycobacterial load in dermal layers of their skin^34,35^. While in this study, we have investigated the skin surface and did not find any sequences of *M. leprae.* In rare core taxa, we have noted the sequences representing presence of *Mycobacterium* genus. This was similar in case of skin microbiota of Brazilian LP that also did not show any sequences belonging to members of genus *Mycobacterium.* Skin microbiota from healthy individuals from India and Brazil harbour *Staphylococcus* and *Streptococcus* as major taxa. A known human pathogen *Brevundimonas* which was enriched in Brazilian LP was found to be present also as a major taxon on HC of Indian subjects. LP from Hyderabad and Miraj had significant reduction in taxa belonging to phylum Firmicutes and had augmentation in Proteobacterial taxa which was very similar to Brazilian subjects. This similarity with Brazilian subject was also seen wherein bacteria belonging to genus *Corynebacterium*¸ *Propionibacterium* and *Staphylococcus*, were found to be present on the skin of HC. These genera were found to be marginalized in LS and NLS of LP from both Hyderabad and Miraj. *Limnobacter*, *Paracoccus* and *Psudomonas* were abundant in LS and NLS of Hyderabad subjects while *Kocuria* and *Methylobacterium* were more in case of Miraj subjects. The LS of LP not only differed based on geography but also varied between individual subjects. Many genera were shared by LP from subjects of India and Brazil (*Actinomyces*, *Brevundimonas*, *Corynebacterium*, *Flavobacterium*, *Fusobacterium*, *Haemophilus*, *Kocuria*, *Luteimonas*, *Lysobacter*, *Methylobacterium*, *Morganella*, *Moraxella*, *Prevotella*, *Rothia*, *Sphingomonas*, *Stenotrophomonas*, *Streptomyces*, and *Streptococcus*) while some taxa described in Brazilian subjects like *Bergeyella*, *Chryseobacterium*, *Gemella*, *Lactobacillus* and *Veillonella* were absent in LS of LP from India. Many of these taxa were also found to be present when DGGE gel bands were sequenced and data was analyzed by EZTaxon. Some of the taxa found in LS like *Acinetobacter*^36^, *Bacillus*^37^, *Kocuria*^38^, *Legionella*^39^, *Limnobacter*^40^, and *Paracoccus*^41^ are reported in human skin infections while some taxa like *Gaiella*, *Pseudonocardia* and *Sphinobium* are not reported to be present with human skin or involved in any human infections.

From the perspective of leprosy, the present study is the first from India with detailed information about microbial genera that could lead to dysbiosis. These findings could make it possible to exploit this information in useful ways of therapeutics and diagnostics^42^. Moreover, these observations will also elucidate the existence of the mutual relationship between humans and microbes at the cutaneous-microbiota interface.

In conclusion, we report here proof that the community of bacteria residing on LS of LP and their relative abundances vary from the skin commensals found usually on the skin of a HC. Skin microbiota changes significantly depending upon age, site on skin, environmental settings and skin’s health condition^43–45^ but with several limitations in case of leprosy. The prevalence of reactional leprosy with age and gender as its independent predictors is difficult to conclude because of the lack of statistical significance. Leprosy stigma affects individuals of all ages and gender that makes it difficult to emphasize in terms of core statistics. As per our observations and discussions with the clinicians in leprosy hospitals, differences in the awareness and access to healthcare coverage between male/female and rural/urban population, admission profiles between younger and older generation or the delay in seeking for care because of isolation and rejection from the society also acknowledge the limitations for statistical significance. This study also shows that primarily, there were minor changes observed in the taxonomic abundances of HC when compared to LP emphasizing the spatial variation i.e. population separated geographically. Analysis of beta diversity of both the geographies viz. Hyderabad and Miraj suggest that changes at systemic level probably induces dysbiosis in the skin microbiota. Most probably, leprosy lesions could be the result of a pathogenic cooperation within the microbial consortium. In Indian LP, *Staphylococcus* is the key driver and opportunist that influences the indigenous skin microenvironment. Altogether, we anticipate that these findings will impact the knowledge and new horizons in skin microbiome research areas pertaining to human health and disease. The present study represents only two different locations and population in India. Future research on global level may strengthen the frontiers in skin microbiome studies.

This study adds to the current information on the skin microbiota of HC and LP from an Asian country. This study reiterates/confirms that HC microbiota is similar irrespective of geographical location (Brazil and India; two cities Hyderabad and Miraj) while the skin microbial composition of LS of LP is different for the above geographical sites.

## Methods

### Ethical clearance declaration

The study was approved by the Institutional Ethical Committees of National Centre for Cell Science (NCCS), Pune and respective leprosy research institutes (IEC Approval Ref. No. NCCS/IEC/2016-I/1). We have collected informed consent from study participants for skin swab sample collection as per the institutional ethics committee. It is hereby also declared that all the methods were carried out in accordance with appropriate ethical guidelines and regulations.

### Recruitment of study participants

This study was an observational and non-interventional study, involving HC as well as LP from two distinct leprosy research centres in India, namely Blue Peter Public Health and Research Centre (BPHRC), LEPRA society in Hyderabad, India and Richardson Leprosy Hospital, The Leprosy Mission Trust in Miraj, India. Both the Indian cities; Hyderabad and Miraj are approximately 500 km apart from each other. Details pertaining to the entire study design and methods have been described thoroughly in our previous research article^20^. This article aims at providing the analytical insights into the data generated in the study.

### Pre-processing of 16S rRNA sequence data

Raw paired-end sequence data was subjected to standardized quality processing and has also been described in our research article published previously^20^. Briefly, high quality sequence pairs with a minimum phred33 quality score of 25 were retained using PRINSEQ v0.20.4^46^. Assembly of the high-quality pairs was performed using PEAR v0.9.10^47^ with a minimum overlap threshold of 10%. Only those reads were retained which had a minimum merged length of 400 base pair. Quality was checked and assembled sequence data was then subjected to closed reference OTU classification using RDP classifier v.2.12^48^ at an assignment confidence cut-off of 0.8 as well as *de-novo* classification using VSEARCH v2.8.0^49^.

### Rarefaction analysis

Rarefaction curves were generated for the genus level abundance data using iNEXT v2.0.9^50^ (default parameters were bootstrap replications = 50, knot size = 40 and end point = double the sample size) and ggplot2 v2.1.0.1 R packages^51^. Solid lines of the plot represent interpolation of species accumulation data for each step of sampling unit, while dotted lines represent extrapolation of the said curve. Lines of the plot were coloured to accommodate the metadata pertaining to nature of samples (HS and LP) and the location of sample collection centre in Hyderabad and Miraj.

### Ordination analysis

Jansen-Shannon divergence (JSD) and Partitioning Around Medoids (PAM) based PCoA were adopted as the analysis method as described by Arumugam^52^. Optimum number of clusters were assessed using Calinski-Harabasz (CH) Index^53^. Visualizations for PCoA were generated using R package ade4, cluster and clusterSim, while the JSD distance based ward clustering (dendrogram) was visualized using MicrobiomeAnalyst^54^.

### Diversity analysis

Alpha diversity metrics for richness (Chao-1), species observed (Sobs), evenness (Simpson 1-D) and diversity (Shannon) were calculated for samples from LP affected skin i.e. LS, NLS and HC sites. Taking cues from the observation of ordination analysis that indicated distinct differences in subjects of Hyderabad and Miraj, alpha-diversity analysis of samples from the two locations was carried out independently. Visualizations and statistical tests (Wilcoxon rank sum test for Control vs subjects; Wilcoxon signed rank test for LS vs NLS; Kruskal–Wallis test for LS-NLS-HC population) for the said datasets were performed using R packages ggplot2 and ggpubr^49^ and MicrobiomeAnalyst^52^.

### Top taxonomic units

In order to assess the general community composition for skin microbiota of LP (LS and NLS sites) as compared to HC, abundances of top-10 genera and top-five phyla (in terms of percentage median abundance of taxa in each class of sample) were visualized in Box-plots (inset) and Stacked bar plots generated using ggplot2^49^. Union feature matrix of top taxa for each class of samples was used for this purpose.

### Core taxa analysis

Taxa occurring with at least 0.1% abundance and prevailing consistently in 75% of the samples of a given category were affiliated as core taxa. Rare core taxa were separately assessed using an occurrence threshold of 0.001–0.1% with 75% prevalence threshold.

### Multivariate statistical analyses

Standard statistical tests were performed in order to identify significantly differentiating taxa between HS and LP, as well as various sites of sampling from the LP. Given the paired relationship between LS and NLS samples, Wilcoxon rank sum test was employed for identifying differentiating taxa between samples obtained from HS and LP. Kruskal–Wallis non-parametric test was performed for identifying group level differences across all classes of samples. Bejaminin-Hochberg correction was also performed on all p-values. R package ggpubr v0.1.1^49^ was employed for this purpose. In addition, LefSe^55^ was also used for identifying differentiating taxa between HS and LP.

### Network analysis

Co-occurrence Network analysis was performed based on Rank normalized RDP taxa abundant data. Positive and negative interactions between all the genera in different sample sets were generated using Spearman's correlation coefficient. Network diagrams were generated using Gephi v0.8.2^56^.

### Preliminary DGGE

#### (a) Sample collection

Skin swabs were collected from body sites with a minimum of three bacillary index. Samples were obtained from 3cm^2^ skin area using HiCulture Sterile Skin Swab Collection Device (HiMedia Labs, India) soaked in wetting solution (0.15 M NaCl with 0.1% Tween 20). The samples were stored at 4 °C until further processing.

#### (b) Community DNA extraction

DNA was extracted from the swabs using the MO BIO PowerSoil DNA Isolation Kit with inclusion of freeze–thaw treatment at − 80 °C and 90 °C for 20 min alternatively.

#### (c) PCR amplification and sample preparation

The universal primer pair GC clamped 341F Forward primer and 518R Reverse primer^57^ were used to amplify V3 hyper variable region of 16S rRNA gene. Touch-down PCR was performed using AmpliTaq Gold 360 Mastermix (Thermo Fisher Scientific, USA). The amplicons were subsequently purified using sodium acetate precipitation protocol and the concentration was checked using Nanodrop 1000 spectrophotometer (Thermo Fisher Scientific, USA).

#### (d) Denaturing gradient gel electrophoresis

Polyacrylamide electrophoresis was done using 40% and 60% denaturing concentrations using the DCode Universal Mutation Detection System (Bio-Rad, UK). Denaturing gradient gels were cast with a linear gradient of urea and formamide ranging from 60% at the base to 40% at the top. The gels were left to equilibrate at room temperature in the tank containing 8 L of 1 × Tris buffer solution. 20 µl of sample (10 µl dye and 10 µl PCR product) was loaded in each well of the gel. Gel electrophoresis was carried out at 80 V at constant 60 °C for 18 h. All gels were stained using SYBR Red stain (Life Technologies, USA) for 45 min, after which they were transferred to a UV trans-illuminator (G: BOX Chemi XRQ, Syngene, USA), visualized under UV light (Supplementary Fig. 1). DGGE bands (visualized on a UV trans illuminator), i.e., those that were present across several samples, and unique bands were excised using sterile gel cutting tips (Axygen, Fisher Scientific, USA) and placed in nuclease-free tubes with 10 μl sterile water. The tubes were then incubated at 37 °C for 10–12 h. Before gel extraction, the tubes were vortex for 30 s and then centrifuge for 5 min at 10,000×*g*. PCR products derived from excised DGGE bands were purified using sodium acetate precipitation protocol. Extracts (6 μl) were then used as templates for PCR. PCR products were sequenced using the non-GC clamp (forward) primer. The PCR amplification consist of 6 µl PCR product, 2.5 µl Thermo DYNAzyme Buffer (1X), 0.3 µl Thermo Taq DNA polymerase, 1 µl dNTPs, 1 µl (10 µM) of each forward and reverse primer and 13.20 µl sterile water (50 µl reaction volume). PCR conditions were: 94 °C for 5 min followed by 32 cycles of 94 °C for 30 s, 55 °C for 30 s, and 72 °C for 30 s, ending with a last step of 72 °C for 7 min to ensure complete amplification of the target region.

#### (e) Amplicon sequencing of bacterial 16S rRNA genes

Sequencing was carried out using Sanger sequencing technology. The sequencing reaction volume for each sample was 5 µl and consists of 1 µl PCR product (~ 50 ng/µl), 0.3 µl single sequencing primer 343F, 2 µl Big Dye and 1.7 µl sterile water.

#### (f) 16S sequence data analysis

The sequencing files generated after the sequencing were analyzed using DNASTAR and ChromasPro software. Then we used EzTaxon^58^, a web-based tool for the identification of bacterial species. We analyzed 16S rRNA gene sequences based on sequence similarity approach. We have taken the topmost hit for each sequence that comes in picture using blast algorithms. Altogether a reliable and automated identification of the bacterial species was done using EzBioCloud server.

## Supplementary Information

Supplementary Information.

## Data Availability

Sequencing data for this study may be accesed at NCBI Sequence Read Archive (SRA) as BioProject: PRJNA505133. A detailed data description report may be accessed at https://doi.org/10.1038/s41597-019-0232-1.
