# Supplementary material for: Structural aspects of lesional and non-lesional skin microbiota reveal key community changes in leprosy patients from India
Source: Sci Rep. 2021 Feb 8;11:3294. doi: 10.1038/s41598-020-80533-5 (PMC7870967; doi:10.1038/s41598-020-80533-5)
Supplement: Supplementary file 1 — Supplementary Information. [file 41598_2020_80533_MOESM1_ESM.pdf]

Supplementary Information

Title: Structural aspects of lesional and non-lesional skin microbiota reveal key community changes in leprosy patients from India

Nitin Bayal[1#], Sunil Nagpal[2#], Mohammed Monzoorul Haque[2], Milind S Patole[3], Yogesh Shouche[3], Shekhar C Mande[1,4*], Sharmila S Mande[2*]

[1]National Centre for Cell Science, NCCS Complex, Pune, India;

[2]BioSciences R&D, TCS Research, Tata Consultancy Services, Pune, India;

[3]National Centre for Microbial Resources, NCCS, Pune, India;

[4]Council of Scientific and Industrial Research, Anusandhan Bhawan, 2, Rafi Marg, New Delhi, India

# Equal contribution

* Corresponding authors

Shekhar C. Mande, Director General, Council of Scientific & Industrial Research, Anusandhan Bhawan, 2, Rafi Marg, New Delhi, India;

shekhar@csir.res.in

Sharmila S. Mande, Chief Scientist, Biosciences R&D, TCS Research, Tata Consultancy Services, Pune, India; sharmila.mande@tcs.com

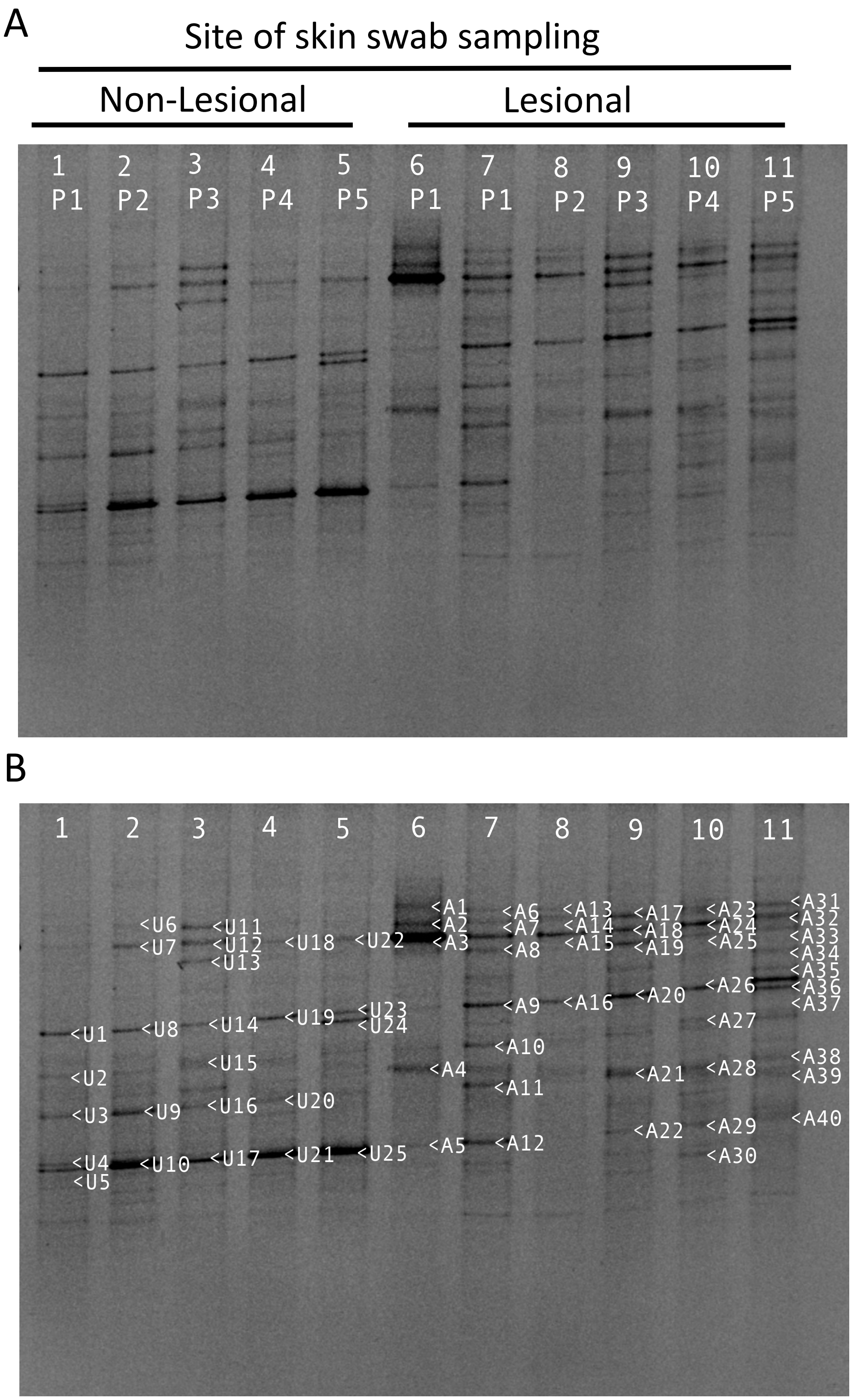

Supplementary Figure 1: DGGE profiles for the V3 hypervariable region of bacterial 16S rRNA gene from lesional and non-lesional skin of leprosy subjects from Hyderabad, India. Numbers in Fig. A indicates the lane number for each sample and Subject ID. Figure B, numbers with initials U and A indicate sample name of DGGE band for sequencing.

Supplementary Figure 2: Principal component analysis (PCoA) of lesional and non-lesional bacterial communities

PCoA clustering of microbial abundance profiles (corresponding to lesional and non-lesional bacterial communities from both geographies) based on Jensen–Shannon (JSD)divergence metric. The aim was to separately analyse the pattern of clustering within lesional and non-lesional samples from Hyderabad or Miraj locations.

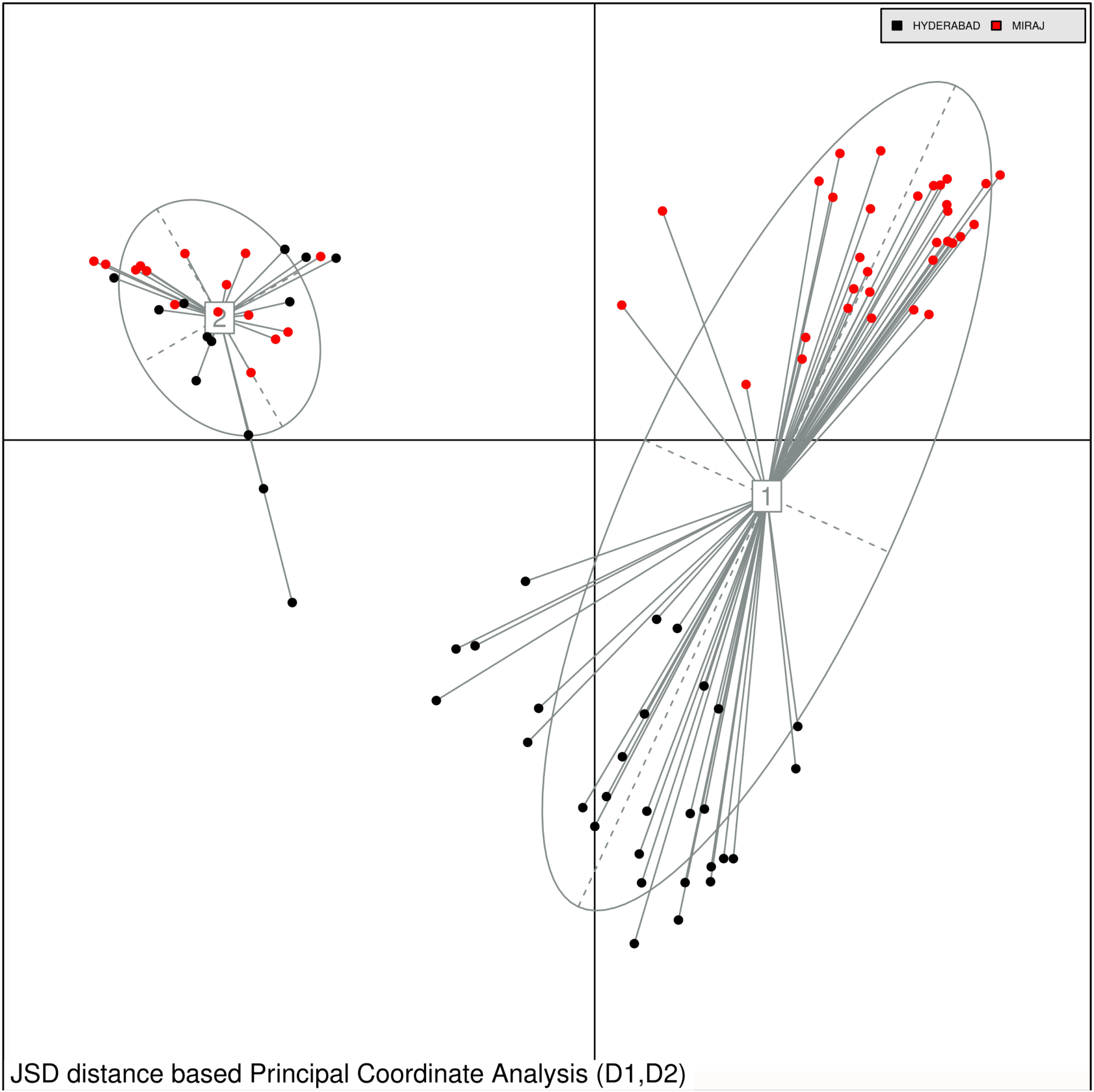

Supplementary Figure 3:Clustering of skin microbiota samples corresponding to healthy controls, lesional and non-lesional skin sites of leprosy subjects

PCoA clustering of microbial abundance profiles corresponding to healthy controls, lesional and non-lesional skin sites of leprosy subjects (from both geographies) based on Jensen–Shannon (JSD)divergence metric.

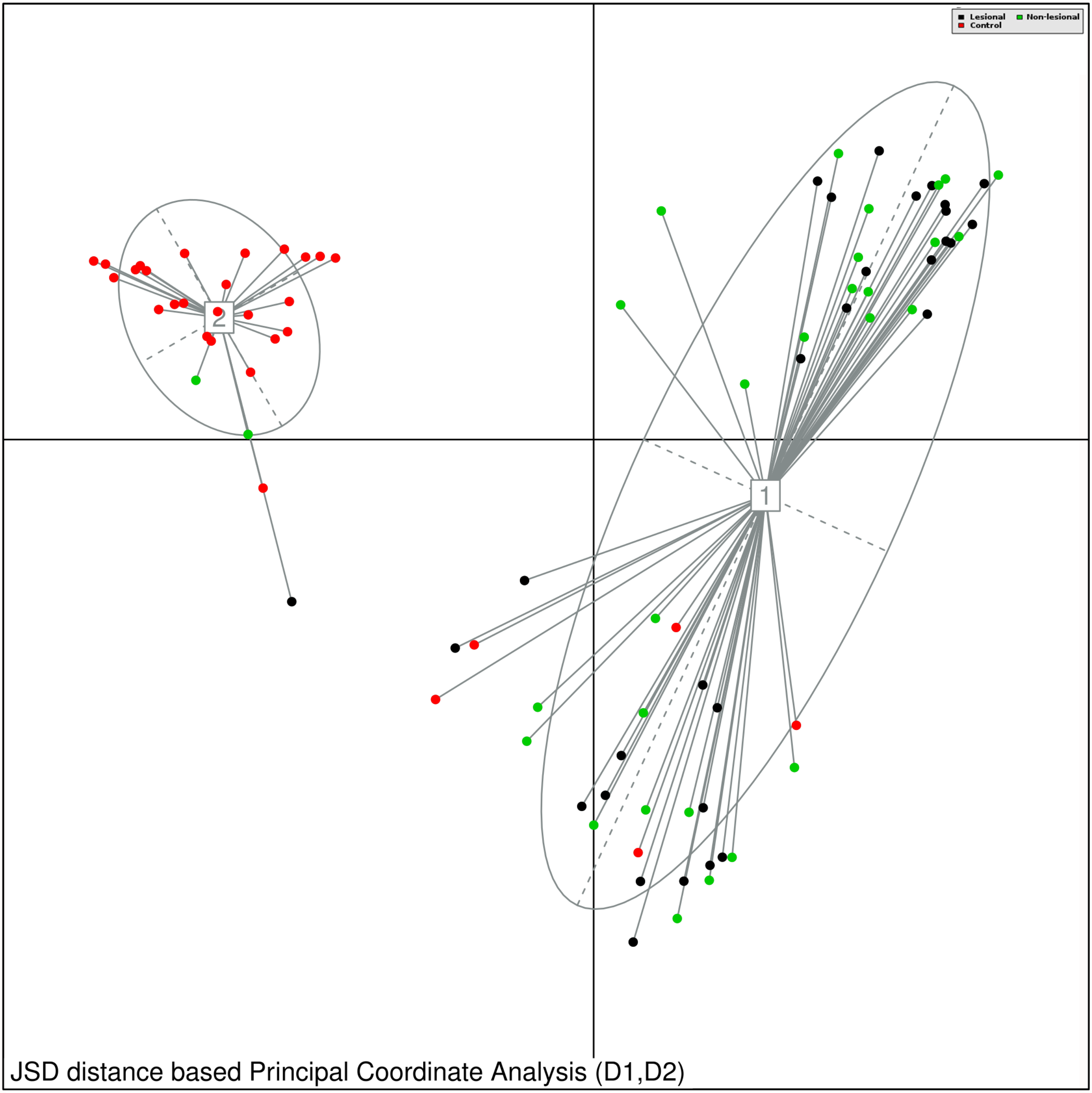

Supplementary Figure 4: Heat map depicting the rare core taxa across various sample types. Z-scores have been employed for depicting the relative distribution.

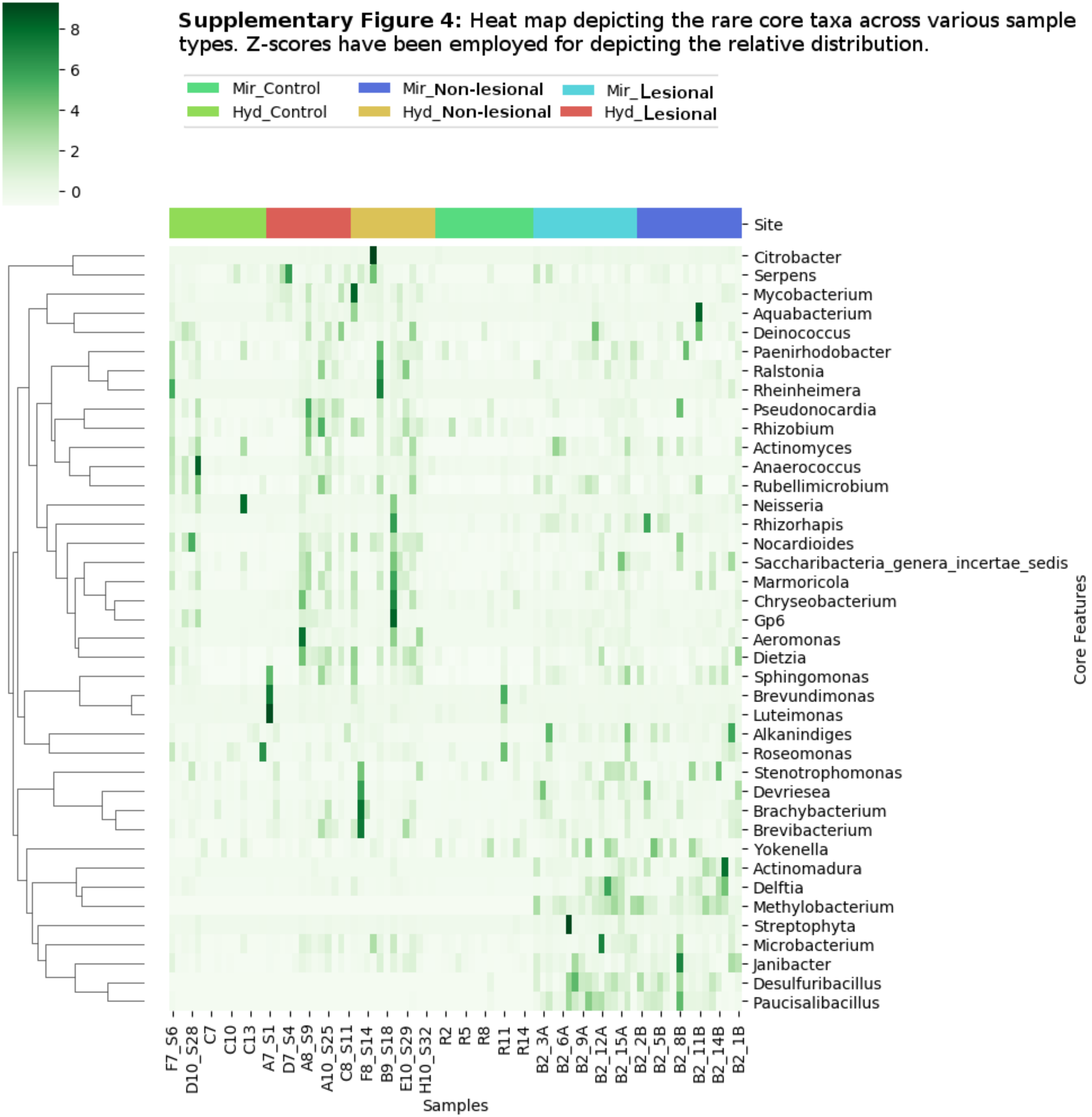

Supplementary Figure 5: Box plots for rare core taxa

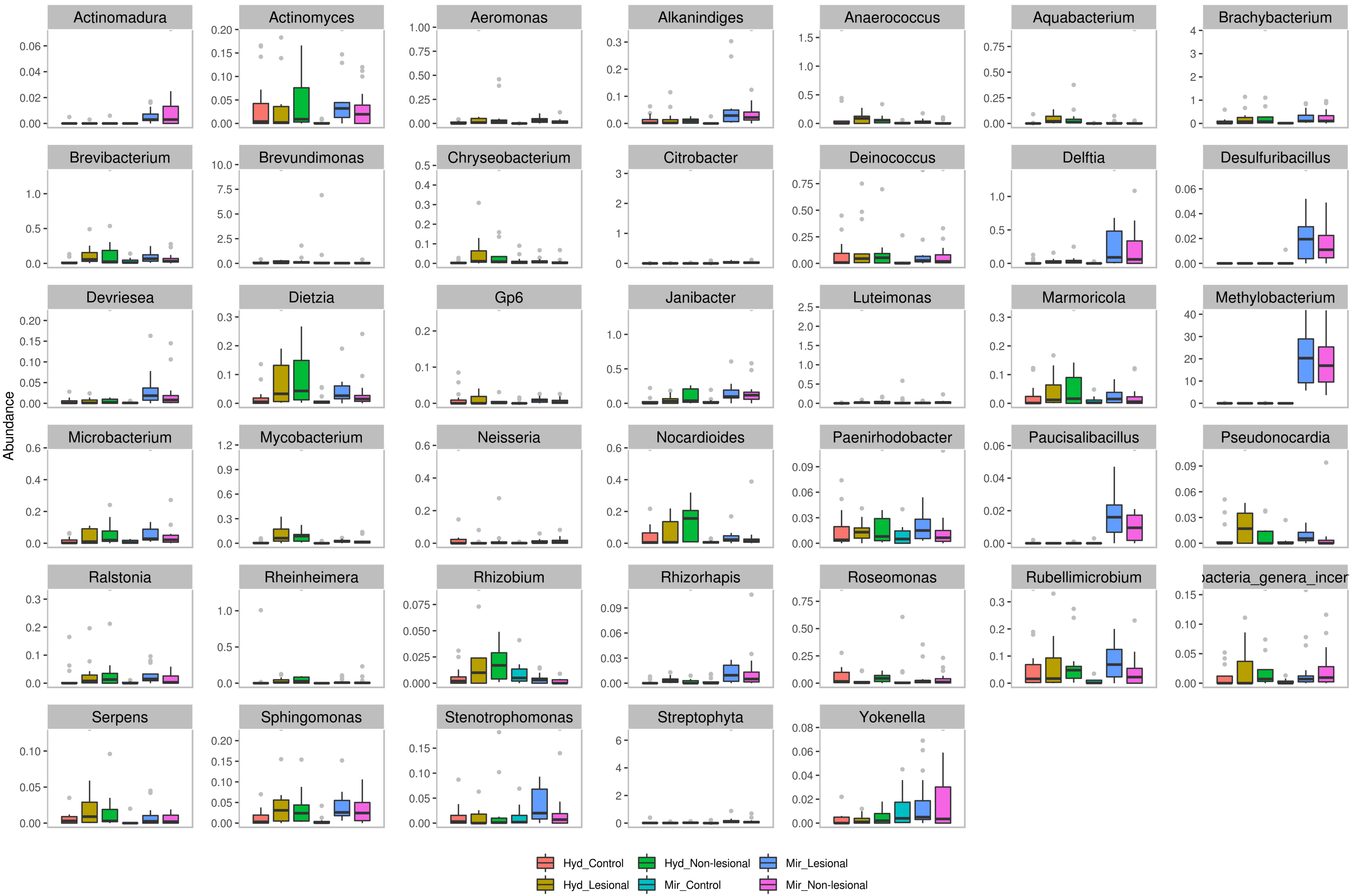

Supplementary Figure 6: Differentiating taxa (identified using LefSe) of lesional and non-lesional skin samples obtained from Hyderabad study participants

Results of LefSe obtained by comparing microbial abundance profiles corresponding to lesional and non-lesional skin of leprosy study participants from Hyderabad.

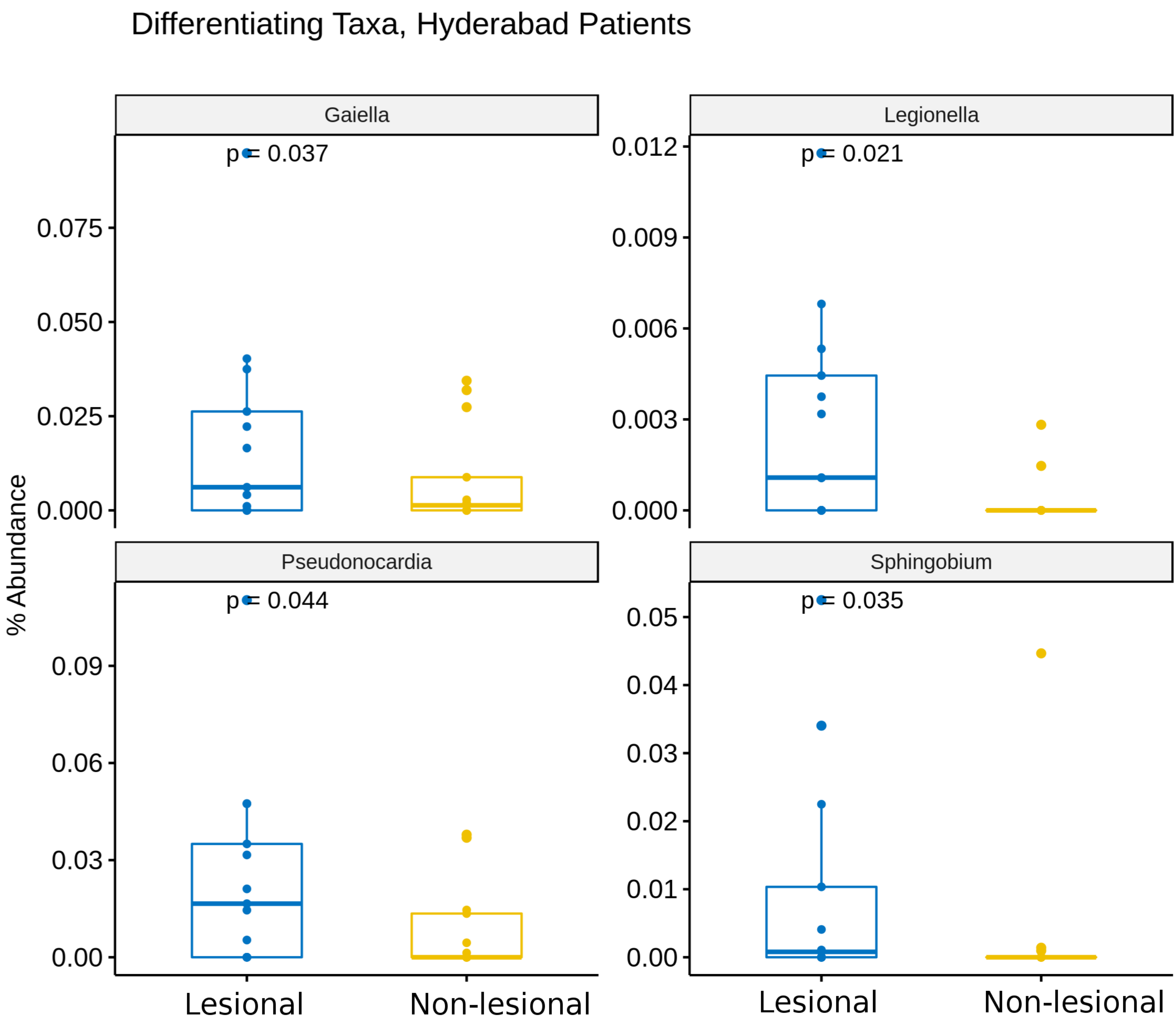

Supplementary Figure 7: Differentiating taxa (identified using LefSe) of lesional and non-lesional skin samples obtained from Miraj study participants

Results of LefSe obtained by comparing microbial abundance profiles corresponding to lesional and non-lesional skin of leprosy study participants from Miraj

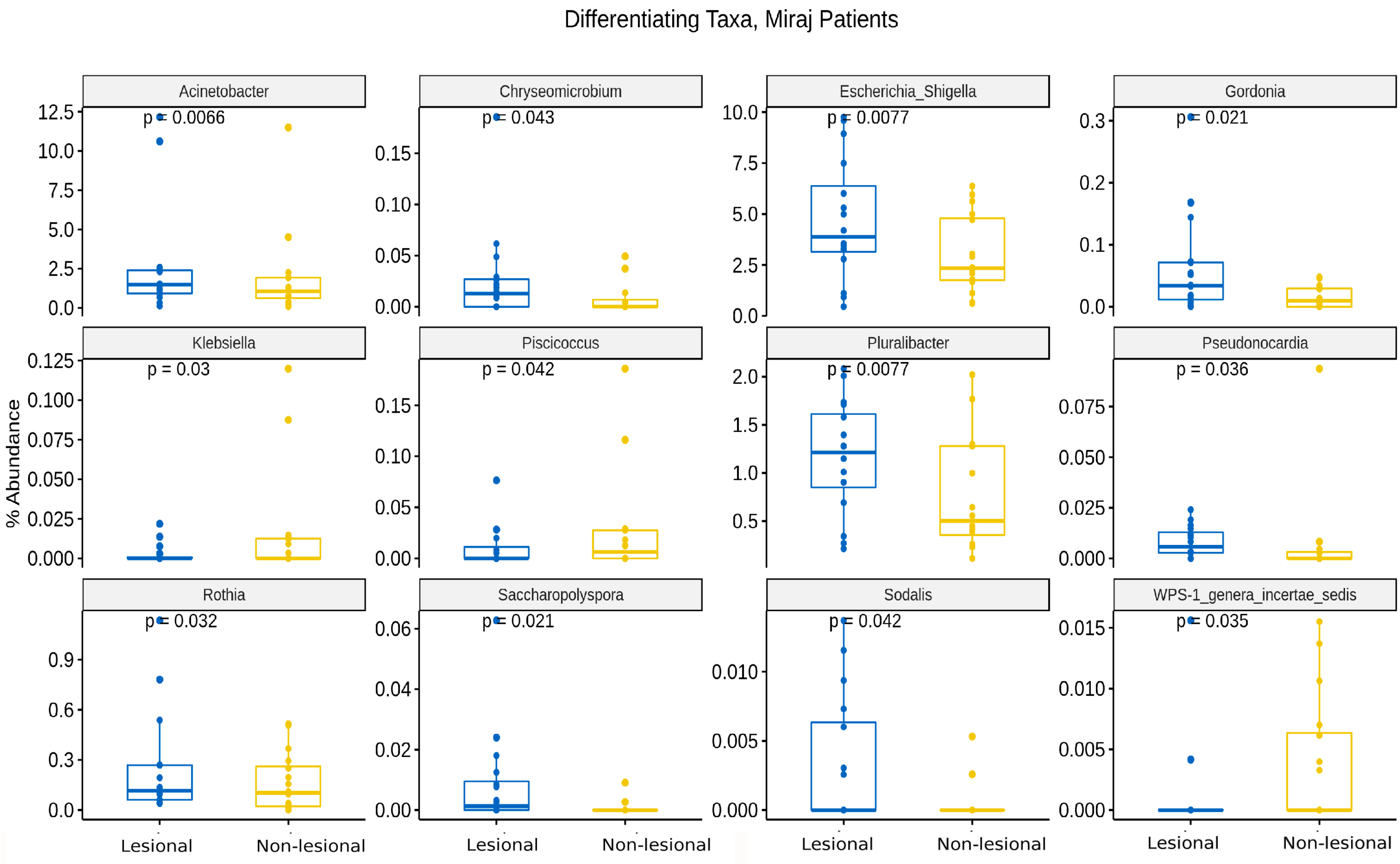

Supplementary Figure 8: Box plots of common differentiating genera across two geographies obtained using iterative approach. Wilcoxon-rank sum test was perfromed between samples from controls and patients (individually in both geographies).

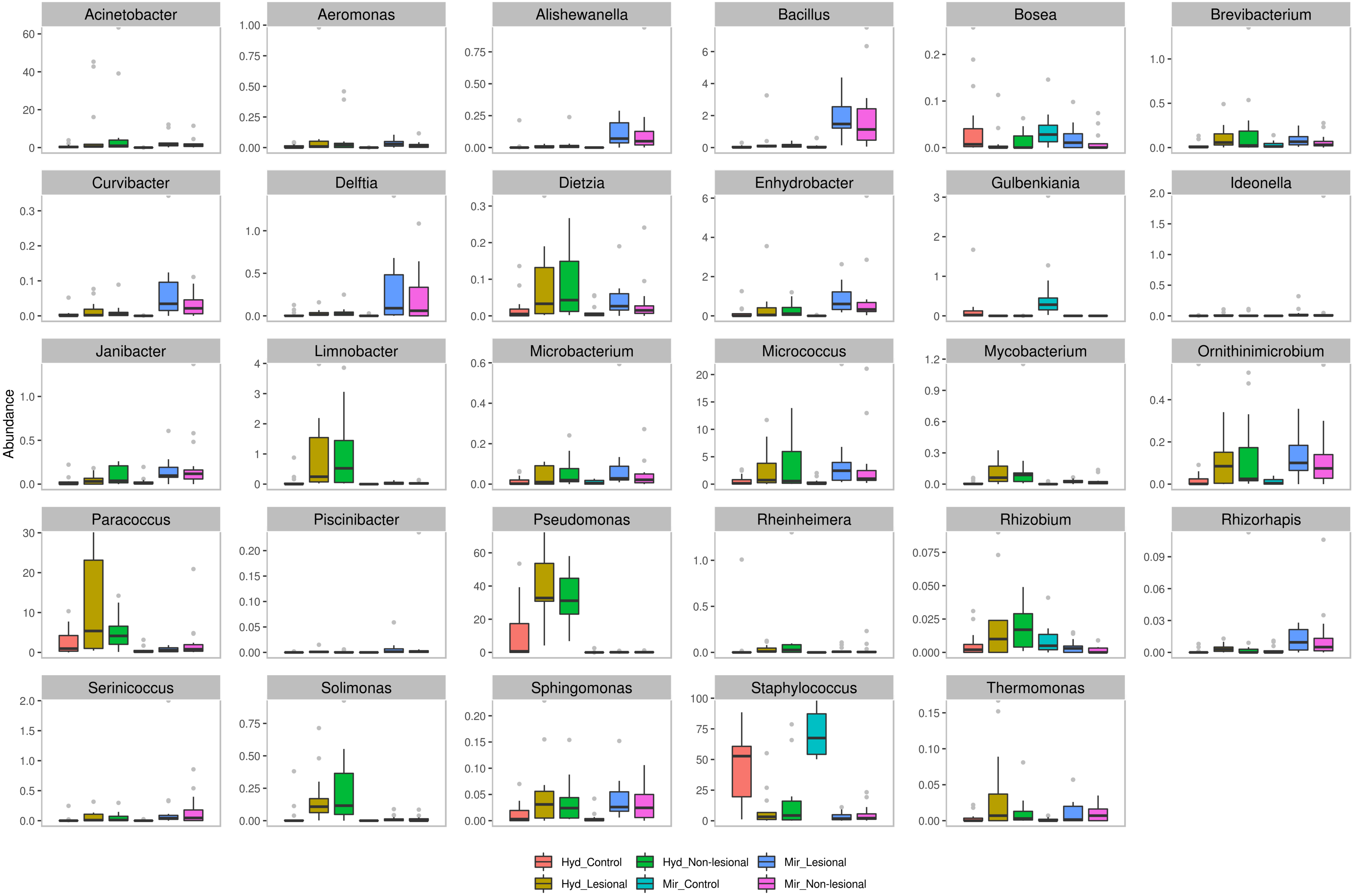

Supplementary Table 1: Good's coverage estimate for sampling completeness for all samples

Samples	Genera	OTU (Filtered)
**B2_16B**	0.97	0.74
**B2_4A**	1.00	0.88
**B2_16A**	1.00	0.92
**B2_5B**	1.00	0.93
**B2_6B**	1.00	0.93
**B2_8A**	1.00	0.94
**B2_6A**	1.00	0.94
**B2_11B**	1.00	0.95
**B2_10A**	1.00	0.95
**B2_5A**	1.00	0.95
**B2_2B**	1.00	0.95
**B2_9A**	1.00	0.96
**B2_4B**	1.00	0.96
**B2_1B**	1.00	0.96
**B2_2A**	1.00	0.96
**B2_11A**	1.00	0.96
**B2_12B**	1.00	0.96
**B2_14A**	1.00	0.97
**B2_10B**	1.00	0.97
**B2_9B**	1.00	0.97
**B2_7A**	1.00	0.97
**B2_7B**	1.00	0.97
**F10_S30**	1.00	0.97
**F9_S22**	1.00	0.97
**B2_15A**	1.00	0.97
**F7_S6**	1.00	0.97
**H8_S16**	1.00	0.97
**B2_12A**	1.00	0.98
**B2_1A**	1.00	0.98
**B2_8B**	1.00	0.98
**A10_S25**	1.00	0.98
**B2_13B**	1.00	0.98
**B2_13A**	1.00	0.98
**B2_14B**	1.00	0.98
**C10_S27**	1.00	0.98
**B2_3A**	1.00	0.98
**B2_3B**	1.00	0.98
**E7_S5**	1.00	0.99
**R11**	1.00	0.99
**A11_S33**	1.00	0.99
**D9_S20**	1.00	0.99
**R13**	1.00	0.99
**F8_S14**	1.00	0.99
**H10_S32**	1.00	0.99
**E8_S13**	1.00	0.99
**A8_S9**	1.00	0.99
**G9_S23**	1.00	0.99
**R9**	1.00	0.99
**H7_S8**	1.00	0.99
**G10_S31**	1.00	0.99
**A7_S1**	1.00	0.99
**H9_S24**	1.00	0.99
**G7_S7**	1.00	0.99

**E10_S29**	1.00	0.99
**R1**	1.00	0.99
**C8**	1.00	0.99
**B7_S2**	1.00	0.99
**R14**	1.00	0.99
**R4**	1.00	0.99
**G8_S15**	1.00	0.99
**C9_S19**	1.00	0.99
**C11**	1.00	0.99
**C12**	1.00	0.99
**C6**	1.00	0.99
**R3**	1.00	0.99
**C8_S11**	1.00	0.99
**D7_S4**	1.00	0.99
**B8_S10**	1.00	0.99
**R2**	1.00	0.99
**C10**	1.00	0.99
**C9**	1.00	0.99
**R10**	1.00	0.99
**R12**	1.00	0.99
**E9_S21**	1.00	1.00
**D10_S28**	1.00	1.00
**D8_S12**	1.00	1.00
**R7**	1.00	1.00
**R5**	1.00	1.00
**C14**	1.00	1.00
**R6**	1.00	1.00
**B9_S18**	1.00	1.00
**C7_S3**	1.00	1.00
**B10_S26**	1.00	1.00
**C15**	1.00	1.00
**C13**	1.00	1.00
**R8**	1.00	1.00
**B2_15B**	1.00	1.00
**C7**	1.00	1.00
**R15**	1.00	1.00

Supplementary Table 2: Distinct DNA bands were eluted from DGGE and were given unique band ID. Each band was subjected to DNA sequencing and the sequence data was used for species origin of the given band by BLAST analysis. The table shows the best hit of BLAST analysis using typed strain data provided on EZTaxon and percent similarity index with BLAST hit.

Band ID	Species / Best hit on BLAST	Strain	Similarity (%)
	**Non-lesional_Skin_Hyderabad**		
U1	*Shigella sonnei*	GTC 781(T)	99.37
U2	No identification		
U3	*Methylobacterium platani*	PMB02(T)	93.51
U4	*Streptomyces lopnurensis*	TRM 49590(T)	94.25
U5	*Corynebacterium imitans*	DSM 44264(T)	92.59
U6	*Asticcacaulis benevestitus*	DSM 16100(T)	87.2
U7	*Staphylococcus hominis subsp. novobiosepticus*	GTC 1228(T)	93.66
U8	*Stenotrophomonas rhizophila*	DSM 14405(T)	48.14
U9	*Paracoccus saliphilus*	YIM 90738(T)	95.93
U10	*Corynebacterium imitans*	DSM 44264(T)	99.22
U11	No identification		
U12	*Staphylococcus hominis subsp. novobiosepticus*	GTC 1228(T)	91.97
U13	*Prolinoborus fasciculus*	CIP 103579(T)	93.24
U14	*Shigella sonnei*	GTC 781(T)	98.75
U15	No identification		
U16	No identification		
U17	*Corynebacterium imitans*	DSM 44264(T)	100
U18	*Staphylococcus hominis subsp. novobiosepticus*	GTC 1228(T)	94
U19	*Shigella sonnei*	GTC 781(T)	98.75
U20	*Paracoccus kocurii*	JCM 7684(T)	93.14
U21	*Corynebacterium imitans*	DSM 44264(T)	100
U22	*Staphylococcus hominis subsp. novobiosepticus*	GTC 1228(T)	90.07
U23	*Shigella sonnei*	GTC 781(T)	98.74
U24	*Acinetobacter indicus*	CIP 110367(T)	98.66
U25	*Corynebacterium imitans*	DSM 44264(T)	99.25
	**Lesional_Skin_Hyderabad**		
A1	*Staphylococcus hominis subsp. novobiosepticus*	GTC 1228(T)	99.32
A2	*Staphylococcus hominis subsp. novobiosepticus*	GTC 1228(T)	99.32
**Band ID**	**Species / Best hit on BLAST**	**Strain**	**Similarity (%)**
A3	*Staphylococcus hominis subsp. novobiosepticus*	GTC 1228(T)	98.64
A4	*Staphylococcus hominis subsp. novobiosepticus*	GTC 1228(T)	98.65
A5	*Corynebacterium ureicelerivorans*	IMMIB RIV-2301(T)	97.79
A6	*Staphylococcus schweitzeri*	FSA084(T)	100
A7	*Staphylococcus hominis subsp. novobiosepticus*	GTC 1228(T)	95.04
A8	*Shigella sonnei*	GTC 781(T)	95.71
A9	No identification		
A10	*Paracoccus kocurii*	JCM 7684(T)	95.2
A11	*Corynebacterium ureicelerivorans*	IMMIB RIV-2301(T)	97.53
A12	*Kocuria flava*	HO-9041(T)	96.38
A13	*Staphylococcus cohnii subsp. cohnii*	ATCC 29974(T)	99.34
A14	*Agromyces rhizospherae*	IFO 16236(T)	87.02
A15	*Neokomagataea thailandica*	AH11(T)	87.9
A16	*Shigella sonnei*	GTC 781(T)	94.51
A17	*Enhydrobacter aerosaccus*	LMG 21877(T)	87.84
A18	*Canibacter oris*	IMMIB Q2029717(T)	86.73
A19	*Prolinoborus fasciculus*	CIP 103579(T)	92.41
A20	*Shigella sonnei*	GTC 781(T)	98.69
A21	*Paracoccus kocurii*	JCM 7684(T)	86.99
A22	*Janibacter cremeus*	HR08-44(T)	96.9
A23	*Burkholderia cepacia*	ATCC 25416(T)	98.64
A24	*Pseudomonas abietaniphila*	ATCC 700689(T)	89.33
A25	*Anaeromusa acidaminophila*	DSM 3853(T)	94.55
A26	*Shigella sonnei*	GTC 781(T)	100
A27	*Enterobacter xiangfangensis*	10-17(T)	93.42
A28	*Serinicoccus profundi*	MCCC 1A05965(T)	96.21
A29	*Ornithinimicrobium pekingense*	DSM 21552(T)	99.22
A30	*Saccharothrix yanglingensis*	Hhs.015(T)	89.39
A31	*Moraxella osloensis*	AerLab-37	87.07
A32	*Staphylococcus hominis subsp. novobiosepticus*	GTC 1228(T)	89.26
**Band ID**	**Species / Best hit on BLAST**	**Strain**	**Similarity (%)**
A33	*Burkholderia territorii*	LMG 28158(T)	91.1
A34	No identification		
A35	*Shigella sonnei*	GTC 781(T)	98.73
A36	*Acinetobacter indicus*	CIP 110367(T)	96.62
A37	No identification		
A38	*Pleomorphomonas diazotrophica*	R5-392(T)	91.04
A39	*Corynebacterium jeikeium*	ATCC 43734(T)	91.79
A40	*Propionibacterium acnes*	DSM 1897(T)	89.93
	**Non-lesional_Skin_Miraj**		
U26	*Mycobacterium flavescens*	ATCC 14474(T)	77.36
U27	*Pseudactinotalea terrae*	5GHs33-3(T)	91.95
U28	*Corynebacterium ulceribovis*	DSM 45146(T)	82.8
U29	*Methylobacterium cerastii*	C44(T)	86.54
U30	*Pseudactinotalea terrae*	5GHs33-3(T)	94.04
U31	*Methylobacterium gossipiicola*	Gh-105(T)	90.85
U32	*Paracoccus aestuariivivens*	GHD-30(T)	90.6
U33	*Paracoccus siganidrum*	M26(T)	88.67
U34	*Microvirga soli*	R491(T)	89.4
U35	*Bacillus gaemokensis*	KCTC 13318(T)	81.87
U36	*Staphylococcus hominis subsp. hominis*	DSM 20328(T)	92.94
U37	*Staphylococcus argensis*	M4S-6(T)	84.52
U38	*Bacillus megaterium*	NBRC 15308(T)	89.94
U39	*Methylobacterium gossipiicola*	Gh-105(T)	91.13
U40	*Methylobacterium gossipiicola*	Gh-105(T)	90.13
U41	*Methylobacterium gossipiicola*	Gh-105(T)	91.5
U42	*Corynebacterium lipophiloflavum*	DSM 44291(T)	83.97
U43	*Corynebacterium lipophiloflavum*	DSM 44291(T)	90.85
U44	*Methylobacterium gossipiicola*	Gh-105(T)	93.96
U45	*Corynebacterium appendicis*	DSM 44531(T)	94.7
**Band ID**	**Species / Best hit on BLAST**	**Strain**	**Similarity (%)**
U46	*Methylobacterium gossipiicola*	Gh-105(T)	93.29
U47	No identification		
U48	*Actinokineospora acnipugnans*	R434(T)	79.88
U49	*Microvirga lupini*	Lut6(T)	75.76
U50	*Mycoplasma feriruminatoris*	G5847(T)	68.48
U51	No identification		
U52	*Staphylococcus hominis subsp. hominis*	DSM 20328(T)	90.59
U53	*Staphylococcus hominis subsp. hominis*	DSM 20328(T)	89.6
U54	*Staphylococcus hominis subsp. hominis*	DSM 20328(T)	95.81
U55	*Staphylococcus hominis subsp. hominis*	DSM 20328(T)	94.64
U56	*Bacillus halmapalus*	DSM 8723(T)	85.8
U57	*Brevundimonas halotolerans*	MCS 24(T)	91.33
U58	*Prauserella soli*	12-833(T)	81.25
U59	No identification		
U60	*Corynebacterium tuberculostearicum*	CIP107291(T)	94.12
U61	*Corynebacterium timonense*	5401744(T)	86.84
U62	*Corynebacterium afermentans*	DSM 44280(T)	88.31
U63	*Microvirga soli*	R491(T)	89.47
U64	*Methylobacterium gossipiicola*	Gh-105(T)	92
U65	*Falsirhodobacter deserti*	W402(T)	87.74
U66	*Corynebacterium mycetoides*	DSM 20632(T)	86.18
U67	*Corynebacterium fournierii*	Marseille-P2948(T)	90
U68	*Corynebacterium pilbarense*	IMMIB WACC 658(T)	92.31
U69	*Staphylococcus equorum subsp. linens*	RP29(T)	88.76
U70	*Corynebacterium lactis*	RW2-5(T)	86.36
U71	*Microvirga soli*	R491(T)	82.78
U72	*Methylobacterium gossipiicola*	Gh-105(T)	91.22
U73	*Paracoccus niistensis*	NII-0918(T)	88.44
U74	*AB010906_s*	QKT1393-078Joya	91.39
	**Lesional_Skin_Miraj**		
**Band ID**	**Species / Best hit on BLAST**	**Strain**	**Similarity (%)**
A41	*Methylobacterium gossipiicola*	Gh-105(T)	82.17
A42	*Methylobacterium gossipiicola*	Gh-105(T)	87.74
A43	*Methylobacterium gossipiicola*	Gh-105(T)	87.58
A44	*Methyloferula stellata*	AR4(T)	80
A45	No identification		
A46	No identification		
A47	No identification		
A48	No identification		
A49	*Methylobacterium gossipiicola*	Gh-105(T)	83.04
A50	*Methylobacterium gossipiicola*	Gh-105(T)	88.31
A51	*Methylobacterium gossipiicola*	Gh-105(T)	89.47
A52	*Ruminococcus albus*	7(T)	73.61
A53	*Methylobacterium gossipiicola*	Gh-105(T)	86.27
A54	*Methylobacterium bullatum*	F3.2(T)	83.55
A55	*Methylobacterium trifolii*	TA73(T)	85.71
A56	*Methylobacterium gossipiicola*	Gh-105(T)	91.89
A57	*Microvirga lupini*	Lut6(T)	84.08
A58	*Methylobacterium gossipiicola*	Gh-105(T)	90.07
A59	*Bosea vaviloviae*	Vaf-18(T)	84.87
A60	*Falsirhodobacter deserti*	W402(T)	87.1
A61	No identification		
A62	*Pseudactinotalea terrae*	5GHs33-3(T)	90.38
A63	*Methylobacterium gossipiicola*	Gh-105(T)	91.77
A64	No identification		
A65	*Methylobacterium gossipiicola*	Gh-105(T)	80.81
A66	*Methylobacterium gossipiicola*	Gh-105(T)	82.56
A67	No identification		
A68	No identification		
A69	*Methylobacterium gossipiicola*	Gh-105(T)	79.39
A70	No identification		
A71	*Methylobacterium gossipiicola*	Gh-105(T)	86.31
A72	*Methylobacterium gossipiicola*	Gh-105(T)	94.08
A73	No identification		
A74	*Microvirga lupini*	Lut6(T)	85.03
A75	*Methylobacterium gossipiicola*	Gh-105(T)	94.56
A76	*Methylocella palustris*	K(T)	94.59
A77	*Methylocella palustris*	K(T)	94.59
A78	*Prauserella soli*	12-833(T)	91.39
A79	*Corynebacterium appendicis*	DSM 44531(T)	92.11
A80	*Corynebacterium lipophiloflavum*	DSM 44291(T)	91.5
A81	No identification		
A82	*Saccharomonospora azurea*	NA-128(T)	90.57
A83	*Methylobacterium gossipiicola*	Gh-105(T)	90.73
A84	*Corynebacterium appendicis*	DSM 44531(T)	95.97
A85	*Saccharomonospora halophila*	8(T)	77.12
A86	*Bacillus altitudinis*	41KF2b(T)	91.62
A87	*Staphylococcus epidermidis*	ATCC 14990(T)	90.12
A88	*Microvirga soli*	R491(T)	77.33
A89	*Acinetobacter proteolyticus*	NIPH 809(T)	92.9
A90	No identification		
A91	*Novosphingobium naphthae*	D39(T)	66.26
A92	*Micrococcus aloeverae*	AE-6(T)	93.33
A93	*Methylobacterium gossipiicola*	Gh-105(T)	93.24
A94	*Paracoccus marcusii*	DSM 11574(T)	90.6
A95	*Microvirga soli*	R491(T)	89.4
A96	*Corynebacterium appendicis*	DSM 44531(T)	93.29
A97	*Staphylococcus hominis subsp. hominis*	DSM 20328(T)	88.82
A98	*Methylobacterium gossipiicola*	Gh-105(T)	82.25
A99	*Methylobacterium gossipiicola*	Gh-105(T)	93.24
A100	*Corynebacterium afermentans subsp. afermentans*	DSM 44280(T)	91.36
A101	*Methylobacterium gossipiicola*	Gh-105(T)	95.21
A102	*Methylobacterium gossipiicola*	Gh-105(T)	86.18
A103	*Microvirga soli*	R491(T)	82.69
A104	*Microvirga soli*	R491(T)	82.76
A105	*Staphylococcus argensis*	M4S-6(T)	78.7
A106	*Microvirga soli*	R491(T)	83.14
A107	*Methylobacterium bullatum*	F3.2(T)	84.34
A108	*Methylobacterium gossipiicola*	Gh-105(T)	84.66
A109	*Chelatococcus asaccharovorans*	TE2(T)	69.64
A110	No identification		
A111	No identification		
A112	*Methylobacterium gossipiicola*	Gh-105(T)	90.67
A113	*Corynebacterium riegelii*	DMMZ 2415(T)	81.46
A114	*Methylobacterium isbiliense*	AR24(T)	76.54
A115	*Methylobacterium gossipiicola*	Gh-105(T)	93.38
A116	*Falsirhodobacter deserti*	W402(T)	88.89
A117	No identification		
A118	No identification		
A119	*Prauserella oleivorans*	RIPI(T)	80.52
A120	No identification		
A121	LT821240_s	Marseille-P2417	86.09
A122	No identification		

Genus Level Abundance Data

Supplementary File 1: A consolidated matrix comprising genus-level taxonomic abundance data corresponding to all samples analysed in the present study.

Downloadable from following link. PDF view on next page:

https://springernature.figshare.com/articles/Leprosy_Microbiome_Genus_Level_Abundance_Data/8020394/1

Genera	A10_S25	A11_S33	A7_S1	A8_S9	B10_S26	B2_10A	B2_10B	B2_11A	B2_11B	B2_12A	B2_12B	B2_13A	B2_13B	B2_14A	B2_14B	B2_15A	B2_15B	B2_16A
Brachybacteri	1107	192	38	324	0	27	43	33	23	123	6	27	23	6	0	39	206	323
Devriesea	14	12	1	8	0	1	3	12	2	19	1	3	3	0	0	17	2	12
Dermabacter	19	25	0	5	0	3	14	0	8	0	1	0	0	0	28	0	0	28
Helcobacillus	0	0	0	0	0	0	0	7	0	0	0	0	0	0	0	0	0	0
Kocuria	5788	3073	70	1430	3	7198	12925	3014	1535	2039	1614	6228	4042	1143	5940	3558	5962	3314
Micrococcus	8385	2735	3180	909	406	118	110	265	156	892	117	261	210	82	61	790	804	1514
Rothia	84	25	11	86	26	34	43	43	21	275	60	67	71	15	42	197	239	36
Citricoccus	8	4	0	16	50	0	0	2	0	0	0	0	0	0	0	0	0	5
Arthrobacter	28	87	5	102	1	6	2	3	7	0	0	0	1	1	0	19	93	5
Nesterenkonia	6	11	23	0	0	9	24	0	0	0	0	0	0	0	15	37	0	0
Garicola	1	6	0	0	0	0	0	0	0	0	0	0	0	0	0	0	0	5
Enteractinoco	0	40	0	0	0	0	0	0	2	0	0	0	0	0	0	14	0	0
Yaniella	0	0	0	0	0	0	1	0	0	0	0	0	0	0	0	0	0	0
Tersicoccus	0	0	0	0	0	0	0	0	0	0	0	2	0	0	0	0	0	0
Sinomonas	0	0	0	0	0	0	0	0	0	0	0	0	0	0	0	9	0	0
Zhihengliuella	0	0	0	0	0	0	0	0	0	0	0	0	0	0	0	0	0	0
Renibacterium	0	0	0	0	0	0	0	0	0	0	0	0	0	0	0	0	0	0
Dermacoccus	76	0	8	0	0	3	0	0	2	0	0	0	0	0	0	15	0	0
Kytococcus	125	41	14	8	0	1	0	17	41	163	0	0	0	10	0	53	360	187
Barrientosiimo	59	0	0	0	0	5	0	0	0	0	7	0	0	0	0	0	0	1
Yimella	1	0	0	0	0	0	0	0	3	0	0	0	0	0	1	0	0	0
Calidifontibact	0	0	0	3	0	0	0	0	0	0	0	0	0	0	0	0	0	0
Flexivirga	0	0	0	0	0	1	0	0	0	0	0	0	0	0	0	0	0	0
Luteipulveratu	0	0	0	0	0	0	0	0	0	0	0	0	0	0	0	0	0	0
Microbacteriu	107	66	5	85	0	6	3	9	4	144	6	11	0	12	16	33	0	39
Zimmermanne	16	15	0	12	0	1	0	0	0	0	0	0	0	0	2	8	0	46
Agrococcus	21	167	0	35	0	4	10	37	8	3	0	10	0	0	0	0	0	8
Leucobacter	1	11	0	0	0	0	0	0	2	0	0	0	6	1	0	0	0	0
Agromyces	0	21	0	2	0	0	0	3	0	0	0	0	0	1	0	0	0	1
Naasia	0	12	0	0	0	0	0	6	3	0	0	0	0	0	0	0	0	0
Curtobacteriu	0	20	0	6	0	3	0	0	0	0	0	0	0	0	0	13	0	0
Amnibacterium	0	9	0	0	0	0	0	0	0	0	0	0	0	0	0	0	0	0
Rhodoluna	0	0	0	35	0	0	0	0	1	0	0	0	0	0	0	0	0	0
Alpinimonas	0	0	0	57	0	0	0	0	0	0	0	0	0	0	0	0	0	0
Gryllotalpicola	0	0	0	10	0	0	0	0	0	0	0	0	0	0	0	0	0	0
Leifsonia	0	0	0	0	72	0	0	0	1	0	0	0	0	0	0	0	60	0
Microcella	0	0	0	0	0	0	0	0	0	0	0	0	0	0	0	38	0	0
Diaminobutyri	0	0	0	0	0	0	0	0	0	0	0	0	0	0	0	0	0	0
Gulosibacter	0	0	0	0	0	0	0	0	0	0	0	0	0	0	0	0	0	0
Lysinimonas	0	0	0	0	0	0	0	0	0	0	0	0	0	0	0	0	0	0
Homoserinibac	0	0	0	0	0	0	0	0	0	0	0	0	0	0	0	0	0	0
Frigoribacteri	0	0	0	0	0	0	0	0	0	0	0	0	0	0	0	0	0	0
Herbiconiux	0	0	0	0	0	0	0	0	0	0	0	0	0	0	0	0	0	0
Serinicoccus	305	23	0	118	0	26	2	27	10	79	65	7	0	7	35	12	694	8
Janibacter	177	86	17	90	0	23	47	35	19	21	24	0	14	11	2	7	0	98
Ornithinimicro	190	68	8	113	106	43	39	126	28	27	3	0	14	7	6	124	459	35
Ornithinibacter	26	0	0	391	0	0	0	0	0	0	1	0	0	0	0	0	0	0
Knoellia	4	1	0	18	0	12	31	0	0	0	2	0	0	0	0	13	0	8
Tetrasphaera	7	1	0	24	1	3	0	0	0	1	0	0	0	0	0	0	3	2
Phycicoccus	1	3	0	0	0	0	0	1	0	0	0	0	0	0	0	0	0	1
Arsenicicoccu	0	12	0	0	0	0	0	0	0	0	0	0	0	0	0	0	0	0
Terracoccus	0	1	0	0	0	0	0	0	0	0	0	0	0	0	0	0	0	0
Intrasporangi	0	3	0	0	0	0	0	0	1	0	0	0	0	0	0	0	0	0
Kribbia	0	0	0	32	0	0	0	0	0	0	0	0	0	0	0	0	0	0
Ornithinicoccu	0	0	0	32	0	0	0	0	3	83	1	0	0	0	0	0	0	6
Aquipuribacter	0	0	0	0	0	0	0	9	0	0	0	0	0	0	0	0	0	0
Terrabacter	0	0	0	0	0	0	0	0	0	0	0	0	0	0	0	0	102	0

Genera	B2_16B	B2_1A	B2_1B	B2_2A	B2_2B	B2_3A	B2_3B	B2_4A	B2_4B	B2_5A	B2_5B	B2_6A	B2_6B	B2_7A	B2_7B	B2_8A	B2_8B	B2_9A	B2_9B	B7_S2	B8_S10	B9_S18	C10
Brachybacteri	5	31	309	88	9	298	372	163	83	21	42	8	27	9	11	47	196	47	23	622	87	27	9
Devriesea	0	4	53	15	2	56	56	6	1	6	9	0	6	1	1	6	7	6	0	22	9	2	2
Dermabacter	0	23	12	10	0	0	0	14	0	43	89	0	9	4	1	14	0	5	9	0	0	2	0
Helcobacillus	0	0	0	0	0	0	0	0	0	0	0	0	0	0	0	0	0	0	0	0	0	0	0
Kocuria	44	3897	3952	2474	776	4943	976	672	1010	1875	2085	1530	1172	1839	491	5405	3056	1986	903	33	151	120	12
Micrococcus	21	1458	10580	1219	189	198	388	1637	283	189	263	828	956	1397	1132	304	393	6749	3824	10943	507	183	176
Rothia	0	84	259	18	3	21	4	10	13	90	94	235	185	15	4	45	0	19	12	0	69	5	0
Citricoccus	0	0	0	0	2	0	1	5	0	0	0	0	0	2	0	0	0	0	0	0	8	0	0
Arthrobacter	0	4	11	14	0	0	7	12	1	1	4	3	1	2	0	0	0	0	0	16	5	5	0
Nesterenkonia	0	0	0	0	0	0	0	4	0	3	0	0	0	0	0	0	0	0	2	0	0	0	0
Garicola	0	0	0	0	0	0	0	0	0	0	0	0	0	0	0	0	0	0	0	0	7	0	0
Enteractinoco	0	0	0	0	0	0	0	2	0	0	0	0	0	0	0	2	0	0	0	0	0	29	0
Yaniella	0	0	0	0	0	0	0	0	0	0	0	0	1	0	0	0	0	0	0	0	0	0	0
Tersicoccus	0	0	0	0	0	0	0	0	0	0	0	0	0	0	0	0	0	0	0	0	0	0	0
Sinomonas	0	0	0	0	0	0	0	0	0	0	0	0	0	0	0	0	0	0	0	0	0	0	0
Zhihengliuella	0	0	0	0	0	0	0	0	0	0	0	0	0	0	0	0	0	0	0	0	0	0	0
Renibacterium	0	0	0	0	0	0	0	0	0	0	0	0	0	0	0	0	0	0	0	0	0	0	0
Dermacoccus	0	0	0	0	0	11	13	0	0	41	51	67	24	0	4	103	0	0	0	0	0	0	0
Kytococcus	9	0	30	77	11	4	3	142	36	42	75	24	34	8	12	34	0	87	24	63	12	31	15
Barrientosiimo	0	82	177	28	0	17	72	0	0	0	45	20	7	7	16	18	0	0	0	0	19	0	0
Yimella	0	0	0	0	0	2	0	0	0	0	1	4	4	1	0	1	0	0	0	0	0	0	0
Calidifontibact	0	0	3	0	0	2	16	0	0	0	1	8	0	0	0	0	0	0	2	0	0	0	0
Flexivirga	0	0	0	0	0	0	0	0	0	0	0	0	0	0	0	0	0	0	0	0	0	0	0
Luteipulveratu	0	0	0	0	0	0	0	0	0	0	0	0	0	0	0	0	0	0	0	0	0	0	0
Microbacteriu	1	42	11	7	3	8	0	8	23	7	8	4	18	4	5	3	61	12	1	9	49	8	0
Zimmermanne	0	0	3	0	0	3	0	24	9	3	4	2	4	4	0	1	0	9	2	0	0	0	0
Agrococcus	0	0	0	10	0	7	16	9	5	9	4	0	10	3	0	0	0	0	0	3	0	0	0
Leucobacter	0	0	4	0	0	0	1	0	0	6	0	0	0	0	0	0	0	0	0	0	0	0	0
Agromyces	0	0	0	0	0	0	0	4	2	0	0	0	0	0	0	0	0	0	0	0	0	0	0
Naasia	0	0	0	0	0	0	0	0	0	0	0	0	0	0	0	0	0	0	0	0	0	0	0
Curtobacteriu	0	0	2	0	0	0	4	5	3	0	4	0	0	0	0	0	0	0	0	1	9	0	0
Amnibacterium	0	0	0	0	0	0	0	0	0	0	0	0	0	0	0	0	0	0	0	0	0	0	0
Rhodoluna	0	0	0	0	0	0	0	1	0	0	0	0	0	0	0	0	0	0	0	0	117	1	0
Alpinimonas	0	0	0	0	0	0	0	0	0	0	0	0	0	0	0	0	0	0	0	0	6	1	0
Gryllotalpicola	0	0	0	0	0	0	0	0	0	0	0	0	0	0	0	0	0	0	0	0	0	0	0
Leifsonia	0	0	0	0	0	1	0	0	0	0	0	0	0	0	0	0	0	0	0	0	0	0	0
Microcella	0	0	0	0	0	0	0	0	0	0	0	0	0	0	0	0	0	0	2	0	0	0	0
Diaminobutyri	0	0	3	0	0	0	4	1	0	0	0	0	1	0	0	0	0	0	0	0	3	0	0
Gulosibacter	0	0	0	0	0	1	0	0	0	0	0	0	0	6	0	0	0	0	0	0	29	3	0
Lysinimonas	0	0	0	0	0	0	0	0	0	0	0	0	0	0	0	0	0	0	0	0	0	0	0
Homoserinibac	0	0	0	0	0	0	0	0	0	0	0	0	0	0	0	0	0	0	0	0	0	0	0
Frigoribacteri	0	0	0	0	0	0	0	0	0	0	0	0	0	0	0	0	0	0	0	0	0	0	0
Herbiconiux	0	0	0	0	0	0	0	0	0	0	0	0	0	0	0	0	0	0	0	0	0	0	0
Serinicoccus	2	628	81	99	0	8	0	7	0	26	30	34	17	0	4	10	121	0	1	0	0	4	0
Janibacter	5	54	242	83	10	33	25	39	88	28	43	39	50	8	3	200	307	85	40	7	35	5	11
Ornithinimicro	2	71	90	63	35	21	11	16	32	40	45	52	27	7	12	23	0	24	8	4	172	14	0
Ornithinibacter	0	21	2	8	0	0	10	3	0	0	3	4	4	0	0	1	0	0	0	0	0	0	1
Knoellia	0	0	0	0	0	15	2	8	4	0	10	2	3	5	0	15	0	0	0	8	0	0	0
Tetrasphaera	0	0	2	18	1	0	3	0	0	2	3	2	3	0	0	1	1	0	4	1	0	0	0
Phycicoccus	1	0	0	0	0	0	0	0	0	0	0	0	1	0	0	0	0	0	0	0	19	3	0
Arsenicicoccu	0	0	0	0	0	0	0	0	12	0	0	24	13	0	0	0	0	0	0	0	1	0	0
Terracoccus	0	0	0	0	0	0	0	0	0	0	0	0	0	0	0	0	0	0	0	0	0	0	0
Intrasporangi	0	0	0	0	0	0	0	0	0	0	0	0	0	0	0	0	0	0	0	0	0	2	0
Kribbia	0	0	0	0	0	0	0	0	0	0	0	0	0	0	0	0	0	0	0	0	0	0	0
Ornithinicoccu	0	0	0	19	0	0	0	0	0	1	0	0	0	1	0	0	0	0	0	0	0	0	0
Aquipuribacter	0	0	0	0	0	0	0	0	0	0	0	0	0	0	0	0	0	0	0	0	0	0	0
Terrabacter	0	0	0	0	0	0	0	0	0	0	0	0	0	0	0	0	0	0	0	0	0	0	0

Genera	C10_S27	C11	C12	C13	C14	C15	C6	C7	C7_S3	C8	C8_S11	C9	C9_S19	D10_S28	D7_S4	D8_S12	D9_S20	E10_S29	E7_S5	E8_S13	E9_S21	F10_S30	F7_S6
Brachybacteri	184	0	75	0	1	5	2	0	1	330	0	3	99	169	41	98	103	287	0	4228	0	765	69
Devriesea	4	0	18	0	0	2	0	0	0	5	0	0	3	1	0	0	2	14	0	237	0	13	2
Dermabacter	7	1	0	0	1	0	2	0	0	2	0	0	85	23	17	0	69	92	0	0	0	23	65
Helcobacillus	0	0	0	0	0	0	0	0	0	0	0	0	0	0	0	0	0	0	0	0	0	0	0
Kocuria	1551	32	568	15	32	26	20	2	3034	142	20	121	964	713	138	808	819	1763	212	400	19	8196	2791
Micrococcus	761	13	1211	12	728	31	30	3	158	18	6	90	402	386	323	12684	721	8898	72	14465	58	6174	2359
Rothia	7	1	68	169	4	0	4	0	0	1	25	0	2	39	0	23	7	42	3	0	16	79	35
Citricoccus	0	0	0	0	0	0	0	0	0	0	0	0	0	0	0	0	0	0	0	1	0	0	6
Arthrobacter	7	1	56	3	1	0	0	0	0	0	0	1	0	9	0	201	0	37	0	5	4	25	26
Nesterenkonia	9	0	0	0	0	0	0	0	0	2	0	0	0	16	0	95	0	432	46	0	0	15	0
Garicola	0	0	0	0	0	0	0	0	0	0	0	0	0	0	0	0	0	0	0	0	0	0	1
Enteractinoco	0	1	0	0	0	0	0	0	0	0	0	0	0	0	0	0	0	0	0	0	3	0	24
Yaniella	0	0	0	0	0	0	0	0	0	0	0	0	0	0	0	0	0	0	0	0	0	0	0
Tersicoccus	0	0	0	0	0	0	0	0	0	0	0	0	0	0	0	0	0	0	0	0	0	0	0
Sinomonas	0	0	0	0	0	0	0	0	0	0	0	0	0	0	0	0	0	0	0	0	0	0	0
Zhihengliuella	3	0	0	0	0	0	0	0	0	0	0	0	0	0	0	0	0	0	0	0	0	0	0
Renibacterium	0	0	0	0	0	0	0	0	0	0	0	0	0	0	0	0	0	0	0	0	0	0	0
Dermacoccus	0	0	0	0	0	0	0	0	0	1	0	0	0	0	0	0	0	0	19	0	0	73	0
Kytococcus	21	4	75	0	1	0	2	1	0	0	2	5	170	82	34	155	36	759	0	4253	0	151	184
Barrientosiimo	0	0	0	0	0	8	0	0	0	0	0	0	132	0	0	87	0	8	9	0	0	62	11
Yimella	0	0	0	0	0	0	0	0	0	0	0	0	0	0	0	0	0	0	0	0	0	0	0
Calidifontibact	0	0	0	0	0	0	0	0	0	0	0	0	0	0	0	0	3	0	0	0	0	0	0
Flexivirga	0	0	0	0	0	0	0	0	0	0	0	0	0	0	0	0	0	0	0	0	0	0	0
Luteipulveratu	0	0	0	0	0	0	0	0	0	0	0	0	0	0	0	0	0	0	8	0	0	10	0
Microbacteriu	103	0	41	0	0	2	0	0	0	3	0	1	158	15	5	28	0	22	0	18	2	105	37
Zimmermanne	9	1	0	0	0	0	0	0	0	2	0	0	0	0	0	0	0	90	0	0	0	1	0
Agrococcus	27	0	0	0	0	2	0	0	0	0	0	2	200	11	0	116	8	1	0	0	0	26	31
Leucobacter	2	0	0	0	0	0	0	0	0	1	0	0	0	0	0	125	0	0	0	0	0	0	4
Agromyces	33	0	0	0	0	0	0	0	0	0	0	0	0	0	0	5	0	0	0	0	2	3	4
Naasia	0	0	0	0	0	0	0	0	0	0	0	0	0	0	0	0	0	0	0	0	0	0	0
Curtobacteriu	3	2	0	0	0	2	0	0	20	0	0	1	10	0	0	0	0	0	0	0	0	7	24
Amnibacterium	0	0	0	0	0	0	0	0	0	0	0	0	0	0	0	0	0	0	0	0	0	0	0
Rhodoluna	0	0	0	0	0	0	0	0	0	0	0	0	93	0	0	0	41	0	0	0	0	0	9
Alpinimonas	0	0	0	0	0	0	0	0	0	0	0	0	0	0	0	0	137	0	0	0	0	0	0
Gryllotalpicola	0	0	0	0	0	0	0	0	0	0	0	0	0	0	0	0	0	0	0	0	0	0	0
Leifsonia	0	0	0	0	0	0	0	0	0	0	0	0	16	0	0	0	0	0	0	0	0	0	0
Microcella	0	0	0	0	0	0	0	0	0	0	0	0	0	0	0	0	0	0	0	0	0	1	0
Diaminobutyri	0	0	0	0	0	0	0	0	0	0	0	0	0	0	0	2	0	0	0	0	0	0	0
Gulosibacter	0	0	0	0	0	0	0	0	0	0	0	0	0	0	0	0	0	0	0	0	0	6	4
Lysinimonas	27	0	0	0	0	0	0	0	0	0	0	0	0	0	0	0	0	0	0	0	0	0	0
Homoserinibac	0	0	0	0	0	0	0	0	0	0	0	0	0	0	0	0	0	0	0	0	0	2	0
Frigoribacteri	0	0	0	0	0	0	0	0	0	0	0	0	0	0	0	0	0	0	0	0	0	1	0
Herbiconiux	0	0	0	0	0	0	0	0	0	0	0	0	0	0	0	0	0	0	0	0	0	0	0
Serinicoccus	111	0	0	1	1	0	0	0	0	0	1	0	141	0	11	0	89	35	22	1	4	308	217
Janibacter	63	0	0	0	0	2	1	0	0	18	0	1	200	7	0	43	72	244	60	40	0	269	195
Ornithinimicro	351	0	22	1	0	0	1	0	3	8	0	0	457	3	5	35	128	178	6	2	34	343	501
Ornithinibacter	0	0	0	0	0	1	0	0	0	1	0	0	11	0	0	0	779	0	0	0	0	55	0
Knoellia	11	0	50	0	0	0	0	0	0	0	0	0	99	0	0	149	0	6	0	1	0	13	25
Tetrasphaera	38	0	0	0	0	0	0	0	0	1	0	0	1520	49	0	0	24	1	0	0	1	4	1
Phycicoccus	0	1	0	0	0	0	0	0	0	0	0	0	30	2	0	0	0	0	0	0	0	3	0
Arsenicicoccu	0	0	0	0	0	0	0	0	0	0	0	0	2	0	0	0	0	0	0	0	0	0	0
Terracoccus	0	0	0	0	0	0	0	0	0	0	0	0	0	0	10	0	0	0	0	0	0	0	0
Intrasporangi	0	0	0	0	0	0	0	0	0	0	0	0	0	0	0	0	0	0	0	0	0	0	0
Kribbia	0	0	0	0	0	0	0	0	0	0	0	0	0	0	0	0	0	0	0	0	0	0	0
Ornithinicoccu	8	0	1	0	0	0	0	0	0	0	0	0	160	0	0	0	0	32	0	0	0	0	0
Aquipuribacter	0	0	0	0	0	0	0	0	0	0	0	0	0	0	0	0	0	0	0	0	0	0	0
Terrabacter	0	0	0	0	0	0	0	0	0	0	0	0	0	0	0	0	0	0	0	0	0	0	0

[illegible]

Fluviicola	[illegible]
Prevotella	[illegible]
Alloprevotella	[illegible]
Paraprevotella	[illegible]
Bacteroides	[illegible]
Porphyromona	[illegible]
Paludibacter	[illegible]
Dysgonomona	[illegible]
Tannerella	[illegible]
Macellibacterc	[illegible]
Parabacteroid	[illegible]
Petrimonas	[illegible]
Proteiniphilum	[illegible]
Phocaeicola	[illegible]
Alistipes	[illegible]
Mucinivorans	[illegible]
Alkalitalea	[illegible]
Mariniphaga	[illegible]
Sphingobacte	[illegible]
Nubsella	[illegible]
Pseudosphing	[illegible]
Arcticibacter	[illegible]
Olivibacter	[illegible]
Pedobacter	[illegible]
Mucilaginibact	[illegible]
Parapedobact	[illegible]
Flavisolibacter	[illegible]
Cnuella	[illegible]
Sediminibacte	[illegible]
Parasegetibac	[illegible]
Segetibacter	[illegible]
Flavitalea	[illegible]
Arachidicoccu	[illegible]
Lacibacter	[illegible]
Filimonas	[illegible]
Ferruginibacte	[illegible]
Flavihumibact	[illegible]
Chitinophaga	[illegible]
Terrimonas	[illegible]
Hydrotalea	[illegible]
Niabella	[illegible]
Niastella	[illegible]
Taibaiella	[illegible]
Rubrivirga	[illegible]
Rubricoccus	[illegible]
Haliscomenob	[illegible]
Phaeodactylib	[illegible]
Aureispira	[illegible]
Paracoccus	[illegible]
Rubellimicrob	[illegible]
Amaricoccus	[illegible]
Paenirhodoba	[illegible]
Haematobacte	[illegible]
Roseicyclus	[illegible]
Gemmobacter	[illegible]
Falsirhodobac	[illegible]
Boseongicola	[illegible]
Frigidibacter	[illegible]
Citreicella	[illegible]
Rhodobacter	[illegible]
Tabrizicola	[illegible]
Pseudorhodob	[illegible]
Pseudovibrio	[illegible]
Profundibacte	[illegible]
Ruegeria	[illegible]
Rubribacteriu	[illegible]
Pannonibacter	[illegible]
Agaricicola	[illegible]
Jannaschia	[illegible]
Defluviimonas	[illegible]
Wenxinia	[illegible]
Hasllibacter	[illegible]
Ketogulonicig	[illegible]
Oceanicola	[illegible]
Methylobacter	[illegible]
Microvirga	[illegible]
Psychroglacie	[illegible]
Pseudaminobac	[illegible]
Mesorhizobiu	[illegible]
Hoeflea	[illegible]
Phyllobacteriu	[illegible]
Aquamicrobiu	[illegible]
Aminobacter	[illegible]
Bosea	[illegible]
Oligotropha	[illegible]
Bradyrhizobiu	[illegible]
Afipia	[illegible]
Salinarimonas	[illegible]
Nitrobacter	[illegible]
Tardiphaga	[illegible]
Rhodopseudo	[illegible]
Devosia	[illegible]
Hyphomicrobi	[illegible]
Pelagibacter	[illegible]
Prosthecomic	[illegible]
Rhodoplanes	[illegible]
Pedomicrobiu	[illegible]
Aquabacter	[illegible]
Filomicrobium	[illegible]
Shinella	[illegible]
Ciceribacter	[illegible]
Rhizobium	[illegible]
Neorhizobium	[illegible]
Ensifer	[illegible]
Kaistia	[illegible]
Aureimonas	[illegible]
Jiella	[illegible]
Fulvimarina	[illegible]
Ochrobactrum	[illegible]
Falsochrobact	[illegible]
Pseudochrobe	[illegible]
Camelimonas	[illegible]
Chelatococcu	[illegible]
Vasilyevaea	[illegible]
Phreatobacter	[illegible]
Hartmannibac	[illegible]
Variibacter	[illegible]
Alsobacter	[illegible]
Methylocystis	[illegible]
Pleomorphom	[illegible]
Methylopila	[illegible]
Roseiarcus	[illegible]
Methyloceanib	[illegible]
Labrys	[illegible]
Azorhizobium	[illegible]
Ancylobacter	[illegible]
Starkeya	[illegible]
Pseudolabrys	[illegible]
Xanthobacter	[illegible]
Altererythroba	[illegible]
Porphyrobacte	[illegible]
Erythromicrob	[illegible]
Croceicoccus	[illegible]
Erythrobacter	[illegible]
Sphingomona	[illegible]
Rhizorhapis	[illegible]
Novosphingob	[illegible]
Hephaestia	[illegible]
Sphingobium	[illegible]
Rhizorhabdus	[illegible]
Sphingopyxis	[illegible]
Sandaracinob	[illegible]
Blastomonas	[illegible]
Parablastomo	[illegible]
Sphingomicro	[illegible]
Parasphingopy	[illegible]
Phenylobacter	[illegible]
Brevundimona	[illegible]
Caulobacter	[illegible]
Asticcacaulis	[illegible]
Roseomonas	[illegible]
Craurococcus	[illegible]
Humitalea	[illegible]
Paracraurococ	[illegible]
Acetobacter	[illegible]
Belnapia	[illegible]
Endobacter	[illegible]
Acidisphaera	[illegible]
Acidomonas	[illegible]
Asaia	[illegible]
Swaminathani	[illegible]
Rhodovarius	[illegible]
Saccharibacte	[illegible]
Gluconobacter	[illegible]
Rubritepida	[illegible]
Rhodopila	[illegible]
Skermanella	[illegible]
Niveispirillum	[illegible]
Defluviicoccu	[illegible]
Desertibacter	[illegible]
Azospirillum	[illegible]
Dongia	[illegible]
Novispirillum	[illegible]
Elstera	[illegible]
Pelagibius	[illegible]
Nitrospirillum	[illegible]
Rhodocista	[illegible]
Reyranella	[illegible]
Ferrovibrio	[illegible]
Taonella	[illegible]
Geminicoccus	[illegible]
Parvularcula	[illegible]
Pseudomonas	[illegible]
Cellvibrio	[illegible]
Azomonas	[illegible]
Serpens	[illegible]
Rhizobacter	[illegible]
Thiopseudomo	[illegible]
Azorhizophilus	[illegible]
Permianibacte	[illegible]
Azotobacter	[illegible]
Acinetobacter	[illegible]
Enhydrobacter	[illegible]
Moraxella	[illegible]
Alkanindiges	[illegible]
Psychrobacter	[illegible]
Perlucidibaca	[illegible]
Thermomonas	[illegible]
Lysobacter	[illegible]
Luteimonas	[illegible]
Pseudoxanth	[illegible]
Pseudofulvimo	[illegible]
Arenimonas	[illegible]
Chiayiivirga	[illegible]
Stenotrophom	[illegible]
Mizugakiibacte	[illegible]
Silanimonas	[illegible]
Aquimonas	[illegible]
Vulcaniibacter	[illegible]
Wohlfahrtiimo	[illegible]
Rehaibacteriu	[illegible]
Dokdonella	[illegible]
Rudaea	[illegible]
Aspromonas	[illegible]
Xanthomonas	[illegible]
Steroidobacte	[illegible]
Solimonas	[illegible]
Povalibacter	[illegible]
Nevskia	[illegible]
Hydrocarboni	[illegible]
Panacagrimor	[illegible]
Fontimonas	[illegible]
Yokenella	[illegible]
Pseudocitroba	[illegible]
Providencia	[illegible]
Escherichia_S	[illegible]
Enterobacter	[illegible]
Citrobacter	[illegible]
Siccibacter	[illegible]
Erwinia	[illegible]
Leclercia	[illegible]
Pantoea	[illegible]
Proteus	[illegible]
Buchnera	[illegible]
Sodalis	[illegible]
Klebsiella	[illegible]
Pluralibacter	[illegible]
Serratia	[illegible]
Cronobacter	[illegible]
Rosenbergiell	[illegible]
Arsenophonus	[illegible]
Samsonia	[illegible]
Mangrovibacte	[illegible]
Raoultella	[illegible]
Franconibacte	[illegible]
Shimwellia	[illegible]
Kluyvera	[illegible]
Tatumella	[illegible]
Budvicia	[illegible]
Ewingella	[illegible]
Salmonella	[illegible]
Cosenzaea	[illegible]
Obesumbacte	[illegible]
Pectobacteriu	[illegible]
Moellerella	[illegible]

Rheinheimera	77	10	2	118	28	3	0	3	8	0	1	0	0	7	25	2	0	42	2	0	0	2	1	0	5	20	0	3	3	2	1	0	0	3	0	6	2	6	11	1	0	11	0	0	0	0	0	0	0	6	0	5	0	94	5	9	73	116	53	2	0	1	27	887
Thiovirga	0	0	0	0	0	2	0	0	0	0	0	0	0	0	0	0	0	0	0	0	0	0	0	0	0	0	0	0	0	0	0	0	0	0	0	0	0	0	0	0	0	0	0	0	0	0	0	0	0	0	0	0	0	0	0	0	0	0	0	0	0	0	0	0
Kushneria	4	1	0	6	0	3	15	5	0	0	4	22	0	0	0	0	0	7	0	63	0	0	0	0	27	0	0	0	11	0	0	0	2	1	0	19	0	0	8	0	0	0	0	0	0	0	0	0	0	594	0	0	0	0	0	0	0	12	0	3	0	5	2	0
Zymobacter	3	0	0	0	0	0	0	0	0	0	0	0	0	0	0	0	0	0	0	0	0	0	0	0	0	0	0	0	0	0	0	0	0	0	0	0	0	0	0	0	0	0	0	0	0	0	0	0	0	0	0	0	0	0	0	0	0	0	0	0	0	0	0	0
Halomonas	0	15	0	1	0	0	0	0	0	0	0	0	0	0	0	0	0	0	1	0	1	0	0	0	0	0	0	0	0	3	0	0	0	0	0	0	0	0	0	0	0	0	0	0	0	0	0	0	0	0	0	0	0	0	8	3	0	10	34	3	0	0	1	0
Salinicola	0	0	0	14	0	0	0	0	0	0	0	0	0	0	0	0	0	0	0	0	0	0	0	0	0	0	0	0	1	0	0	0	0	0	0	0	0	0	0	0	0	5	0	0	0	0	0	0	0	0	0	0	0	0	0	0	0	0	0	0	0	0	0	0
Carnimonas	0	0	0	0	0	0	0	0	0	0	0	0	0	0	0	0	0	0	0	0	0	0	0	0	0	0	0	0	0	0	0	0	0	0	0	0	0	0	0	0	0	0	0	0	0	0	0	0	0	0	0	0	0	0	0	0	0	0	0	0	0	0	3	12
Modicisalibact	0	0	0	0	0	0	0	0	0	0	0	0	0	0	0	0	0	0	0	0	0	0	0	0	0	0	0	0	0	0	0	0	0	0	0	0	0	0	0	0	0	0	0	0	0	0	0	0	0	0	0	0	0	0	0	0	0	0	0	0	0	0	1	0
Nitrincola	0	0	0	0	0	0	0	0	0	0	0	0	0	0	0	0	0	0	0	0	0	0	2	0	0	0	0	0	0	0	0	0	0	0	0	0	0	3	0	0	0	0	0	0	0	0	0	0	0	0	0	0	0	0	0	10	9	0	0	0	1287	0	0	0
Hahella	0	0	0	4	0	0	0	0	0	0	0	0	0	0	0	0	0	1	0	0	0	0	0	0	0	0	0	0	0	0	0	0	0	0	0	0	0	2	0	0	0	3	0	0	0	0	0	0	0	0	0	0	0	0	0	0	0	0	0	0	1	0	0	0
Alcanivorax	0	0	0	0	0	0	0	0	0	0	0	0	7	0	0	0	0	0	0	0	0	0	0	0	0	0	0	0	0	0	0	0	0	0	0	0	0	0	0	0	0	0	0	0	0	0	0	0	0	0	0	0	0	0	0	0	0	6	0	0	0	0	0	0
Haemophilus	15	45	0	47	2	0	0	1	1	5	0	2	0	0	0	1	0	3	0	4	11	0	0	0	1	0	0	3	3	19	7	2	0	0	0	2	0	0	0	7	0	9	0	0	0	0	0	0	0	0	0	0	0	19	11	0	6	7	0	0	3	0	21	7
Aggregatibact	16	17	0	16	0	1	0	0	0	59	2	0	0	0	4	0	0	0	0	0	0	0	0	0	0	0	0	0	0	1	1	0	0	0	0	0	0	0	0	4	0	7	0	0	0	0	0	0	0	0	0	0	0	13	0	0	0	0	0	0	0	0	3	14
Actinobacillus	2	0	0	0	0	0	0	0	0	0	0	0	0	0	0	0	0	0	0	0	0	0	0	0	0	0	0	0	0	0	0	0	0	0	0	0	0	0	0	0	0	0	0	0	0	0	0	0	0	0	0	0	0	0	0	0	0	0	0	0	0	0	0	0
Pasteurella	6	0	0	0	0	0	0	0	0	0	0	0	0	0	0	0	0	0	0	0	0	0	0	0	0	0	0	0	0	0	0	0	0	0	0	0	0	0	0	0	0	0	0	0	0	0	0	0	0	0	0	0	0	0	0	0	0	0	0	0	0	0	0	0
Phocoenobact	0	1	0	0	0	0	0	0	0	0	0	0	0	0	0	0	0	0	0	0	0	0	0	0	0	0	0	0	0	0	1	0	0	0	0	0	0	0	0	0	0	0	0	0	0	0	0	0	0	0	0	0	0	0	0	0	0	0	0	0	0	0	0	0
Frederiksenia	0	0	0	0	0	0	0	0	0	1	0	0	0	0	0	0	0	0	0	0	0	0	0	0	0	0	0	0	0	0	0	0	0	0	0	0	0	0	0	0	0	0	0	0	0	0	0	0	0	0	0	0	0	0	0	0	0	0	0	0	0	0	0	0
Alishewanella	24	1	0	28	1	15	5	102	53	0	8	85	58	26	47	76	1	47	1	56	13	18	11	2	25	60	9	22	11	1	7	0	1	17	211	23	15	3	0	1	0	7	0	0	0	0	0	0	0	7	0	2	0	16	9	2	19	12	30	0	0	0	18	188
Marinobacter	1	0	0	0	0	0	0	0	0	0	0	0	0	0	0	0	0	1	0	0	0	0	0	0	0	0	0	0	0	0	0	0	0	0	0	0	0	0	0	0	0	0	0	0	0	0	0	0	0	0	0	0	0	0	0	0	0	0	0	0	0	0	0	1
Haliea	0	0	0	0	0	0	0	0	0	0	0	11	0	0	0	0	0	0	0	0	2	0	0	0	0	0	0	0	0	0	4	0	0	0	0	0	0	0	0	0	0	0	0	0	0	0	0	0	0	0	0	0	0	0	0	0	0	0	0	0	0	0	0	0
Catenovulum	0	0	0	0	0	0	0	0	0	0	0	0	0	0	0	0	0	0	0	0	0	0	0	0	0	0	0	0	0	0	0	0	0	0	0	0	0	0	0	0	0	0	0	0	0	0	0	0	0	0	0	0	0	0	0	0	0	0	0	0	0	0	0	0
Shewanella	4	10	3	13	55	1	0	0	0	0	0	0	0	0	1	0	0	0	0	0	0	0	0	0	0	0	0	0	0	0	0	1	0	0	0	0	0	0	0	0	0	9	0	2	0	0	0	0	0	1	0	2	0	0	1	3	10	12	20	0	0	0	14	0
Maricurvus	1	0	0	0	0	1	0	0	0	0	0	0	0	0	0	0	0	0	0	0	0	0	0	0	0	0	0	0	0	0	0	0	0	0	0	0	0	0	0	0	0	0	0	0	0	0	0	0	0	0	0	0	0	0	1	1	1	0	0	0	0	0	2	0
Paraferrimona	0	0	0	0	0	0	0	0	0	0	0	0	0	0	0	0	0	0	0	0	0	0	0	0	0	0	0	0	0	0	0	0	0	0	0	0	0	0	0	0	0	0	0	0	0	0	0	0	0	0	0	0	0	0	0	0	0	0	1	0	0	0	0	0
Idiomarina	0	0	0	0	0	0	0	0	0	0	0	0	0	0	0	0	0	0	0	0	0	0	0	0	0	0	0	0	0	0	0	0	0	0	0	0	0	0	0	0	0	0	0	0	0	0	0	0	0	0	0	0	0	0	0	0	0	0	0	0	0	0	0	0
Aliidiomarina	0	0	0	0	0	0	0	0	0	0	0	0	0	0	0	0	0	0	0	0	0	0	0	0	0	0	0	0	0	0	0	0	0	0	0	0	0	0	0	0	0	0	0	0	0	0	0	0	0	0	0	0	0	0	0	0	0	0	0	0	0	0	0	1
Aeromonas	9	30	0	64	61	33	6	8	2	0	7	19	16	5	1	22	0	41	1	0	15	2	0	3	0	9	7	16	3	4	5	8	1	6	0	11	7	2	5	0	0	53	0	1	0	0	0	0	0	0	0	2	0	440	1	19	48	33	51	0	0	1	20	36
Tolumonas	0	0	0	7	0	2	0	0	0	0	0	2	0	0	0	0	0	7	0	0	0	0	0	0	0	0	0	0	0	0	0	0	0	0	0	0	0	0	0	0	0	10	0	0	0	0	0	0	0	0	0	0	0	7	0	0	0	7	1	0	0	0	0	0
Oceanisphaer	0	0	0	0	0	0	0	0	0	0	0	0	0	0	0	0	0	0	0	0	0	0	0	0	0	0	0	0	0	0	0	0	0	0	0	0	0	0	0	0	0	0	0	0	0	0	0	0	0	0	0	0	0	0	0	0	0	0	0	0	0	0	0	0
Succinivibrio	0	0	0	0	0	0	0	0	0	0	0	0	0	0	0	0	60	0	0	0	0	0	0	0	0	0	0	0	0	0	0	0	0	0	0	0	0	0	0	0	0	0	0	0	0	0	0	0	0	0	0	0	0	0	0	0	0	0	0	0	0	0	0	0
Legionella	0	1	0	11	0	0	0	0	1	0	0	0	8	0	0	0	0	0	0	0	0	1	0	0	0	0	0	0	0	0	1	0	0	0	0	4	0	1	0	3	0	7	0	0	0	0	0	0	0	5	0	1	0	0	0	4	0	0	0	0	0	2	0	0
Aquicella	0	0	0	0	0	0	0	0	0	0	0	0	0	0	0	12	0	0	0	0	0	0	0	0	0	0	0	0	0	0	0	0	0	0	0	0	0	0	0	0	0	0	0	0	0	0	0	0	0	0	0	0	0	0	0	0	0	0	0	0	0	0	0	0
Vibrio	0	0	2	0	0	0	0	3	1	4	0	2	2	1	8	0	0	6	0	0	0	5	0	0	0	0	0	0	0	0	0	0	0	0	0	0	0	2	0	0	0	0	0	0	0	0	0	0	0	0	0	0	0	0	0	0	14	2	3	0	0	0	4	0
Allomonas	0	0	0	0	0	2	1	5	0	8	1	1	2	3	13	0	0	4	0	0	0	4	0	0	0	1	0	0	1	2	0	0	0	0	0	0	0	0	0	0	0	0	0	0	0	0	0	0	0	0	0	0	0	0	0	0	5	3	1	0	0	0	1	0
Lucibacterium	0	0	0	0	0	0	0	0	0	0	0	0	0	0	0	0	0	0	0	0	0	1	0	0	0	0	0	0	0	0	0	0	0	0	0	0	0	0	0	0	0	0	0	0	0	0	0	0	0	0	0	0	0	0	0	0	0	0	0	0	0	0	0	0
Photobacteriu	0	0	0	0	0	0	0	0	0	0	0	0	0	0	0	0	0	0	0	0	0	0	0	0	0	0	0	0	0	0	1	0	0	0	0	0	0	0	0	0	0	0	0	0	0	0	0	0	0	0	0	0	0	0	0	1	0	0	0	0	0	0	0	0
Candidatus_C	0	0	0	1	0	0	0	0	0	0	0	0	0	0	0	0	0	0	0	0	0	0	0	0	0	0	0	0	0	0	0	0	0	0	0	0	0	0	0	0	0	0	0	0	0	0	0	0	0	0	0	0	0	0	0	0	0	0	0	0	0	0	0	0
Alkalimonas	0	0	0	0	0	0	0	0	0	0	0	0	0	0	0	0	0	0	0	0	0	0	0	0	0	0	0	0	4	0	0	0	0	0	0	8	0	0	0	0	0	0	0	0	0	0	0	0	0	0	0	0	0	0	0	0	0	0	0	0	0	0	0	0
Umboniibacter	0	0	0	0	0	0	0	0	0	0	0	0	0	0	0	0	0	0	0	0	0	0	0	0	0	0	0	0	0	0	0	0	0	0	0	0	0	0	0	0	0	0	0	0	0	0	0	0	0	0	0	0	0	0	0	0	0	1	0	0	0	0	0	0
Methylomonas	0	0	0	0	0	1	0	0	0	0	0	0	0	0	0	0	0	0	0	0	0	0	0	0	0	0	0	0	0	0	0	0	0	1	0	0	0	0	0	0	0	0	0	0	0	0	0	0	0	0	0	0	0	0	0	0	0	0	0	0	0	0	0	0
Methylobacter	0	0	0	0	0	0	0	0	0	0	0	1	0	0	0	0	0	0	0	0	0	0	0	0	0	0	0	0	0	0	0	0	0	0	0	0	0	0	0	0	0	0	0	0	0	0	0	0	0	0	0	0	0	0	0	0	0	0	0	0	0	0	0	0
Cardiobacteri	0	0	0	0	0	0	0	0	3	0	0	0	0	0	0	0	0	0	0	0	0	0	0	0	0	0	22	0	2	0	0	0	0	0	0	0	0	0	0	0	0	0	0	0	0	0	0	0	0	0	0	0	0	0	0	0	0	0	0	0	0	0	0	0
Salinisphaera	0	0	0	0	0	0	0	0	0	0	0	1	0	0	0	0	0	0	0	0	0	0	0	0	0	0	0	0	0	0	0	0	0	0	0	0	0	0	0	0	0	0	0	0	0	0	0	0	0	0	0	0	0	0	0	0	0	0	0	0	0	0	0	0
Methylophaga	0	0	0	0	0	0	0	0	0	0	0	0	0	0	0	0	0	0	1	0	0	0	0	0	0	0	0	0	0	0	0	0	0	0	0	0	0	0	0	0	0	0	0	0	0	0	0	0	0	0	0	0	0	0	0	0	0	0	0	0	0	0	0	0
Thiothrix	0	0	0	0	0	0	0	0	0	0	0	0	0	0	0	0	0	0	0	0	0	0	0	0	0	0	0	0	0	0	0	0	0	1	0	0	0	0	0	0	0	0	0	0	0	0	0	0	0	0	0	0	0	0	0	0	0	0	0	0	0	0	0	0
Pelomonas	313	205	6	2131	163	8	0	23	45	0	4	0	0	0	0	17	0	9	0	9	0	19	0	7	10	6	0	0	4	0	2	8	0	1	0	0	4	6	187	6	3	1061	3	79	1	0	3	0	0	1	0	4	0	327	1211	77	36	6425	409	62	1	103	350	152
Delftia	32	5	10	15	161	154	118	90	96	118	20	489	27	93	173	193	879	41	0	9	21	4	0	0	24	0	12	25	0	5	0	1	0	3	96	35	17	4	74	7	0	12	0	0	0	0	2	0	0	9	0	0	1	59	83	7	346	93	40	11	46	22	7	32
Ottowia	60	24	0	47	0	2	2	0	0	0	13	0	0	0	0	0	0	16	0	0	92	14	0	15	19	0	7	3	18	13	34	0	0	7	0	9	7	0	0	0	0	2	0	0	0	0	0	0	0	0	0	0	0	232	141	0	14	9	0	0	0	0	56	0
Pseudorhodof	13	25	0	6	0	8	0	3	0	0	2	0	0	0	0	0	0	127	1	0	2	0	0	0	0	5	1	0	0	9	2	0	0	1	0	5	0	0	0	0	0	0	0	0	0	0	1	0	0	0	0	0	0	0	0	0	0	1	0	0	0	0	20	0
Schlegelella	8	24	7	0	120	0	0	0	0	0	0	0	0	0	0	0	0	0	0	0	0	0	0	0	0	0	0	0	0	0	0	0	0	1	0	0	0	1	80	3	0	78	0	0	0	0	0	0	0	32	0	0	0	52	28	19	0	681	53	26	0	7	9	0
Diaphorobacte	1	0	0	0	0	0	0	0	0	50	0	10	3	0	0	0	0	0	0	0	19	0	0	0	0	0	0	0	0	0	0	0	0	0	0	0	1	0	0	0	0	0	0	0	0	0	0	0	0	0	1	0	0	72	0	0	0	0	0	0	0	0	0	0
Limnohabitans	8	0	0	10	0	4	0	0	0	0	0	0	0	0	0	14	0	4	0	0	0	0	0	0	0	3	0	0	0	0	0	0	0	0	0	7	0	0	22	0	0	0	0	0	0	0	0	0	0	0	0	0	0	1	0	0	0	160	0	0	0	0	0	57
Caenimonas	5	0	0	18	0	2	0	8	11	0	0	0	0	0	0	31	0	0	0	0	0	0	0	0	0	1	0	0	0	0	0	0	5	9	0	2	0	0	7	0	0	0	0	0	0	0	0	0	1	0	0	0	0	3	0	0	0	23	0	0	0	0	20	14
Comamonas	6	4	1	25	104	2	0	0	0	0	2	0	0	1	0	3	0	49	0	0	0	0	0	3	0	29	15	0	3	17	0	0	0	0	0	1	1	0	55	0	1	24	1	14	0	0	1	2	0	0	0	0	1	0	6	3	126	0	11	0	10	0	9	72
Ramlibacter	1	10	0	5	0	1	1	5	8	0	0	0	73	0	0	0	0	6	0	0	7	10	0	0	0	3	0	0	2	0	0	3	0	0	0	0	0	0	15	0	0	13	0	0	1	0	0	2	0	0	0	0	0	86	0	0	0	10	0	0	0	0	4	0
Acidovorax	3	0	1	196	0	3	0	0	12	0	0	0	0	1	0	0	0	8	0	0	21	0	0	1	1	0	9	2	1	11	0	1	0	0	0	2	0	0	0	1	0	18	0	0	0	0	0	0	0	0	0	0	0	6	12	7	64	323	0	0	0	0	15	148
Variovorax	1	5	0	0	0	0	0	0	0	0	0	0	0	0	0	0	0	0	0	0	4	0	0	0	0	0	0	0	4	17	0	0	0	0	0	0	0	1	0	0	0	0	0	0	0	0	0	0	0	0	0	0	0	0	0	0	6	0	0	0	0	0	0	0
Tepidicella	1	0	0	0	0	0	0	4	5	0	0	0	0	0	0	0	0	0	0	0	0	0	0	0	0	0	0	0	0	0	0	0	0	0	0	0	0	0	0	0	0	0	0	0	0	0	0	0	0	0	0	1	0	1	0	0	1	2	0	1	0	0	0	0
Alicycliphilus	3	0	0	21	0	0	0	0	1	0	0	3	1	0	0	0	0	0	0	0	5	0	0	0	0	0	1	1	1	0	1	1	0	1	0	1	0	0	0	0	0	5	0	0	0	0	0	0	0	0	0	0	0	2	5	3	15	33	0	0	0	0	5	39
Brachymonas	1	0	0	0	0	0	0	0	0	0	0	0	0	0	0	0	0	0	0	0	0	0	0	0	0	0	0	0	2	0	0	0	0	0	0	0	0	1	0	0	0	0	0	0	0	0	0	0	0	0	0	0	0	0	0	20	0	1	0	0	0	0	0	0
Roseateles	0	8	0	1	0	0	0	0	0	0	0	0	0	0	0	0	0	0	0	0	0	0	0	0	0	0	0	0	0	0	0	0	0	0	0	0	0	0	0	0	0	0	0	0	0	0	0	0	0	0	0	0	0	0	0	0	0	1	0	0	0	0	0	0
Zhizhongheell	0	1	1	1	0	1	0	0	0	0	0	0	0	0	0	0	0	4	0	0	0	0	0	0	0	0	0	0	0	0	0	0	0	0	0	6	0	0	1	0	0	0	0	0	0	0	2	0	0	8	0	1	0	0	17	5	49	0	0	0	0	1	0	0
Curvibacter	0	4	0	72	0	34	32	36	10	0	15	119	0	5	13	11	0	11	0	39	11	27	13	2	13	4	7	0	8	3	3	18	4	31	0	10	9	6	39	6	0	66	0	0	0	0	0	0	0	0	0	1	0	10	6	5	0	108	1	0	0	18	1	7
Hydrogenopha	0	1	0	357	0	6	0	316	2595	0	3	16	4	0	0	2	1	9	0	0	8	6	0	1	5	0	3	0	3	0	0	1	0	2	0	9	2	0	0	2	0	4	0	0	0	0	0	0	0	0	0	0	0	4	0	0	0	468	0	0	0	0	0	0
Rhodoferax	0	0	0	8	0	1	0	0	0	0	0	0	0	0	0	0	0	0	0	0	0	0	0	0	0	0	1	0	0	0	0	0	0	0	0	0	0	0	0	0	0	0	0	0	0	0	0	0	0	0	0	0	0	0	0	0	0	6	0	0	0	0	0	0
Macromonas	0	0	0	2	0	5	0	0	0	0	0	0	0	0	0	0	0	7	0	0	0	0	0	0	0	3	0	0	0	0	0	0	0	0	0	0	0	0	0	0	0	11	0	0	0	0	0	0	0	0	0	0	0	67	0	0	0	0	0	0	0	0	3	0
Malikia	0	0	0	2	0	1	0	204	204	0	0	2	4	0	0	0	0	2	0	0	0	3	0	0	0	0	1	0	0	0	1	0	0	2	0	1	0	0	0	0	0	0	0	0	0	0	0	0	0	0	0	0	0	3	0	0	0	0	0	0	0	0	0	17
Simplicispira	0	0	0	2	0	0	0	0	6	0	0	0	0	0	0	0	0	0	0	0	0	0	0	0	0	1	0	0	0	0	0	0	0	0	0	0	0	0	0	0	0	0	0	0	0	0	0	0	0	0	0	0	0	0	0	0	0	1	0	0	0	0	0	1
Melaminivora	0	0	0	0	0	0	0	0	0	0	0	0	0	5	0	0	0	0	0	0	0	0	0	0	0	0	0	0	0	0	0	0	0	0	0	0	0	0	0	0	0	0	0	0	0	0	0	0	0	0	0	0	0	0	0	0	0	0	0	0	0	0	0	0
Xenophilus	0	0	0	0	0	0	0	0	0	0	0	0	0	0	0	0	0	0	0	0	0	0	0	0	0	0	0	2	0	0	1	0	0	1	0	0	0	0	0	0	0	0	0	0	0	0	0	0	0	0	0	0	0	1	0	0	0	1	0	0	0	0	0	0
Pseudacidovo	0	0	0	0	0	0	0	0	0	0	0	0	0	0	0	0	0	0	0	0	0	0	0	0	0	0	0	0	4	0	0	0	0	0	0	0	0	0	0	0	0	0	0	0	0	0	0	0	0	0	0	0	0	0	0	0	0	0	0	0	0	0	0	0
Polaromonas	0	0	0	0	0	0	0	0	0	0	0	0	0	0	0	0	0	0	0	0	0	0	0	0	0	0	0	0	0	0	0	0	0	1	0	0	0	0	0	0	0	0	0	0	0	0	0	0	0	0	0	0	0	0	0	0	0	0	0	0	0	0	0	0
Hylemonella	0	0	0	0	0	0	0	0	0	0	0	0	0	0	0	0	0	0	0	0	0	0	0	0	0	0	0	0	0	0	0	0	0	0	0	0	0	0	0	0	0	0	0	0	0	0	0	0	0	0	0	0	0	0	0	0	0	0	0	0	0	0	1	0
Kinneretia	0	0	0	0	0	0	0	0	0	0	0	0	0	0	0	0	0	0	0	0	0	0	0	0	0	0	0	0	0	0	0	0	0	0	0	0	0	0	0	0	0	0	0	0	0	0	0	0	0	0	0	0	0	0	0	0	0	0	0	0	0	0	0	0
Limnobacter	459	37	106	2027	1563	34	5	39	15	0	5	38	50	0	0	48	61	18	0	0	0	0	14	4	10	5	11	0	5	2	14	14	6	15	0	6	3	68	184	23	0	2253	0	0	0	0	0	0	0	34	0	24	0	1387	187	310	775	3704	540	21	41	75	108	773
Chitinimonas	8	0	0	29	0	0	0	4	11	0	0	0	0	0	0	0	0	4	0	0	0	0	0	0	0	3	0	0	0	0	0	1	0	0	0	0	0	0	0	0	0	0	0	0	0	0	0	0	0	0	0	0	0	0	0	0	0	189	0	0	0	0	0	0
Ralstonia	14	70	0	40	79	9	1	5	2	0	4	28	10	3	16	13	0	5	0	23	6	28	0	3	0	2	14	0	11	3	0	6	1	0	0	10	0	1	0	0	1	24	0	0	0	0	0	1	0	2	0	0	1	32	51	2	0	78	218	7	21	0	13	145
Lautropia	13	45	0	0	0	10	0	37	0	0	4	0	0	0	0	0	70	11	0	0	40	0	0	0	0	5	0	0	2	0	0	0	0	0	0	0	0	0	7	0	0	15	0	0	3	0	0	0	0	0	0	0	0	191	0	0	0	51	1	1	0	0	10	0
Burkholderia	0	6218	13	1	0	0	0	0	0	0	0	3	0	0	0	0	0	0	0	0	0	0	0	0	0	0	0	0	0	0	0	0	0	1	0	0	0	1	12861	2550	0	19	0	0	0	0	0	0	0	0	0	1160	0	1	7375	11	85	0	0	1093	2	4165	1	1
Pandoraea	0	46	0	0	0	0	0	0	0	0	1	0	0	4	0	0	0	0	0	0	0	0	0	0	0	0	0	0	0	0	0	0	0	1	0	0	0	6	54	8	0	0	0	0	0	0	0	0	0	0	0	0	0	0	33	0	0	0	0	14	0	10	0	0
Cupriavidus	0	0	3	0	0	1	2	5	0	0	0	0	0	0	0	0	0	0	0	0	0	0	0	0	0	0	0	0	0	0	0	0	0	0	0	2	0	10	0	0	0	5	0	0	0	0	0	0	0	0	0	2	0	0	0	5	8	0	0	0	0	0	0	0
Polynucleobac	0	0	0	76	0	1	0	0	0	0	0	0	0	0	0	0	0	0	0	0	0	0	0	0	0	5	0	0	2	0	0	0	0	0	0	0	0	0	16	0	0	0	0	0	0	0	0	0	0	0	0	0	0	0	0	0	0	8	0	0	0	0	0	25
Aquabacteriu	13	6	65	129	40	0	1	26	203	5	0	0	10	0	1	1	0	10	0	0	0	3	1	0	1	0	0	2	1	0	0	0	0	0	0	2	0	18	10	3	0	3	0	0	0	0	0	0	0	25	0	19	0	10	0	138	521	162	10	0	13	0	43	79
Rubrivivax	2	0	0	20	12	0	0	1	2	0	0	0	0	0	0	0	0	0	0	0	0	0	0	0	0	0	0	0	1	0	0	0	0	0	0	0	0	0	0	0	0	23	0	0	0	0	0	0	0	0	0	0	0	7	5	0	0	13	1	1	0	0	2	7
Mitsuaria	10	5	0	27	0	1	0	4	10	0	0	0	0	0	0	0	0	4	0	0	0	0	0	0	0	5	1	0	0	0	0	0	0	0	0	0	0	0	0	0	0	4	0	0	0	0	0	0	0	0	0	0	0	12	0	0	0	2	1	0	0	1	27	3
Ideonella	5	4	16	100	1	7	3	113	432	0	3	39	17	1	0	17	1	13	0	0	9	3	0	0	0	5	10	3	3	0	1	2	0	3	0	2	3	8	16	0	0	1	0	0	0	0	0	0	0	2	0	2	0	0	0	68	155	110	0	0	1	0	0	9
Piscinibacter	3	0	1	14	0	1	1	21	52	0	1	5	1	1	0	4	0	0	0	0	2	2	0	0	0	0	2	1	0	0	1	0	0	2	0	0	1	0	0	0	0	2	0	0	0	0	0	0	0	2	0	2	0	1	0	3	8	5	1	0	0	0	0	2
Tepidimonas	5	0	0	0	0	0	0	0	0	0	0	0	0	0	0	0	0	0	0	0	0	0	4	2	0	0	1	0	0	0	0	0	0	0	0	0	0	4	6	0	0	1	0	0	0	0	0	0	0	50	0	3	0	2	0	3	62	49	17	31	0	3	0	0
Aquincola	0	5	0	0	0	0	0	0	0	0	5	0	0	0	0	0	0	7	0	0	0	0	0	0	0	6	11	0	0	3	4	0	0	3	0	0	0	0	0	0	0	0	0	0	0	0	0	0	0	0	0	0	0	0	0	0	0	0	0	0	0	0	0	0
Sphaerotilus	0	0	0	229	0	0	0	0	0	0	0	0	0	0	0	0	0	0	0	0	0	0	0	0	0	2	0	0	16	34	22	90	29	39	0	0	0	0	1	0	0	0	0	0	0	0	0	0	0	0	0	0	0	34	0	0	0	383	0	0	0	0	0	37
Leptothrix	0	0	0	152	0	0	0	0	0	0	0	0	0	0	0	0	0	0	0	0	0	0	0	0	0	0	0	0	0	0	0	0	0	0	0	0	0	0	2	0	0	0	0	0	0	0	0	0	0	1	0	0	0	0	0	0	1	240	0	0	0	0	0	1
Inhella	0	0	0	24	0	0	0	89	172	0	0	0	0	0	0	0	1	5	0	0	0	0	0	0	0	5	0	0	0	0	0	0	0	0	0	0	0	0	0	0	0	0	0	0	0	0	0	0	0	0	0	0	0	0	0	0	0	3	0	0	0	0	1	0
Rivibacter	0	0	0	0	0	0	0	1	0	0	0	0	0	0	0	0	0	0	0	0	0	0	0	0	0	0	0	0	0	0	0	0	0	0	0	0	0	0	0	0	0	6	0	0	0	0	0	0	0	0	0	0	0	0	0	0	0	0	0	0	0	0	0	0
Methylibium	0	0	0	0	0	0	0	0	0	0	0	0	0	0	0	0	0	0	0	0	0	0	0	0	0	0	0	0	0	0	0	0	0	0	0	0	0	0	0	0	0	0	0	0	0	0	0	0	0	0	0	0	0	0	0	0	0	0	0	0	0	0	0	20
Massilia	19	35	3	33	0	5	2	3	4	0	0	7	0	0	0	0	0	37	0	0	5	4	0	3	3	1	0	2	2	4	1	0	0	1	0	1	2	1	0	5	3	113	1	1	0	0	0	1	0	0	0	0	1	0	13	0	2	20	12	10	0	1	26	20
Rugamonas	2	4	0	0	0	3	0	0	3	0	0	0	0	0	0	0	0	15	0	0	1	0	0	0	0	1	0	1	3	2	0	0	0	2	0	0	2	0	0	0	0	3	1	0	0	0	1	1	0	0	0	0	0	33	0	0	1	0	1	0	0	0	11	3
Noviherbaspiri	7	8	0	25	0	3	0	1	3	0	0	0	28	0	0	0	0	0	0	0	0	0	0	0	0	8	1	0	8	0	0	0	0	1	0	0	0	0	0	0	0	1	0	0	0	0	0	0	0	0	0	0	0	2	16	0	0	8	10	0	0	0	0	1
Undibacterium	9	0	0	0	0	0	0	0	0	0	0	0	0	0	0	0	0	0	0	0	0	0	0	0	0	0	0	0	0	0	0	0	0	0	0	2	0	0	0	0	0	0	0	0	0	0	0	0	0	0	0	0	0	0	0	0	0	13	2	0	0	0	4	4
Oxalicibacteri	0	1	0	0	0	0	0	0	0	0	0	0	0	0	0	0	0	0	0	0	0	0	0	0	0	0	0	0	0	0	0	0	0	0	0	0	0	0	0	0	0	1	0	0	0	0	0	0	0	0	0	0	0	0	0	0	0	0	0	0	0	0	0	0
Janthinobacte	0	0	0	0	0	0	0	0	0	0	0	0	0	0	0	0	3	0	0	0	0	0	0	0	0	0	0	0	0	0	0	0	0	0	0	0	0	0	0	0	0	0	0	0	0	0	0	0	0	0	0	0	0	0	0	0	0	0	0	0	0	0	0	0
Pseudodugan	0	0	0	0	0	0	0	0	0	0	0	0	0	0	0	0	0	1	0	0	0	0	0	0	0	0	0	0	0	0	0	0	0	0	0	0	0	0	0	0	0	0	0	1	0	0	0	0	0	0	0	0	0	0	0	0	0	0	0	0	0	0	0	0
Herbaspirillum	0	0	0	0	0	0	0	0	0	0	0	0	0	0	0	0	0	0	0	0	0	0	0	0	0	0	0	0	0	0	0	0	0	0	0	0	0	0	0	0	0	7	0	0	0	0	0	0	0	0	0	0	0	0	0	0	0	0	0	0	0	0	0	0
Parapusillimo	5	0	0	0	0	0	0	0	1	3	0	0	0	0	0	0	0	0	0	0	0	0	0	0	0	0	0	0	0	0	0	0	0	0	0	0	0	0	0	0	0	14	0	0	0	0	0	0	0	0	0	0	0	0	0	0	0	1	0	0	0	0	1	0
Azohydromon	0	13	0	0	0	0	3	0	2	0	0	0	0	0	0	0	0	0	0	7	0	0	0	0	0	4	3	6	0	0	3	2	0	0	0	0	0	0	12	0	0	0	0	0	0	0	0	0	0	0	0	0	0	0	0	0	0	0	0	0	0	0	3	2
Achromobacte	0	2	0	9	0	0	0	0	1	3	0	0	0	1	0	0	0	0	0	0	3	0	0	0	1	0	0	0	0	0	0	0	0	0	0	0	0	0	0	0	0	11	0	0	0	0	0	2	0	0	0	0	0	0	0	0	49	0	0	0	0	0	0	1
Alcaligenes	0	0	0	0	0	9	7	16	7	0	0	8	7	9	32	39	0	5	0	0	0	7	1	0	0	1	0	6	10	0	0	2	1	2	0	2	3	2	3	0	2	5	0	0	0	0	0	0	0	0	0	0	0	0	0	0	0	0	0	0	0	0	0	0
Kerstersia	0	0	0	0	0	0	3	0	0	0	0	0	0	0	0	0	0	0	0	0	0	0	0	0	0	0	0	0	0	0	0	0	0	0	0	0	0	0	0	0	0	0	0	0	0	0	0	2	0	0	0	0	0	0	0	0	0	0	0	0	0	0	0	0
Eoetvoesia	0	0	0	0	0	0	0	0	0	0	0	0	0	3	0	0	0	0	0	0	0	0	0	0	0	0	0	0	0	0	0	0	0	0	0	0	0	0	0	0	0	0	0	0	0	0	0	0	0	0	0	0	0	0	0	0	0	0	0	0	0	0	0	0
Pigmentiphag	0	0	0	0	0	0	0	0	0	0	0	0	0	0	0	0	0	0	0	0	0	0	0	6	0	0	0	0	0	0	0	0	0	0	0	0	0	0	0	0	0	0	0	0	0	0	0	0	0	0	0	0	0	0	0	0	0	0	0	0	0	0	0	0
Paenalcaligen	0	0	0	0	0	0	0	0	0	0	0	0	0	0	0	0	0	0	0	0	0	0	0	0	0	0	0	0	1	0	0	0	0	0	0	0	0	0	0	0	5	0	0	4	2	0	0	0	0	0	0	0	0	0	0	0	0	0	2	0	0	0	0	0
Pelistega	0	0	0	0	0	0	0	0	0	0	0	0	0	0	0	0	0	0	0	0	0	0	0	0	0	0	0	0	0	0	0	0	0	0	0	0	0	0	0	0	0	0	0	0	0	0	0	0	0	0	0	0	0	8	0	0	0	0	0	0	0	0	0	0
Advenella	0	0	0	0	0	0	0	0	0	0	0	0	0	0	0	0	0	0	0	0	0	0	0	0	0	0	0	0	0	0	0	0	0	0	0	0	0	0	0	0	0	0	0	0	0	0	0	0	0	0	0	0	0	0	0	0	0	1	0	0	0	0	0	0
Castellaniella	0	0	0	0	0	0	0	0	0	0	0	0	0	0	0	0	0	0	0	0	0	0	0	0	0	0	0	0	0	0	0	0	0	0	0	0	0	0	0	0	0	0	0	0	0	0	0	0	0	0	0	0	0	0	0	0	0	0	0	0	0	0	0	1
Bordetella	0	0	0	0	0	0	0	0	0	0	0	0	0	0	0	0	0	0	0	0	0	0	0	0	0	0	0	0	0	0	0	0	0	0	0	0	0	0	0	0	0	0	0	0	0	0	0	0	0	0	0	0	0	0	0	0	0	0	0	0	0	0	0	0
Oligella	0	0	0	0	0	0	0	0	0	0	0	0	0	0	0	0	0	0	0	0	0	0	0	0	0	0	0	0	0	0	0	0	0	0	0	0	0	0	0	1	0	0	0	0	0	0	0	0	0	0	0	0	0	0	0	0	0	0	0	0	0	0	0	0
Parasutterella	0	0	0	0	0	0	0	2	0	0	0	0	0	0	0	0	0	0	0	0	0	0	0	0	0	0	0	0	0	0	0	0	0	0	0	0	0	0	0	0	0	0	0	0	0	0	0	0	0	0	0	0	0	0	0	0	0	0	0	0	0	0	0	0
Sutterella	0	0	0	0	0	0	0	0	0	0	0	0	0	0	0	0	0	0	0	0	0	0	0	0	0	0	0	0	1	0	0	0	0	0	0	0	0	0	0	0	0	0	0	0	0	0	0	0	0	0	0	0	0	0	0	0	0	0	0	0	0	0	0	0
Methyloversat	36	0	1	88	0	3	0	0	0	0	0	0	0	0	0	0	0	0	0	23	0	0	0	0	0	0	0	0	0	0	0	0	0	0	0	0	0	3	0	0	0	54	0	0	0	0	0	0	0	48	0	0	0	29	0	14	43	78	119	0	0	7	13	0
Thauera	4	0	0	99	0	3	0	7	0	0	0	0	0	0	0	9	80	23	0	0	23	15	6	11	0	3	12	4	20	32	4	0	0	8	89	0	3	0	0	0	0	15	0	0	1	0	0	1	0	0	0	0	0	160	0	0	0	30	4	0	0	0	41	0
Azoarcus	0	10	0	0	0	0	0	0	0	0	0	0	0	0	0	0	0	0	0	0	0	0	0	0	0	0	0	0	0	0	0	0	0	0	0	0	0	0	0	0	0	0	0	0	0	0	0	0	0	0	0	0	0	0	28	0	0	0	0	0	0	0	0	0
Zoogloea	0	0	1	0	0	0	10	0	0	0	0	25	3	0	0	0	0	0	0	0	2	0	0	3	0	1	0	0	0	0	0	13	0	5	0	0	0	2	0	0	0	0	0	0	0	0	0	0	0	0	0	2	0	0	8	8	0	0	0	0	0	0	14	0
Ferribacterium	0	0	0	0	0	35	6	2	0	0	2	2	0	2	0	7	0	15	0	20	10	0	1	0	5	3	6	8	10	0	1	3	2	11	0	0	7	0	0	0	0	1	0	0	0	0	0	0	0	0	0	0	0	0	3	0	0	1	2	0	0	0	2	1
Dechloromona	0	0	0	0	0	9	8	2	0	0	0	0	0	4	0	20	0	8	0	2	4	0	2	0	8	0	3	0	3	0	2	1	0	8	0	0	1	0	0	0	0	3	0	0	0	0	0	0	0	0	0	0	0	0	7	0	0	3	2	0	0	1	3	1
Propionivibrio	0	0	0	0	0	1	0	0	0	0	0	0	0	0	0	0	0	5	0	0	0	0	0	0	10	0	0	0	0	0	0	0	0	0	0	0	0	0	0	0	0	1	0	0	0	0	0	0	0	0	0	0	0	0	0	0	0	0	11	0	0	0	0	0
Georgfuchsia	0	0	0	0	0	1	0	0	0	0	0	0	0	0	0	0	0	0	0	0	2	0	0	0	0	0	0	0	2	0	0	2	0	0	0	1	6	0	0	0	0	0	0	0	0	0	0	0	0	0	0	0	0	0	0	0	0	0	0	0	0	0	0	0
Azovibrio	0	0	0	0	0	8	0	0	0	0	0	0	0	0	0	0	0	0	0	0	0	0	0	0	0	6	0	0	0	0	0	0	0	0	0	0	0	0	0	0	0	0	0	0	0	0	0	0	0	0	0	0	0	0	0	0	0	0	0	0	0	0	0	0
Uliginosibacte	0	0	0	0	0	0	0	0	0	0	0	0	0	0	0	0	0	0	0	0	2	0	0	0	0	0	0	0	0	0	0	0	0	0	0	0	0	0	0	0	0	0	0	0	0	0	0	0	0	0	0	0	0	0	0	0	0	0	0	0	0	0	0	0
Azospira	0	0	0	0	0	0	0	0	0	0	0	0	0	0	0	0	0	0	0	0	0	0	0	0	0	0	0	0	0	0	0	0	0	0	0	0	0	0	0	0	0	0	0	0	0	0	0	0	0	0	0	0	0	0	0	0	0	9	0	0	0	0	0	0
Denitratisoma	0	0	0	0	0	0	0	0	0	0	0	0	0	0	0	0	0	0	0	0	0	0	0	0	0	0	0	0	0	0	0	0	0	0	0	0	0	0	0	0	0	0	0	0	0	0	0	0	0	0	0	0	0	0	0	0	0	0	0	0	0	0	0	0
Methylotenera	18	0	0	203	0	0	0	0	4	0	0	0	0	0	0	0	0	0	0	0	0	0	0	0	0	2	0	0	0	0	0	5	0	0	0	3	0	0	7	0	0	0	0	0	0	0	0	0	0	0	0	0	0	0	0	0	8	41	0	0	0	0	0	88
Methylobacillu	3	0	0	0	0	0	0	0	0	0	0	0	0	0	0	0	0	0	0	0	0	0	0	0	0	0	0	0	0	0	0	0	0	0	0	0	0	3	0	0	0	0	0	0	0	0	0	0	0	0	0	1	0	0	0	0	0	0	0	0	0	0	0	0
Methylophilus	0	0	0	5	0	0	0	0	0	0	0	0	0	0	0	0	0	0	0	0	0	0	0	0	0	0	0	0	0	0	0	0	0	0	0	0	0	0	0	0	0	5	0	6	0	0	3	0	0	0	0	0	2	0	0	0	0	0	0	0	0	0	0	17
Morococcus	24	57	0	0	0	4	0	1	0	86	0	0	0	6	34	0	0	4	0	0	1	0	1	4	0	1	1	17	9	5	0	0	4	0	50	7	3	0	25	4	0	6	1	148	0	0	0	0	0	0	0	0	0	288	52	0	55	0	0	5	7	0	16	58
Neisseria	9	162	0	8	0	1	1	5	4	1	1	1	0	0	3	6	27	7	0	8	41	1	0	0	0	2	0	4	4	18	16	0	2	1	4	1	0	0	1	0	0	3	0	369	0	0	0	0	0	0	0	0	0	265	25	0	10	3	2	1	1	0	32	31
Kingella	1	20	0	1	0	0	1	1	4	0	0	0	0	0	0	0	0	28	1	0	0	0	0	0	0	8	0	0	2	0	0	1	0	2	0	0	0	0	0	0	0	0	0	0	0	0	0	0	0	0	0	0	0	3	0	0	0	0	0	0	0	0	12	0
Snodgrassella	1	0	0	0	0	0	0	0	0	0	0	0	0	0	0	0	0	0	0	0	0	0	0	0	0	0	1	0	0	0	0	0	0	0	0	0	0	0	0	0	0	0	0	0	0	0	0	0	0	0	0	0	0	0	0	0	0	0	0	0	0	0	0	0
Simonsiella	2	0	0	4	0	0	0	0	0	0	0	0	0	0	0	0	0	0	0	0	0	0	0	0	0	0	0	0	0	0	0	0	0	0	0	0	0	0	0	0	0	0	0	0	0	0	0	0	0	0	0	0	0	0	0	0	0	2	0	0	0	0	1	0
Vogesella	3	4	0	19	0	0	0	0	0	0	1	0	0	0	0	0	0	8	0	0	0	0	0	0	0	1	0	0	0	0	0	0	0	0	0	0	0	0	0	0	0	0	2	0	0	0	0	0	0	0	0	0	0	0	0	0	0	257	0	0	0	0	14	37
Eikenella	0	0	0	7	0	0	0	5	0	0	0	0	0	0	0	0	0	0	0	0	0	0	0	0	0	0	0	0	2	0	0	0	0	0	0	8	0	0	0	0	0	0	0	0	0	0	0	0	0	0	0	0	0	0	0	0	0	0	0	0	0	0	10	0
Leeia	0	0	0	0	0	2	11	0	0	0	0	0	0	0	0	0	0	0	0	0	0	0	0	0	0	0	0	0	0	0	0	0	0	5	0	0	0	0	0	0	0	0	0	0	0	0	0	0	0	0	0	0	0	0	0	0	0	0	0	0	0	0	0	0
Vitreoscilla	0	0	0	0	0	0	0	0	1	0	0	0	0	0	0	0	0	0	0	0	0	0	0	0	0	0	0	0	1	0	0	0	0	0	0	0	0	0	0	0	0	0	0	0	0	0	0	0	0	0	0	0	0	0	0	0	0	0	0	0	0	0	0	0
Chitinilyticum	0	0	0	0	0	0	0	0	0	0	0	0	12	0	0	0	0	0	0	0	0	0	0	0	0	0	0	0	0	0	0	0	0	0	0	0	0	0	0	0	0	0	0	0	0	0	0	0	0	0	0	0	0	0	0	0	0	0	0	0	0	0	0	0
Bergeriella	0	0	0	0	0	0	0	0	0	0	0	0	0	0	0	0	5	0	0	0	0	0	0	0	0	0	0	0	0	0	2	0	0	0	0	0	0	0	0	0	0	0	0	0	0	0	0	0	0	0	0	0	0	0	0	0	0	0	0	0	0	0	0	0
Uruburuella	0	0	0	0	0	0	0	0	0	0	0	0	0	0	0	0	0	0	0	0	5	0	0	0	0	0	0	0	0	0	0	0	0	0	0	0	0	0	0	0	0	8	0	0	0	0	0	0	0	0	0	0	0	0	0	0	0	0	0	0	0	0	1	0
Aquaspirillum	0	0	0	0	0	0	0	0	0	0	0	0	0	0	0	0	0	0	0	0	0	0	0	0	0	0	0	0	4	0	0	0	0	0	0	0	0	0	0	0	0	0	0	0	0	0	0	0	0	0	0	0	0	0	0	0	0	0	0	0	0	0	2	0
Rivicola	0	0	0	0	0	0	0	0	0	0	0	0	0	0	0	0	0	0	0	0	0	0	0	0	0	0	0	0	3	0	0	0	0	0	0	0	0	0	0	0	0	0	0	0	0	0	0	0	0	0	0	0	0	0	0	0	0	0	0	0	0	0	6	0
Gulbenkiania	0	0	0	0	0	0	0	0	0	0	0	0	0	0	0	0	0	0	0	0	0	0	0	0	0	0	0	0	0	0	0	0	0	0	0	0	0	0	0	0	74	0	111	1066	155	12	56	55	14	0	12	0	89	0	0	0	0	0	0	0	0	0	2	0
Pseudogulben	0	0	0	0	0	0	0	0	0	0	0	0	0	0	0	0	0	0	0	0	0	0	0	0	0	0	0	0	0	0	0	0	0	0	0	0	0	0	0	0	0	0	0	0	0	0	0	0	0	0	0	0	0	0	0	0	0	4	0	0	0	0	1	0
Hydrogenophi	6	0	0	0	0	0	0	0	0	0	0	0	0	0	0	0	0	0	0	0	0	0	0	0	0	0	0	0	0	0	0	0	0	0	0	0	0	0	0	0	0	0	0	0	0	0	0	0	0	0	0	0	0	0	47	0	0	0	7	0	0	0	14	0
Thiobacillus	0	0	0	0	0	0	0	0	0	0	0	0	21	0	0	0	0	0	0	0	0	0	0	0	0	0	0	0	0	0	0	0	0	0	0	0	0	0	0	0	0	0	0	0	0	0	0	0	0	0	0	0	0	0	0	0	0	0	0	0	0	0	0	0
Tepidiphilus	0	0	0	0	0	0	0	0	0	0	0	0	13	0	0	0	0	0	0	0	0	0	0	0	0	0	0	0	0	0	0	0	0	0	0	0	0	0	0	0	0	0	0	0	0	0	0	0	0	0	0	0	0	0	0	0	0	0	0	0	0	0	0	0
Nitrosospira	0	12	0	0	0	0	0	0	0	0	0	0	0	0	0	0	0	0	0	0	0	0	0	0	0	0	0	0	0	0	0	0	0	0	0	0	0	0	0	0	0	0	0	0	0	0	0	0	0	0	0	0	0	0	0	0	0	0	0	0	0	0	0	0
Nitrosomonas	0	0	0	0	0	0	0	0	0	0	0	0	0	0	0	0	0	4	0	0	0	0	0	0	0	0	0	0	0	0	0	0	0	0	0	0	0	0	0	0	0	0	0	0	0	0	0	0	0	0	0	0	0	0	0	0	0	0	0	0	0	0	0	0
Sideroxydans	0	1	0	0	0	0	0	0	0	0	0	0	0	0	0	0	0	0	0	0	0	0	0	0	0	0	0	0	0	0	0	0	0	0	0	0	0	0	0	0	0	0	0	0	0	0	0	0	0	0	0	0	0	0	0	0	0	0	0	0	0	0	0	0
Ferrovum	0	1	1	0	0	0	0	0	0	0	0	0	0	0	0	0	0	0	0	0	0	0	0	0	0	0	0	0	0	0	0	0	0	0	0	0	0	0	0	2	0	42	0	0	0	0	0	0	0	0	0	0	0	0	0	0	0	0	0	0	0	0	0	9
Angiococcus	1	0	0	0	0	0	0	0	0	0	0	0	0	0	0	0	0	0	0	0	0	0	0	0	0	0	0	0	3	0	0	0	0	0	0	0	0	0	0	0	0	0	0	0	0	0	0	0	0	0	0	0	0	0	0	0	0	0	0	0	0	0	0	0
Myxococcus	9	1	0	0	0	0	0	0	0	0	0	0	0	0	0	0	0	0	0	0	0	0	0	0	0	0	0	0	0	0	0	0	0	0	0	0	0	0	0	0	0	0	0	0	0	0	0	0	0	0	0	0	0	0	0	0	0	0	0	0	0	0	0	0
Aggregicoccus	0	4	0	0	0	0	0	0	0	0	0	0	0	0	0	0	0	0	0	0	0	0	0	0	0	3	0	0	0	8	0	0	0	0	0	0	0	0	0	0	0	0	0	0	0	0	0	0	0	0	0	0	0	0	0	0	0	0	0	0	0	0	0	0
Pyxidicoccus	0	0	0	0	0	0	0	27	0	0	0	0	0	0	0	0	0	0	0	0	0	0	0	0	0	0	0	0	1	0	0	0	3	0	0	0	0	0	0	0	0	0	0	0	0	0	0	0	0	0	0	0	0	0	0	0	0	0	0	0	0	0	0	21
Corallococcus	0	0	0	0	0	0	0	0	0	0	0	0	0	0	0	0	0	0	0	0	0	0	0	0	0	0	0	0	1	0	0	0	0	0	0	0	0	0	0	0	0	0	0	0	0	0	0	0	0	0	1	0	0	0	0	0	0	0	0	0	0	0	0	0
Anaeromyxob	5	19	0	0	0	0	0	0	0	0	0	0	0	0	0	16	0	3	0	0	0	5	4	0	0	0	0	0	0	3	0	0	0	0	0	0	0	0	0	0	0	2	0	0	0	0	0	0	0	0	0	0	0	98	0	0	0	0	0	0	0	0	1	5
Archangium	0	0	0	0	0	0	0	0	0	0	0	0	0	0	0	0	0	0	0	0	0	0	0	0	0	0	0	0	0	0	1	0	0	0	0	0	0	0	0	0	0	0	0	0	0	0	0	0	0	0	0	0	0	0	0	0	0	0	0	0	0	0	0	0
Cystobacter	0	0	0	0	0	0	0	0	0	0	0	0	0	0	0	0	0	0	0	0	0	0	0	0	0	0	0	0	0	0	0	0	0	0	0	0	0	0	0	0	0	0	0	0	0	0	0	0	0	0	0	0	0	2	0	0	0	0	0	0	0	0	0	0
Hyalangium	0	0	0	0	0	0	0	0	0	0	0	0	0	0	0	0	0	0	0	0	0	0	0	0	0	0	0	0	0	0	0	0	0	0	0	0	0	0	0	0	0	0	0	0	0	0	0	0	0	0	0	0	0	0	0	0	0	0	0	0	0	0	0	0
Melittangium	0	0	0	0	0	0	0	0	0	0	0	0	0	0	0	0	0	0	0	0	0	0	0	0	0	0	0	0	0	0	0	0	0	0	0	0	0	0	0	0	0	0	0	0	0	0	0	0	0	0	0	0	0	0	0	0	0	0	0	0	0	0	0	0
Vulgatibacter	0	0	0	0	0	0	0	0	0	0	0	0	0	0	0	0	0	0	0	0	0	0	0	0	0	0	0	0	0	0	0	0	0	0	0	0	0	0	0	0	0	3	0	0	0	0	0	0	0	0	0	0	0	0	0	0	0	0	0	0	0	0	2	0
Byssovorax	5	0	0	19	0	0	0	0	0	0	0	0	0	0	0	0	0	0	0	0	0	0	0	0	0	0	0	0	0	0	0	0	0	0	0	0	0	0	0	0	0	14	0	0	0	0	0	0	0	0	0	0	0	58	0	0	0	0	0	0	0	0	0	4
Sorangium	0	0	0	5	0	0	0	0	0	0	0	0	0	0	0	0	0	0	0	0	0	0	0	0	1	0	0	0	2	0	0	0	4	0	0	3	0	0	0	0	0	0	0	0	0	0	0	0	0	0	0	0	0	2	0	0	0	0	0	0	1	0	0	0
Chondromyce	0	0	0	0	0	0	0	0	0	0	0	0	0	0	0	0	0	0	0	0	0	0	0	0	0	0	0	0	2	0	0	0	0	0	0	0	0	0	0	0	0	0	0	0	0	0	0	0	0	0	0	0	0	0	0	0	0	0	0	0	0	0	0	0
Minicystis	0	0	0	0	0	0	0	0	0	0	0	0	0	0	0	0	0	0	0	0	0	0	0	0	0	0	0	0	0	0	0	0	0	0	0	0	0	0	0	0	0	3	0	0	0	0	0	0	0	0	0	0	0	0	0	0	0	0	0	0	0	0	0	0
Labilithrix	2	0	2	44	0	1	0	0	0	0	0	0	0	0	0	0	0	0	0	0	0	0	0	0	0	0	0	0	0	0	0	0	0	0	0	0	0	0	1	0	0	2	0	0	0	0	0	0	0	0	0	0	0	43	0	0	0	20	0	0	0	0	0	0
Enhygromyxa	6	0	0	0	0	0	0	0	0	0	0	0	0	0	0	0	0	0	0	0	0	0	0	0	0	0	0	0	0	0	0	0	0	0	0	0	0	0	0	0	0	0	0	0	0	0	0	0	0	0	0	0	0	0	0	0	0	0	0	0	0	0	0	0
Nannocystis	0	10	0	0	0	0	0	0	0	0	0	0	0	0	0	0	0	0	0	0	0	0	0	0	0	0	0	0	0	0	0	0	0	0	0	0	0	0	0	0	0	0	0	0	0	0	0	0	0	0	0	0	0	0	0	0	0	0	0	0	0	0	0	0
Pseudenhygro	0	0	0	0	0	0	0	0	0	0	0	0	0	0	0	0	0	4	0	0	0	0	0	0	0	0	0	0	0	0	0	0	0	0	0	0	0	0	0	0	0	0	0	0	0	0	0	0	0	0	0	0	0	0	0	0	0	0	0	0	0	0	0	0
Kofleria	0	0	0	0	0	0	0	0	0	0	0	0	0	0	0	0	0	0	0	0	0	0	0	0	0	0	0	0	0	0	0	0	0	0	0	0	0	0	0	0	0	0	0	0	0	0	0	0	0	0	0	0	0	0	0	0	0	0	0	0	0	0	0	0
Sandaracinus	0	0	0	0	0	0	0	0	9	0	0	0	0	0	0	0	0	0	0	0	1	0	0	0	0	0	0	0	0	0	0	0	0	0	0	0	0	0	0	0	0	0	0	0	0	0	0	0	0	0	0	0	0	0	0	0	0	0	0	0	0	0	0	0
Geobacter	2	0	0	0	0	0	0	0	0	0	0	0	0	2	0	0	0	0	0	0	0	0	0	0	0	0	0	0	0	0	0	0	0	0	0	0	0	0	0	0	0	0	0	0	0	0	0	0	0	0	0	0	0	0	0	0	0	0	0	0	0	0	0	0
Bacteriovorax	6	0	0	40	0	0	0	0	0	0	0	0	16	0	0	0	0	2	0	0	0	0	0	0	0	0	0	0	1	0	0	0	0	0	0	0	0	0	0	0	0	0	0	0	0	0	0	0	0	0	0	0	0	0	0	0	0	8	0	0	0	0	7	0
Peredibacter	0	0	0	0	0	0	0	0	1	6	0	2	1	0	0	0	0	0	0	0	0	0	0	0	0	0	0	0	0	0	0	0	0	0	1	0	0	0	0	0	0	0	0	0	0	0	0	0	0	0	0	0	0	0	0	0	0	0	0	0	0	0	0	0
Vampirovibrio	0	0	0	0	0	0	0	0	0	0	0	2	0	0	0	0	0	0	0	0	0	0	0	0	0	0	0	0	0	0	0	0	0	0	0	0	0	0	0	0	0	0	0	0	0	0	0	0	0	0	0	0	0	0	0	0	0	0	26	0	0	0	0	0
Bdellovibrio	0	0	0	0	0	0	0	0	0	0	0	0	0	0	0	0	0	1	0	0	0	0	0	0	0	8	0	0	0	0	0	0	0	1	0	8	0	0	0	0	0	0	0	0	0	0	0	0	0	0	0	0	0	0	0	0	0	0	0	0	0	0	0	0
Desulfoprunu	0	0	0	0	0	0	0	0	0	0	0	0	0	0	0	0	0	0	0	0	0	0	0	0	0	0	0	0	4	0	0	0	0	0	0	0	0	0	0	0	0	0	0	0	0	0	0	0	0	0	0	0	0	0	0	0	0	0	0	0	0	0	0	0
Bilophila	0	0	0	0	0	0	0	0	0	0	0	0	0	0	0	0	0	0	0	0	0	0	0	0	0	0	0	0	2	0	0	0	0	0	0	0	0	0	0	0	0	0	0	0	0	0	0	0	0	0	0	0	0	0	0	0	0	0	0	0	0	0	0	0
Deferrisoma	0	0	0	0	0	0	0	0	0	0	0	0	0	0	0	0	0	0	0	0	0	12	0	0	0	0	0	0	0	0	0	0	0	0	0	0	0	0	0	0	0	0	0	0	0	0	0	0	0	0	0	0	0	0	0	0	0	0	0	0	0	0	0	0
Campylobacte	1	14	0	0	0	2	0	0	0	0	0	0	6	0	0	0	75	5	0	0	1	0	0	0	0	0	0	0	0	0	1	0	0	0	0	0	0	0	0	1	0	0	0	0	0	0	0	0	0	0	0	0	0	18	0	3	0	0	29	0	0	0	10	0
Arcobacter	0	0	0	17	0	0	0	0	0	0	0	4	0	0	0	0	0	4	0	0	0	0	0	0	0	0	0	0	0	3	0	0	0	0	0	0	1	0	0	0	0	2	0	0	0	0	0	0	0	0	0	0	0	0	0	0	0	0	0	0	0	0	3	0
Sulfuricurvum	0	0	0	0	0	1	2	0	0	0	1	0	0	0	0	0	0	0	0	0	0	0	0	0	0	0	0	0	0	0	0	0	0	0	0	0	0	0	0	0	0	0	0	0	0	0	0	0	0	0	0	0	0	0	0	0	0	0	0	0	0	0	0	0
Wolinella	0	0	0	0	0	0	0	0	0	0	0	0	0	0	0	0	0	0	0	0	0	0	0	0	0	0	0	0	0	0	0	0	0	0	0	0	0	0	0	0	0	0	0	0	0	0	0	0	0	0	0	0	0	0	0	0	0	0	0	0	0	0	18	0
Oligoflexus	0	0	0	0	0	0	0	0	0	0	0	0	0	0	0	0	0	0	0	0	0	0	0	0	0	1	0	0	0	0	0	0	0	0	0	0	0	0	0	0	0	0	0	0	0	0	0	1	0	0	0	0	0	0	0	0	0	0	7	0	0	0	8	0
Staphylococcu	6550	7548	82	3052	227	161	325	772	142	239	34	400	931	1068	857	1792	4227	547	13	811	2268	1331	1312	33	567	1226	603	3690	8776	161	7039	119	51	3070	1572	429	516	982	30690	83592	39346	5197	32638	38200	59668	52988	52373	32672	59736	4128	33941	15285	33963	924	33259	69365	27595	5269	901	1479	8146	89795	13379	1037
Salinicoccus	57	235	0	361	0	0	0	10	2	0	0	0	0	0	0	0	0	0	1	0	0	3	0	0	0	0	0	0	12	2	2	1	1	0	0	2	0	0	61	5	0	66	0	0	0	0	0	0	0	0	0	0	0	0	0	0	1	230	22	0	0	4	56	53
Macrococcus	72	53	0	5	0	0	0	5	0	0	0	5	0	4	4	0	0	3	0	0	0	5	0	0	0	0	0	10	8	14	31	1	0	5	0	0	0	0	39	3	0	27	0	8	0	0	0	1	0	0	1	0	0	0	0	0	29	0	0	0	0	5	69	87
Jeotgalicoccus	38	10	0	0	0	0	3	9	5	0	0	0	0	0	0	8	0	12	1	0	1	0	7	0	0	0	5	11	12	3	2	1	0	4	0	0	0	0	8	0	0	6	0	0	0	0	0	0	0	0	1	0	0	0	0	0	79	0	7	0	0	0	118	8
Nosocomiicoc	0	0	0	16	0	0	0	0	0	0	0	0	0	0	0	0	0	0	0	0	0	0	0	0	0	0	0	0	0	0	0	0	0	0	0	0	0	0	0	0	0	0	0	23	0	0	0	0	0	0	1	0	0	0	0	0	0	0	0	0	0	0	0	0
Bacillus	138	159	27	61	0	1081	428	885	209	653	189	1517	2299	113	379	546	2509	581	15	45	225	399	351	337	144	48	956	485	331	390	171	1181	19	435	1682	516	87	97	123	6	2	417	3	66	2	0	9	8	1	80	1	13	0	174	66	101	603	132	57	42	27	12	215	265
Anoxybacillus	26	0	0	0	0	0	0	0	0	0	1	0	0	0	0	18	0	0	0	1	0	0	0	0	0	0	0	0	0	0	0	0	0	0	0	2	0	1	0	0	0	0	0	27	0	0	0	0	0	0	0	0	0	0	0	0	0	0	5	0	0	0	62	14
Domibacillus	9	81	0	0	0	8	3	35	13	0	2	0	0	0	0	0	0	1	0	0	2	2	0	1	0	0	0	0	1	8	1	0	0	1	0	0	0	0	0	0	0	34	0	0	0	0	1	0	0	0	0	0	0	36	0	0	164	0	3	1	0	1	34	0
Falsibacillus	1	0	0	0	0	1	0	0	1	0	0	1	7	0	0	0	2	0	0	0	0	0	0	1	0	0	0	0	0	0	0	0	0	1	0	2	0	0	0	0	0	12	0	0	0	0	0	0	0	0	0	0	0	0	0	0	0	0	0	0	0	0	1	1
Geobacillus	0	0	0	0	0	0	0	1	0	0	0	0	0	1	0	1	0	0	0	0	3	0	0	0	4	0	3	3	0	0	0	0	0	0	0	0	0	0	23	0	0	0	0	0	0	0	0	0	0	0	0	0	0	0	0	0	39	0	0	0	0	2	0	0
Saccharococc	0	0	0	0	0	0	0	0	0	0	0	0	0	0	0	1	0	0	0	0	0	0	0	0	0	0	0	0	0	0	0	0	0	0	0	0	0	0	1	0	0	0	0	0	0	0	0	0	0	0	0	0	0	0	0	0	0	0	0	0	0	0	0	1
Caldibacillus	0	0	0	0	0	0	0	0	0	0	0	0	0	0	0	0	0	0	0	0	0	0	0	0	0	0	0	0	0	0	2	0	0	0	0	0	0	0	0	0	0	0	0	0	0	0	0	0	0	0	0	0	0	0	0	0	0	0	0	0	0	0	0	0
Fictibacillus	0	0	0	0	0	0	0	0	0	0	0	0	0	0	0	0	0	0	0	0	0	0	0	0	0	0	0	0	0	0	0	0	0	0	0	0	0	0	0	0	0	0	0	0	0	0	0	0	0	0	0	0	0	0	0	0	0	0	0	0	0	0	5	0
Anaerobacillus	0	0	0	0	0	0	0	0	0	0	0	0	0	0	0	0	0	0	0	0	0	0	0	0	0	0	0	0	0	0	0	0	0	0	0	0	0	0	0	0	0	0	0	0	0	0	0	0	0	0	0	0	0	0	0	0	0	0	0	0	0	0	2	0
Paenibacillus	6	7	0	0	0	7	1	2	1	0	0	1	1	2	6	8	138	0	0	0	0	0	0	0	0	0	0	0	0	1	3	0	0	0	0	0	0	0	0	2	0	14	0	0	0	0	0	0	0	0	0	0	0	0	3	0	0	0	0	5	0	3	4	0
Brevibacillus	0	19	0	0	179	0	1	7	1	0	0	11	0	0	15	10	32	1	0	0	3	0	0	0	0	1	2	0	0	0	0	0	0	1	0	1	0	1	0	0	0	1	0	12	0	0	0	0	0	0	0	0	0	0	0	6	0	0	0	0	0	0	1	0
Saccharibacill	0	22	0	0	0	0	0	0	0	0	3	0	0	0	0	0	0	0	0	0	0	0	0	0	0	0	0	0	0	0	0	0	0	0	0	0	0	0	0	0	0	0	0	0	0	0	0	0	0	0	0	0	0	0	0	0	0	0	0	0	0	0	3	1
Fontibacillus	0	0	0	0	0	0	0	0	0	0	0	0	16	0	13	40	42	2	0	0	0	4	0	0	0	0	0	0	0	1	0	0	0	0	0	1	0	0	0	0	0	0	0	0	0	0	0	0	0	0	0	0	0	0	0	0	0	0	0	0	0	0	0	0
Thermobacillu	0	0	0	0	0	0	0	0	0	0	0	0	0	0	0	0	0	0	0	0	1	0	0	0	0	0	0	0	0	0	0	0	0	0	0	0	0	0	0	0	0	0	0	0	0	0	0	0	0	0	0	0	0	0	0	0	0	0	0	0	0	0	0	0
Chryseomicro	59	97	0	159	0	6	0	3	3	0	0	0	0	3	0	68	40	19	0	0	2	18	0	9	0	7	2	5	14	0	5	4	0	3	0	0	0	0	135	19	2	584	3	0	0	0	0	0	0	0	2	0	0	324	0	0	0	133	14	5	0	11	125	228
Planococcus	49	26	0	0	70	8	3	0	7	0	0	0	0	0	0	21	0	8	0	0	0	0	0	0	0	0	0	0	2	0	0	0	0	3	0	0	2	0	0	0	0	48	0	0	0	0	0	0	0	0	0	0	0	35	0	9	0	0	2	0	0	0	50	0
Planomicrobiu	22	14	0	19	0	1	1	12	3	0	0	0	13	0	0	12	0	0	1	0	4	2	2	0	0	0	0	0	0	2	0	0	0	0	0	0	0	0	0	2	0	24	0	0	0	0	0	0	0	0	0	0	0	53	0	0	0	0	0	0	0	1	31	6
Lysinibacillus	9	4	0	13	0	1	0	4	0	0	0	0	0	0	0	0	0	5	0	0	0	0	0	0	0	0	0	1	2	0	0	0	0	0	0	0	0	0	13	0	0	15	1	13	0	0	0	0	0	0	0	0	0	61	0	0	0	0	0	0	0	3	4	13
Solibacillus	35	23	0	0	0	0	0	0	1	0	0	6	0	0	0	22	0	6	0	0	0	0	0	0	0	0	3	0	0	0	0	0	0	1	0	2	1	0	4	0	0	1	0	0	0	0	0	0	0	0	0	0	0	0	0	0	0	0	2	0	0	0	31	7
Sporosarcina	3	0	0	0	0	0	0	0	0	0	0	0	0	0	0	0	0	0	0	0	0	0	0	0	0	0	0	0	0	0	0	0	0	0	0	0	0	0	0	0	0	8	0	0	0	0	0	0	0	0	0	0	0	0	0	0	0	0	0	0	0	0	1	6
Jeotgalibacillu	2	0	0	0	0	0	0	0	0	0	0	0	0	0	0	0	0	0	0	0	0	0	0	0	0	0	0	0	0	0	0	0	0	0	0	0	0	0	0	0	0	60	0	0	0	0	0	0	0	0	0	0	0	0	0	0	0	0	0	0	0	0	18	0
Chungangia	2	0	0	0	0	0	0	0	0	0	0	0	0	0	0	0	0	0	0	0	0	0	0	0	0	0	0	0	0	0	0	0	0	5	0	0	0	0	0	0	0	0	0	0	0	0	0	0	0	0	0	0	0	0	0	0	0	0	0	0	0	0	0	18
Bhargavaea	2	5	0	0	0	0	0	1	7	0	0	0	0	0	0	0	0	0	0	0	0	13	0	0	6	0	6	0	10	0	0	0	2	3	0	0	0	0	0	1	0	4	0	0	0	0	0	4	0	0	0	0	0	0	0	0	0	0	0	0	0	0	4	0
Ureibacillus	0	7	0	0	0	0	0	0	0	0	0	0	0	0	0	0	0	0	0	0	0	0	0	0	0	0	0	0	0	0	0	0	0	0	0	0	0	0	0	0	0	0	0	0	0	0	0	0	0	0	0	0	0	0	0	0	0	0	0	0	0	0	0	0
Kurthia	0	12	0	0	0	0	0	4	0	0	0	0	0	0	0	0	0	0	0	186	0	0	0	0	0	0	0	0	0	0	0	0	0	0	0	0	0	0	0	0	0	0	0	0	0	0	0	0	0	0	0	0	0	0	0	0	0	0	0	0	0	0	0	9
Caryophanon	0	0	0	2	0	1	1	8	1	0	0	0	0	0	0	27	5	1	0	0	0	0	0	0	0	1	0	0	0	0	1	0	2	0	0	0	0	0	1	0	0	0	0	0	0	0	0	0	0	0	0	0	0	1	1	0	0	0	0	1	0	0	2	50
Psychrobacillu	0	0	0	0	0	3	0	0	0	0	1	0	0	0	1	0	0	0	0	0	0	1	0	0	0	0	0	0	0	0	0	0	1	0	0	0	0	0	0	0	0	0	0	0	0	0	0	0	0	0	0	0	0	2	0	0	0	0	0	0	0	0	0	1
Rummeliibacill	0	0	0	0	0	0	2	0	0	0	0	0	0	0	0	0	0	0	0	1	0	0	0	0	0	0	0	0	0	0	0	0	0	0	0	0	0	0	0	0	0	0	0	0	0	0	0	0	0	0	0	0	0	0	0	0	0	0	0	0	0	0	0	0
Planococcace	0	0	0	0	0	0	0	0	0	0	0	0	0	0	3	0	0	0	0	0	0	0	0	0	0	0	0	0	0	0	0	0	0	0	0	0	0	0	0	0	0	0	0	0	0	0	0	0	0	0	0	0	0	0	0	0	0	0	0	0	0	0	0	0
Filibacter	0	0	0	0	0	0	0	0	0	0	0	0	0	0	0	0	0	0	0	0	0	0	0	0	0	0	0	0	0	0	0	0	0	0	0	0	0	0	0	0	0	1	0	0	0	0	0	0	0	0	0	0	0	0	0	0	0	0	0	0	0	0	0	0
Gemella	46	304	0	12	0	9	1	27	0	0	1	0	0	1	0	21	0	17	0	33	27	0	3	0	0	1	7	0	21	81	18	5	4	12	72	0	0	0	0	7	0	0	2	29	0	0	1	0	0	0	0	0	0	80	36	0	137	66	0	5	0	0	92	34
Exiguobacteri	308	113	1	106	0	12	9	7	33	0	0	10	0	0	0	33	0	104	0	29	22	10	3	0	1	13	14	0	4	0	3	22	0	1	0	8	8	4	7	0	2	238	1	0	0	0	0	0	0	8	0	0	0	45	99	0	34	206	44	15	8	3	161	154
Tumebacillus	10	2	0	15	0	0	0	17	2	0	0	0	0	0	0	13	0	5	0	0	7	0	0	0	0	0	0	0	5	1	10	0	0	0	0	0	0	0	0	0	0	11	0	0	0	0	0	0	0	0	0	0	0	0	0	0	0	0	3	0	0	0	104	11
Effusibacillus	0	0	0	0	0	0	0	0	0	0	0	0	0	0	0	0	0	0	0	0	0	0	0	0	0	5	0	0	0	0	0	0	0	0	0	0	0	0	0	0	0	0	0	0	0	0	0	0	0	0	0	0	0	0	0	0	0	0	0	0	0	0	0	0
Marinococcus	22	0	0	67	0	2	0	0	15	0	0	0	0	0	0	0	0	0	0	0	0	0	0	0	0	0	0	0	0	2	0	0	0	0	0	0	0	2	0	0	0	0	0	41	0	0	0	0	0	0	0	0	0	0	0	0	0	2	0	0	0	5	20	36
Salirhabdus	1	0	0	0	0	0	1	1	0	0	0	2	0	0	1	0	0	1	0	0	2	0	1	1	0	0	1	1	0	0	0	1	0	0	0	0	0	1	0	0	0	1	0	0	0	0	0	0	0	0	0	0	0	0	1	0	6	0	0	0	1	0	0	0
Ornithinibacill	0	2	0	0	0	0	0	0	4	0	0	0	0	0	0	0	0	0	0	0	0	0	0	0	0	3	0	0	0	0	0	0	0	0	0	0	0	0	0	0	0	0	0	0	0	0	0	0	0	0	0	0	0	0	0	0	0	0	0	0	0	0	0	0
Lentibacillus	0	0	0	1	0	0	0	1	0	0	0	0	0	0	0	0	0	0	0	0	3	0	0	0	0	0	0	0	0	0	0	0	0	1	0	0	0	0	0	0	0	0	0	0	0	0	0	0	0	0	0	0	0	0	0	0	0	1	0	0	0	0	0	0
Oceanobacillu	0	0	0	0	0	213	11	74	25	0	4	26	0	4	80	51	37	12	0	0	0	6	0	0	0	0	5	14	10	0	23	7	0	1	0	49	5	0	0	0	0	0	0	0	0	0	0	0	0	0	0	0	0	0	0	0	0	0	0	1	0	0	0	0
Paucisalibacill	0	0	0	0	0	15	4	11	2	8	2	6	6	2	0	2	1	8	0	0	1	1	1	4	7	5	9	2	7	2	2	15	0	7	13	4	3	0	0	0	0	0	0	0	0	0	0	0	0	0	0	0	0	0	0	0	0	0	0	0	0	0	1	2
Terribacillus	0	0	0	0	0	1	0	0	0	0	0	0	0	0	0	0	0	20	0	0	0	0	0	0	0	0	0	0	0	2	0	0	0	0	0	0	0	0	0	0	0	0	0	0	0	0	0	0	0	0	0	0	0	0	0	0	0	0	0	0	0	0	0	0
Halobacillus	0	0	0	0	0	0	0	1	0	0	0	0	0	0	3	2	0	0	0	0	0	0	0	0	0	0	0	0	0	3	0	0	0	0	0	0	0	0	0	0	0	0	0	0	0	0	0	0	0	0	0	0	0	0	0	0	0	0	0	0	0	0	4	3
Virgibacillus	0	0	0	0	0	0	0	0	0	0	0	2	0	0	0	0	0	0	0	0	0	0	0	0	0	0	0	0	0	0	0	0	0	0	0	0	0	0	0	0	0	0	0	0	0	0	0	0	0	0	0	0	0	0	0	0	6	0	0	0	0	0	0	0
Geomicrobium	0	0	0	0	0	0	0	0	0	0	0	0	0	0	0	0	0	0	0	0	4	0	0	0	0	0	0	0	3	0	0	0	0	2	0	0	0	0	0	0	2	6	0	0	0	0	0	0	0	0	0	0	0	0	0	0	0	0	0	0	0	0	5	0
Allobacillus	0	0	0	0	0	0	0	0	0	0	0	0	0	0	0	0	0	0	0	0	2	0	0	0	0	0	0	0	0	0	0	0	0	0	0	0	0	0	0	0	0	0	0	0	0	0	0	0	0	0	0	0	0	0	0	0	0	0	0	0	0	0	0	0
Alkalibacillus	0	0	0	0	0	0	0	0	0	0	0	0	0	0	0	0	0	0	0	0	0	0	0	0	0	0	0	0	0	0	0	0	0	0	0	0	0	1	0	0	0	0	0	0	0	0	0	0	0	0	0	0	0	0	0	0	23	42	0	0	0	0	15	0
Piscibacillus	0	0	0	0	0	0	0	0	0	0	0	0	0	0	0	0	0	0	0	0	0	0	0	0	0	0	0	0	0	0	0	0	0	0	0	0	0	1	0	0	0	0	0	0	0	0	0	0	0	0	0	0	0	0	0	0	0	0	0	0	0	0	0	0
Thalassobacill	0	0	0	0	0	0	0	0	0	0	0	0	0	0	0	0	0	0	0	0	0	0	0	0	0	0	0	0	0	0	0	0	0	0	0	0	0	0	0	0	0	30	0	0	0	0	0	0	0	0	0	0	0	0	0	0	0	0	0	0	0	0	4	0
Compostibacil	0	0	0	0	0	0	0	0	0	0	0	0	0	0	0	0	0	0	0	0	0	0	0	0	0	0	0	0	0	0	0	0	0	0	0	0	0	0	0	0	0	0	0	0	0	0	0	0	0	0	0	0	0	0	0	0	0	0	0	0	0	0	0	0
Gracilibacillus	0	0	0	0	0	0	0	0	0	0	0	0	0	0	0	0	0	0	0	0	0	0	0	0	0	0	0	0	0	0	0	0	0	0	0	0	0	0	0	0	0	0	0	0	0	0	0	0	0	0	0	0	0	0	0	0	0	0	0	0	0	0	0	0
Ammoniphilus	15	0	0	0	0	0	0	2	0	0	0	0	0	0	0	0	0	1	0	0	3	14	0	0	0	0	4	31	5	0	4	0	0	1	0	0	0	0	10	0	0	0	0	0	0	0	3	0	0	0	0	0	0	0	0	0	0	0	0	0	7	1	6
Oxalophagus	0	0	0	0	0	0	0	1	0	0	0	0	0	0	0	0	0	0	0	0	0	0	0	0	0	7	0	0	0	0	1	0	0	8	0	0	0	0	0	0	0	0	0	0	0	0	0	0	0	0	0	0	0	0	0	0	0	0	0	0	0	0	0	0
Aneurinibacillu	0	0	0	0	0	0	0	0	0	0	0	0	0	0	0	0	0	0	0	0	0	0	0	0	0	0	0	0	0	0	0	0	0	0	0	0	0	0	0	0	0	0	0	0	0	0	0	0	0	0	0	0	0	0	0	0	0	0	0	0	0	0	0	0
Brochothrix	16	0	0	0	0	0	0	0	0	0	0	0	0	0	0	0	0	0	0	0	0	9	0	0	0	0	0	0	0	0	0	0	0	0	0	0	0	0	0	0	0	0	0	0	0	0	0	0	0	0	0	0	0	0	0	0	0	0	0	0	0	0	33	0
Listeria	0	0	0	0	0	0	0	0	0	0	0	1	0	0	0	0	0	0	0	0	0	0	0	0	0	0	0	0	0	0	0	0	0	0	0	0	0	0	0	0	0	0	0	0	0	0	0	0	0	0	0	0	0	0	0	0	0	0	0	0	0	0	0	0
Planifilum	0	17	0	0	0	2	0	0	0	0	0	0	0	0	0	0	0	0	0	0	2	0	0	0	0	3	0	0	2	0	0	0	0	2	0	0	0	0	0	0	0	0	0	0	0	0	0	0	0	0	0	0	0	0	0	0	0	0	0	0	0	0	0	0
Polycladomyc	0	0	0	0	0	0	0	0	0	0	0	0	0	0	0	0	0	0	0	0	0	0	0	0	0	1	0	0	0	0	0	0	0	0	0	0	0	0	0	0	0	0	0	0	0	0	0	0	0	0	0	0	0	0	0	0	0	0	0	0	0	0	0	0
Thermoactino	0	10	0	0	0	0	2	15	1	0	0	10	0	0	0	0	0	18	0	0	3	0	0	0	0	7	0	0	3	0	1	0	0	1	0	0	0	0	0	0	0	16	0	0	0	0	0	1	0	0	1	0	0	0	0	0	0	0	0	0	0	0	3	0
Laceyella	0	0	0	0	0	0	0	5	0	0	0	0	0	0	0	0	0	7	0	0	0	0	0	0	0	0	0	6	0	0	0	0	0	2	0	5	0	0	0	0	0	0	0	0	0	0	0	0	0	0	0	0	0	0	0	0	0	0	0	0	0	0	0	0
Shimazuella	0	0	0	0	0	0	0	0	1	0	0	0	0	0	0	0	0	0	0	0	5	0	0	0	0	0	0	0	0	0	0	0	0	0	0	0	0	0	0	0	0	0	0	0	0	0	0	0	0	0	0	0	0	0	0	0	0	0	0	0	0	0	0	0
Melghirimyces	0	0	0	0	0	0	0	0	0	0	0	0	0	0	0	0	0	0	0	0	0	0	0	0	0	0	0	0	0	0	1	0	0	0	0	0	0	0	0	0	0	0	0	0	0	0	0	0	0	0	0	0	0	0	0	0	0	0	0	0	0	0	0	0

Desulfuribacill	0	0	0	0	0	10	4	7	1	1	2	9	12	5	5	7	0	7	0	0	4	6	5	0	3	2	8	0	14	1	7	20	0	25	11	9	1	0	0	0	0	0	0	0	0	0	0	0	0	0	0	0	0	0	0	0	0	0	0	0	0	0	0	0
Sporolactobac	0	0	0	0	0	0	0	1	1	0	0	0	0	0	0	0	0	0	0	0	0	1	0	0	0	0	0	0	0	0	0	0	0	0	0	0	0	0	0	0	0	0	0	0	0	0	0	0	0	0	0	0	0	0	0	0	0	0	5	0	0	0	0	0
Sporolactobac	0	0	0	0	0	0	0	2	0	0	0	0	0	0	0	0	0	0	0	0	0	0	0	0	0	0	0	0	0	0	0	0	0	0	0	0	0	0	0	0	0	33	0	0	0	0	0	0	0	0	0	0	0	0	0	0	0	0	0	0	0	0	0	0
Streptococcus	680	2880	16	1036	167	16	125	1716	31	246	12	43	84	5	11	110	131	319	5	16	332	32	0	2	28	34	18	169	177	225	123	15	6	14	31	9	1	1	116	57	0	263	3	197	14	1	5	28	1	0	0	1	0	595	115	0	53	303	31	120	33	14	540	654
Lactococcus	8	29	0	13	0	0	2	0	0	0	0	0	0	0	0	0	0	3	0	0	0	0	2	0	0	3	0	0	0	0	3	5	0	1	0	4	0	0	0	0	0	39	0	0	0	0	0	0	0	0	4	0	0	192	50	0	0	32	0	0	0	0	4	16
Granulicatella	101	681	0	123	0	15	11	28	71	0	22	0	0	0	0	20	0	61	0	0	89	19	0	0	13	26	23	25	26	125	88	11	15	8	0	0	0	0	0	19	0	113	1	289	1	0	0	2	0	0	0	0	0	142	6	0	0	48	0	35	13	0	115	48
Atopostipes	17	21	0	0	0	0	0	3	2	0	0	0	0	0	0	33	0	15	0	0	0	4	0	0	0	4	0	0	1	0	2	3	2	0	0	0	0	0	0	3	0	0	0	0	0	0	0	0	0	0	0	0	0	0	0	0	0	0	0	0	0	0	3	23
Desemzia	18	11	0	0	0	0	0	0	2	0	0	0	0	0	0	0	0	15	0	0	0	0	0	0	0	0	6	0	4	0	0	0	4	3	0	0	0	0	0	0	0	10	0	0	0	0	0	0	0	0	0	0	0	0	0	0	0	0	0	0	0	0	13	0
Trichococcus	10	7	0	22	0	0	0	0	2	0	0	0	0	0	0	5	0	0	0	0	0	0	0	0	0	0	0	0	0	0	0	0	0	0	0	0	0	0	0	0	0	68	0	0	0	0	0	0	0	0	0	0	0	0	0	152	0	0	0	4	0	0	0	1
Jeotgalibaca	1	0	0	0	0	0	0	0	0	0	0	0	0	0	0	0	0	3	0	0	0	0	0	0	0	0	0	0	0	0	0	0	0	0	0	0	0	0	0	1	0	0	0	0	0	0	0	0	0	0	0	0	0	0	0	0	0	0	0	0	0	0	0	0
Dolosigranulu	1	47	0	0	0	7	1	0	0	0	3	10	20	17	0	34	0	24	0	0	0	5	0	0	0	0	34	0	3	11	1	0	0	0	102	0	7	0	0	0	0	1	0	0	0	1	0	0	0	0	0	0	0	0	0	0	0	0	0	2	0	0	1	1
Marinilactibaci	0	30	0	40	0	0	0	0	0	0	0	0	0	0	0	0	0	5	0	0	0	0	0	0	0	0	0	0	0	0	0	0	0	0	0	0	0	0	0	0	0	0	0	0	0	0	0	0	0	0	0	0	0	0	0	0	0	0	0	0	0	0	4	25
Alloiococcus	0	0	0	0	0	0	0	0	0	0	1	0	0	0	0	1	0	0	0	0	0	0	0	0	0	1	0	8	5	0	0	0	0	0	0	0	0	0	0	0	0	0	0	0	0	0	0	0	0	0	0	0	0	0	0	0	0	0	0	0	0	0	0	0
Alkalibacteriu	0	0	0	0	0	0	0	0	0	0	0	0	0	0	0	29	0	0	0	0	0	0	0	0	0	0	2	0	3	0	0	0	0	0	0	0	0	0	0	0	0	3	0	0	0	0	0	0	0	0	0	0	0	0	0	0	0	0	0	8	0	3	0	0
Pisciglobus	0	0	0	0	0	0	0	0	0	0	0	0	0	0	0	0	0	2	0	0	0	0	0	0	0	0	0	0	0	0	0	0	0	0	0	0	0	0	0	0	0	0	0	0	0	0	0	0	0	0	0	0	0	0	0	0	0	0	0	2	0	0	0	0
Catellicoccus	0	0	0	0	0	0	0	0	0	0	0	0	0	0	0	0	0	8	0	0	0	0	0	0	0	0	0	0	0	0	0	0	0	0	0	0	0	0	0	0	0	0	0	0	0	0	0	0	0	0	0	0	0	0	0	0	0	0	0	0	0	0	0	0
Carnobacteriu	0	0	0	0	0	0	0	0	0	0	0	0	0	0	0	0	0	1	1	0	0	0	0	0	0	0	0	0	0	0	0	0	0	0	0	0	0	0	0	0	0	0	0	0	0	0	0	0	0	0	0	0	0	0	0	0	0	0	0	6	0	0	5	0
Atopococcus	0	0	0	0	0	0	0	0	0	0	0	0	0	0	0	0	0	0	0	0	0	0	0	0	0	2	0	0	0	0	0	0	0	2	0	0	0	0	0	0	0	0	0	0	0	0	0	0	0	0	0	0	0	0	0	0	0	0	0	0	0	0	1	0
Enterococcus	91	13	0	0	0	4	0	2	0	0	0	0	2	0	0	15	38	0	0	4	0	3	0	0	0	0	0	0	0	0	0	0	0	1	1	0	0	0	0	0	0	12	0	0	0	0	0	0	0	0	0	0	0	0	0	0	0	0	0	0	0	0	0	0
Vagococcus	2	0	0	0	0	2	0	0	0	0	0	0	0	0	0	0	0	0	0	0	0	0	0	0	0	0	0	0	0	0	0	0	0	0	0	0	0	0	0	0	0	12	0	0	0	0	0	0	0	0	0	0	0	0	28	0	11	0	0	0	0	0	0	0
Tetragenococc	0	13	0	0	0	0	0	0	0	0	0	0	0	0	0	0	0	0	0	0	0	0	0	0	0	0	0	0	0	0	0	0	0	0	0	0	0	0	0	0	0	0	0	0	0	0	0	0	0	0	0	0	0	0	0	0	0	0	0	0	0	0	0	8
Pilibacter	0	1	0	0	0	0	0	0	0	0	0	0	0	0	0	0	0	0	0	0	0	0	0	0	0	0	0	0	0	0	0	0	0	3	0	0	0	0	0	0	0	0	0	0	0	0	0	0	0	0	0	0	0	0	0	0	0	0	0	0	0	0	0	0
Aerococcus	157	97	11	266	0	0	18	43	8	9	2	20	0	3	0	10	0	19	0	0	13	0	0	4	0	7	8	16	37	0	13	5	0	2	0	2	0	8	386	7	0	542	1	80	6	6	9	12	0	106	13	6	1	433	44	17	1471	148	3	0	157	4	147	100
Facklamia	48	44	12	0	125	0	0	0	7	0	0	0	0	14	0	3	0	85	0	0	0	0	0	0	0	5	0	5	14	0	7	0	0	4	0	4	1	0	33	4	0	14	0	0	0	0	0	3	0	0	1	0	0	0	0	0	25	0	4	0	0	4	47	12
Eremococcus	130	268	0	51	0	0	2	0	0	0	0	0	0	0	0	0	0	52	0	0	0	0	0	0	14	0	8	13	0	10	9	1	19	0	0	0	0	0	1	0	0	0	0	0	0	0	0	0	0	0	0	0	0	101	0	0	103	0	6	0	0	0	120	0
Abiotrophia	24	165	0	0	0	20	12	14	13	0	0	0	0	0	0	0	0	5	0	0	0	0	0	0	0	0	21	0	0	0	0	0	0	0	10	0	0	0	0	0	0	0	0	50	0	0	0	0	0	0	0	0	0	93	0	0	0	0	0	31	0	0	0	84
Globicatella	0	0	0	0	0	4	0	0	4	0	0	0	0	0	0	0	0	0	0	0	1	0	0	0	0	0	0	0	0	0	0	0	0	0	0	5	0	0	0	0	0	0	0	0	0	0	0	0	0	0	0	0	0	0	0	0	0	0	0	0	0	0	0	0
Weissella	22	46	0	65	0	205	43	96	25	0	41	145	2653	53	205	2193	2899	487	4	35	2	61	3	1	43	6	59	317	24	21	8	16	1	20	0	91	18	0	14	0	0	16	1	13	0	0	4	2	0	0	0	0	0	0	614	0	0	43	4	22	0	1	22	3
Leuconostoc	15	19	0	77	0	8	4	5	1	0	0	0	0	1	0	12	0	6	0	0	2	0	0	0	0	1	2	0	1	0	0	0	0	0	0	0	3	0	0	2	0	25	0	1	0	0	1	1	0	0	0	3	0	0	0	0	116	0	0	0	0	0	34	9
Fructobacillus	1	0	0	0	0	0	0	0	0	0	0	0	0	0	0	0	0	0	0	0	0	0	0	0	0	0	0	0	0	0	0	0	0	0	0	0	0	0	0	0	0	0	0	0	0	0	0	0	0	0	0	0	0	0	0	0	0	0	0	0	0	0	0	0
Lactobacillus	40	45	0	85	157	4	0	1	4	2	1	5	91	0	6	11	476	5	1	0	334	0	2	0	0	0	147	26	1	3	0	0	1	0	3	0	0	0	10	2	0	41	0	0	0	5	0	0	0	0	0	0	1	272	72	0	0	0	1	1	25	7	43	8
Pediococcus	0	0	0	0	0	110	31	147	52	17	48	210	2068	24	201	893	557	285	10	40	16	56	18	3	28	1	90	720	67	53	4	11	1	13	0	94	21	0	0	0	0	0	0	0	0	0	0	0	0	0	0	0	0	0	0	0	0	0	0	0	0	0	0	0
Finegoldia	261	80	0	173	0	0	0	5	3	0	0	35	0	2	0	35	0	14	1	0	13	43	0	0	4	14	0	37	6	3	6	0	15	0	0	8	0	0	50	4	5	272	0	0	0	2	0	1	0	0	0	0	0	196	94	7	1	150	26	5	0	0	120	1789
Anaerococcus	263	1825	0	103	89	2	0	3	2	9	0	17	0	3	0	2	0	69	0	0	34	17	2	1	0	7	2	28	40	3	0	1	0	7	0	3	4	0	236	72	1	189	0	14	0	0	0	0	0	23	0	2	0	29	25	7	41	72	60	12	1	184	349	390
Gallicola	3	17	0	0	0	0	0	0	0	0	0	0	0	0	0	0	0	0	0	0	0	0	0	0	0	0	0	0	0	0	0	0	0	0	0	0	0	0	0	0	0	0	0	0	0	0	0	0	0	0	0	0	0	0	0	0	0	0	0	0	0	0	0	0
Murdochiella	1	0	0	0	73	0	0	0	0	0	0	0	0	0	0	0	0	0	0	0	14	0	0	0	2	0	0	0	0	0	0	0	2	0	0	0	0	0	0	5	0	0	0	0	0	0	0	0	0	1	0	0	0	0	0	0	0	0	0	0	0	0	9	0
Parvimonas	0	101	0	0	0	3	0	36	71	0	0	0	0	0	0	0	0	11	0	0	0	0	0	0	0	0	13	34	0	0	0	0	102	135	3	5	0	0	0	8	0	2	0	0	0	1	0	0	0	0	0	0	0	42	0	0	0	0	31	12	0	0	22	0
Clostridium_X	0	2	0	0	0	0	0	0	0	0	0	0	0	0	2	0	0	0	0	0	0	0	0	0	0	0	0	0	0	0	0	0	0	0	0	0	0	0	0	0	0	0	0	0	0	0	0	0	0	0	0	0	0	0	0	0	0	0	0	0	0	0	0	0
Ezakiella	0	0	0	4	0	2	0	0	0	0	0	8	0	0	0	0	0	0	0	0	0	0	0	0	0	0	0	1	0	0	0	0	0	0	0	0	0	42	2	0	0	20	0	0	0	0	0	0	0	0	0	0	0	0	27	0	0	93	82	0	0	0	0	11
Helcococcus	0	0	0	0	0	0	0	0	0	0	0	0	0	0	0	0	0	7	0	0	0	0	0	0	0	1	0	0	0	0	0	0	0	0	0	0	0	0	8	0	0	0	0	0	0	0	0	0	0	0	0	0	0	0	0	0	0	0	0	0	0	0	0	0
Sedimentibact	0	0	0	0	0	0	0	0	0	0	0	0	0	0	0	0	0	0	0	0	0	0	0	0	0	0	0	0	0	0	0	0	0	0	0	0	0	0	0	0	0	12	0	0	0	0	0	0	0	0	0	0	0	0	0	0	0	0	0	0	0	0	0	0
Romboutsia	61	12	0	30	0	3	0	313	188	0	0	15	0	0	0	388	453	88	5	0	24	43	0	0	0	17	11	40	117	27	21	8	3	49	0	9	0	0	39	14	0	8	0	24	0	0	0	0	0	0	0	0	0	47	0	0	75	0	152	0	0	7	119	321
Clostridium_X	1	0	0	0	0	0	0	0	0	0	0	0	0	0	0	0	0	0	0	0	0	0	0	0	0	0	0	0	0	0	0	0	0	0	0	0	0	0	0	0	0	4	0	0	0	0	0	0	0	0	0	0	0	0	0	0	7	0	0	0	0	0	0	0
Peptostreptoc	5	1	0	0	0	0	0	0	0	0	0	0	0	0	0	0	0	0	0	0	0	0	0	0	0	9	18	0	0	0	0	0	2	0	2	0	0	0	1	0	0	0	0	0	0	0	0	0	0	0	0	0	0	0	0	0	0	0	0	1	0	0	3	7
Filifactor	6	6	0	0	0	0	0	0	0	0	0	0	0	0	0	0	0	0	0	0	0	0	0	0	0	2	1	0	0	0	0	0	0	0	0	0	0	0	0	0	0	0	0	0	0	0	0	0	0	0	0	0	0	0	0	0	0	0	0	0	0	0	3	0
Asaccharospo	1	0	0	7	0	0	0	4	6	0	0	1	0	0	0	3	4	6	0	0	3	0	0	0	0	0	1	5	1	1	0	0	2	0	0	0	0	0	2	2	0	0	0	0	0	0	0	0	0	0	0	0	0	5	0	0	2	0	0	0	0	1	3	7
Intestinibacter	1	17	0	1	0	0	0	14	1	0	0	38	0	0	0	4	0	0	0	0	0	0	0	0	0	3	0	0	0	0	0	0	5	0	0	0	0	0	0	0	0	16	0	0	0	0	0	0	0	0	0	0	0	2	0	0	3	0	2	0	0	0	8	25
Terrisporobact	0	3	0	10	0	4	1	7	15	0	0	0	0	0	0	0	0	7	0	0	0	0	0	0	0	0	0	0	0	0	0	0	1	0	0	0	0	0	0	0	0	0	0	0	0	0	0	0	0	0	0	0	0	0	0	0	0	0	0	0	0	0	0	0
Peptostreptoc	0	2	0	0	0	0	0	0	0	0	0	0	0	0	0	0	0	0	0	0	0	0	0	0	0	0	0	0	0	0	0	0	0	0	0	0	0	0	0	0	0	0	0	0	0	0	0	0	0	0	0	0	0	0	0	0	0	0	0	0	0	0	0	0
Sporacetigeni	0	0	0	0	0	3	4	0	6	0	0	0	0	0	0	0	0	0	0	0	0	0	0	0	0	0	0	0	0	0	0	0	0	0	0	0	0	0	0	0	0	0	0	0	0	0	0	0	0	0	0	0	0	0	0	0	0	0	0	0	0	0	0	0
Tepidibacter	0	0	0	0	0	0	0	0	0	0	0	0	0	0	0	0	0	0	0	0	0	0	0	0	0	0	0	0	0	0	0	0	0	0	0	0	0	0	0	0	0	0	0	0	0	0	0	0	0	0	0	0	0	0	0	0	0	0	0	0	0	0	0	0
Fastidiosipila	9	0	0	0	0	0	0	0	0	0	0	0	0	0	0	0	0	0	0	0	0	0	0	0	0	0	0	0	0	0	0	0	0	0	0	0	0	0	0	0	0	0	0	0	0	0	0	0	0	0	0	0	0	0	0	0	0	0	0	0	0	0	0	0
Subdoligranul	1	0	0	0	0	0	0	0	0	0	0	0	0	0	0	0	0	0	0	0	0	0	0	0	0	0	0	3	1	0	0	0	0	0	0	0	0	0	0	0	0	0	0	0	0	0	0	0	0	0	0	0	0	0	0	0	0	1	0	0	0	0	0	6
Faecalibacter	0	6	0	0	0	0	0	0	24	0	0	0	0	0	0	0	0	0	0	1	0	0	0	21	0	0	105	217	0	0	9	0	123	0	0	0	0	0	5	0	0	0	0	0	0	0	0	0	0	0	0	0	0	112	0	0	0	0	39	0	0	0	0	325
Clostridium_IV	0	2	0	2	0	0	0	0	1	0	0	0	0	0	0	1	0	2	0	0	0	1	0	0	0	0	0	0	0	0	0	0	5	0	0	0	0	0	6	0	0	0	0	0	0	0	0	0	0	0	0	0	0	0	0	0	0	0	0	0	0	0	0	1
Anaerotruncus	0	3	0	0	0	0	0	0	0	0	0	0	0	0	0	0	0	0	0	0	0	0	0	0	0	0	0	0	0	0	0	0	0	0	0	0	0	0	0	0	0	0	0	0	0	0	0	0	0	0	0	0	0	0	0	0	0	0	0	0	0	0	0	0
Pseudoflavonifractor	0	0	0	0	0	3	0	0	0	0	0	0	0	0	0	0	0	0	0	0	0	0	0	0	0	0	0	0	0	0	0	0	0	0	0	0	0	0	0	0	0	0	0	0	0	0	0	0	0	0	0	0	0	0	0	0	0	0	0	0	0	0	0	0
Oscillibacter	0	0	0	0	0	0	0	2	0	0	0	0	0	0	1	0	0	4	0	0	0	0	0	0	0	0	0	1	0	0	0	0	0	0	0	0	0	0	1	0	0	4	0	0	0	0	0	0	0	0	0	0	0	0	0	0	0	0	0	0	0	11	16	0
Ruminococcus	0	0	0	0	0	0	0	2	0	0	0	0	0	0	0	0	0	0	0	0	0	0	0	1	0	0	1	1	0	0	0	0	0	0	0	0	0	0	0	0	0	0	0	0	0	0	0	0	0	0	0	0	0	0	0	0	0	0	0	0	0	0	0	14
Acetanaerobacterium	0	0	0	0	0	0	0	0	0	0	0	0	0	0	0	0	0	0	0	0	0	0	0	0	0	0	0	0	0	0	0	0	0	0	0	0	0	0	0	0	0	0	0	0	0	0	0	0	0	0	0	0	0	0	0	0	0	0	0	0	0	0	0	0
Butyricicoccus	0	0	0	0	0	0	0	0	0	0	0	0	0	0	0	0	0	0	0	1	0	0	0	0	0	2	0	0	0	0	0	0	1	0	0	0	0	0	0	0	0	0	0	0	0	0	0	0	0	0	0	0	0	0	0	0	0	0	0	0	0	0	0	0
Flavonifractor	0	0	0	0	0	0	0	0	0	0	0	0	0	0	0	0	0	0	0	3	0	0	0	0	0	0	0	0	0	0	0	0	0	0	0	0	0	0	0	0	0	0	0	0	0	0	0	0	0	0	0	0	0	0	0	0	0	0	0	0	0	0	0	0
Clostridium_III	0	0	0	0	0	0	0	0	0	0	0	0	0	0	0	0	0	0	0	0	0	0	0	0	0	0	0	0	0	0	0	0	0	0	0	0	0	0	4	0	0	0	0	0	0	0	0	0	0	0	0	0	0	21	0	0	0	0	0	0	0	0	0	0
Saccharofermentans	0	0	0	0	0	0	0	0	0	0	0	0	0	0	0	0	0	0	0	0	0	0	0	0	0	0	0	0	0	0	0	0	0	0	0	0	0	0	10	1	0	0	0	0	0	0	0	0	0	0	0	0	0	0	0	0	0	0	0	0	0	0	0	0
Anaerofilum	0	0	0	0	0	0	0	0	0	0	0	0	0	0	0	0	0	0	0	0	0	0	0	0	0	0	0	0	0	0	0	0	0	0	0	0	0	0	0	0	0	0	0	0	0	0	0	0	0	0	0	0	0	0	0	0	0	0	0	2	0	0	0	0
Gemmiger	0	0	0	0	0	0	0	0	0	0	0	0	0	0	0	0	0	0	0	0	0	0	0	0	0	0	0	0	0	0	0	0	0	0	0	0	0	0	0	0	0	0	0	0	0	0	0	0	0	0	0	0	0	0	0	0	0	0	0	0	0	0	0	2
Intestinimonas	0	0	0	0	0	0	0	0	0	0	0	0	0	0	0	0	0	0	0	0	0	0	0	0	0	0	0	0	0	0	0	0	0	0	0	0	0	0	0	0	0	0	0	0	0	0	0	0	0	0	0	0	0	0	0	0	0	0	0	0	0	0	0	1
Anaerobacterium	0	0	0	0	0	0	0	0	0	0	0	0	0	0	0	0	0	0	0	0	0	0	0	0	0	0	0	0	0	0	0	0	0	0	0	0	0	0	0	0	0	0	0	0	0	0	0	0	0	0	0	0	0	0	0	0	0	0	0	0	0	0	0	0
Hydrogenoanaerobacterium	0	0	0	0	0	0	0	0	0	0	0	0	0	0	0	0	0	0	0	0	0	0	0	0	0	0	0	0	0	0	0	0	0	0	0	0	0	0	0	0	0	0	0	0	0	0	0	0	0	0	0	0	0	0	0	0	0	0	0	0	0	0	0	0
Pseudoflavonifractor	0	0	0	0	0	0	0	0	0	0	0	0	0	0	0	0	0	0	0	0	0	0	0	0	0	0	0	0	0	0	0	0	0	0	0	0	0	0	0	0	0	0	0	0	0	0	0	0	0	0	0	0	0	0	0	0	0	0	0	0	0	0	0	0
Anaerobacter	3	4	0	0	0	2	1	3	0	0	0	0	0	0	0	3	0	1	0	0	0	0	0	0	0	0	0	0	0	0	0	0	0	0	0	0	0	0	0	0	0	2	0	0	0	0	0	0	0	0	0	0	0	1	0	0	0	0	0	0	0	0	8	2
Clostridium_sensu_stricto	14	18	0	5	0	6	2	0	10	4	0	0	1	0	0	17	0	9	1	0	9	0	0	0	0	0	0	0	0	0	0	0	1	1	0	0	0	0	9	1	0	55	0	0	0	0	0	0	0	0	0	0	0	16	0	0	4	20	19	0	0	0	16	16
Oceanirhabdus	0	0	0	0	2	0	0	0	0	0	0	0	0	0	0	0	0	0	0	0	0	0	0	0	0	0	0	0	0	0	0	0	0	0	0	0	0	0	0	0	0	0	0	0	0	0	0	0	0	0	0	0	0	0	0	0	0	0	0	0	0	0	0	0
Proteiniclasticum	0	0	0	0	0	0	0	0	0	0	0	0	0	0	0	0	0	0	0	0	0	0	0	0	0	0	0	0	0	0	0	0	0	4	0	0	0	0	0	0	0	14	0	0	0	0	0	0	0	0	0	0	0	0	0	0	0	0	0	0	0	0	1	0
Hathewaya	0	0	0	0	0	0	0	0	0	0	0	0	0	0	0	0	0	0	0	0	0	0	0	0	0	0	0	0	0	0	0	0	0	0	0	0	0	0	0	0	0	0	0	0	0	0	0	0	0	0	0	0	0	0	0	0	0	0	0	0	0	0	0	0
Fonticella	0	0	0	0	0	0	0	0	0	0	0	0	0	0	0	0	0	0	0	0	0	0	0	0	0	0	0	0	0	0	0	0	0	0	0	0	0	0	0	0	0	6	0	0	0	0	0	0	0	0	0	0	0	0	0	0	0	0	0	0	0	0	0	0
Youngiibacter	0	0	0	0	0	0	0	0	0	0	0	0	0	0	0	0	0	0	0	0	0	0	0	0	0	0	0	0	0	0	0	0	0	0	0	0	0	0	0	0	0	5	0	0	0	0	0	0	0	0	0	0	0	0	0	0	0	0	0	0	0	0	0	0
Peptoniphilus	94	45	2	77	0	0	0	1	0	0	0	0	0	0	2	5	2	0	0	8	3	0	0	0	0	0	3	2	2	0	0	0	0	0	0	0	0	0	19	4	0	41	0	0	0	0	0	0	0	0	0	0	0	64	54	0	164	49	1	0	0	4	71	69
Anaerosphaera	2	6	2	0	0	0	0	0	0	0	0	0	0	0	0	0	0	0	0	2	0	0	0	0	0	0	0	0	0	0	0	0	0	0	0	0	0	0	0	0	0	14	0	0	0	0	0	0	0	1	0	0	0	0	0	0	0	2	0	0	1	1	0	0
Mogibacterium	13	20	0	2	0	0	0	6	15	0	0	0	0	0	0	4	30	18	0	1	0	0	0	0	0	0	12	10	0	0	0	0	5	0	8	0	2	0	0	6	0	3	0	0	0	0	0	0	0	0	0	0	0	28	0	0	0	0	0	0	1	0	14	0
Anaerovorax	11	26	0	4	0	1	0	8	2	0	0	0	0	0	0	12	3	6	0	0	0	0	0	0	0	0	0	0	0	2	0	0	0	0	0	0	0	0	2	3	0	7	0	0	0	0	0	0	0	0	0	0	0	52	0	0	0	0	0	0	0	3	1	0
Catonella	6	0	0	0	0	0	0	0	0	0	0	0	0	0	0	0	54	6	0	1	0	0	0	0	0	0	0	6	0	0	0	0	6	0	0	0	0	0	0	0	0	12	0	0	0	0	0	0	0	0	0	0	0	1	0	0	0	0	0	0	0	0	0	0
Clostridium_XlVa	2	1	0	0	0	0	0	0	0	0	0	0	0	0	0	2	0	1	0	0	0	0	0	0	0	0	0	0	0	0	0	0	4	0	0	0	0	0	0	0	0	1	0	0	0	0	0	0	0	0	0	0	0	0	0	0	0	0	0	0	0	1	2	0
Lachnoanaerobaculum	2	9	0	0	0	0	0	8	0	0	2	0	0	0	12	10	0	3	0	9	0	0	0	0	0	3	0	7	2	0	4	0	1	0	0	0	0	0	0	0	0	0	0	1	0	0	0	0	0	0	0	0	0	0	0	0	0	0	0	0	0	0	3	0
Dorea	0	3	0	19	0	0	0	1	0	0	0	0	0	0	0	0	0	2	0	31	0	0	0	2	0	0	1	13	0	0	0	4	0	0	0	0	0	0	0	0	0	0	0	0	0	0	0	0	0	0	0	0	0	0	0	0	0	0	0	0	0	2	14	0
Lachnospiracea_incertae_sedis	0	2	0	0	0	0	0	3	0	0	0	0	0	0	0	1	0	3	0	17	0	0	0	0	0	0	14	10	0	0	0	0	4	24	0	0	0	0	3	0	0	0	0	0	0	0	0	0	0	0	0	0	0	13	0	0	0	0	0	0	0	2	12	0
Roseburia	0	4	0	0	47	0	0	2	1	0	0	0	0	0	0	0	0	0	0	0	0	0	0	0	0	0	8	0	0	0	0	4	0	0	0	0	0	1	0	0	0	0	0	0	0	0	0	0	0	0	0	0	0	0	0	0	0	0	0	0	0	0	22	0
Mobilitalea	0	1	0	0	0	0	0	0	0	0	0	0	0	0	0	0	0	0	0	0	0	0	0	0	1	0	0	0	0	0	0	0	0	0	1	0	0	0	2	0	0	0	0	0	0	0	0	0	0	0	0	0	0	0	0	0	0	0	0	0	0	0	0	0
Robinsoniella	0	1	0	0	0	0	0	0	0	0	0	0	0	0	0	0	0	0	0	1	0	0	0	0	0	0	1	0	1	0	0	0	0	0	0	0	0	0	0	0	0	0	0	0	0	0	0	0	0	0	0	0	0	0	0	0	0	0	0	0	0	0	0	0
Oribacterium	0	4	0	0	0	0	0	0	0	0	0	0	0	0	0	0	0	0	0	4	0	0	0	0	0	0	0	0	0	4	0	0	0	0	0	0	0	0	0	0	0	0	0	0	0	0	0	0	0	0	0	0	0	0	3	0	0	0	0	0	0	0	0	0
Blautia	0	3	0	0	0	0	0	0	0	0	1	0	0	0	0	0	0	1	0	20	0	0	0	0	0	0	2	3	0	0	0	0	0	0	0	0	0	0	0	0	0	0	0	0	0	0	0	0	0	0	0	0	0	5	0	0	0	0	0	0	0	0	3	48
Butyrivibrio	0	0	0	0	33	0	0	0	0	0	0	0	0	0	0	0	0	0	0	0	0	0	0	0	0	0	0	0	0	0	0	0	0	0	0	0	0	0	0	0	0	0	0	0	0	0	0	0	0	0	0	0	0	0	0	0	0	0	0	0	0	0	0	0
Cellulosilyticum	0	0	0	0	0	0	2	10	0	0	0	0	0	0	0	0	26	0	0	3	0	0	0	0	1	0	0	2	0	0	0	0	0	0	0	0	4	0	1	0	0	0	0	0	0	0	0	0	0	0	0	0	0	0	0	0	0	0	0	0	0	0	22	0
Anaerostipes	0	0	0	0	0	0	0	0	0	0	0	0	0	0	0	0	0	0	0	0	0	0	0	0	0	0	0	4	0	0	0	0	1	0	0	0	0	0	0	0	0	0	0	0	0	0	0	0	0	0	0	0	0	0	0	0	0	0	0	0	0	0	0	0
Stomatobaculum	0	0	0	0	0	0	0	0	0	0	1	0	0	0	0	0	0	3	0	7	0	0	0	0	0	0	0	1	0	10	0	0	3	0	0	0	0	0	0	0	0	0	0	0	0	0	0	0	0	0	0	0	0	0	0	0	0	0	0	0	0	0	4	0
Moryella	0	0	0	0	0	0	0	0	0	0	1	0	0	0	0	0	0	0	0	0	0	0	0	0	0	2	4	0	0	0	0	0	0	0	0	0	0	0	0	0	0	0	0	0	0	0	0	0	0	0	0	0	0	0	0	0	0	0	0	0	0	0	0	0
Clostridium_XIVb	0	0	0	0	0	0	0	0	0	0	0	0	0	0	0	14	0	0	0	0	0	0	0	0	0	0	0	0	0	0	0	0	0	0	0	0	0	0	0	0	0	0	0	0	0	0	0	0	0	0	0	0	0	2	0	0	0	0	0	0	0	0	1	0
Ruminococcus2	0	0	0	0	0	0	0	0	0	0	0	0	0	0	0	13	0	0	0	16	0	0	0	0	0	11	0	0	0	0	0	6	0	0	0	0	0	0	0	0	0	21	0	0	0	0	0	0	0	0	0	0	0	1	0	0	0	0	0	0	0	0	0	0
Fusicatenibacter	0	0	0	0	0	0	0	0	0	0	0	0	0	0	0	0	0	0	0	17	0	0	0	0	0	0	10	0	0	0	0	13	0	0	0	0	0	0	0	0	0	0	0	0	0	0	0	0	0	0	0	0	0	0	0	0	0	0	0	0	0	0	0	0
Lachnobacterium	0	0	0	0	0	0	0	0	0	0	0	0	0	0	0	0	0	0	0	0	0	0	0	0	1	0	0	0	0	0	0	0	0	0	0	0	0	0	0	0	0	0	0	0	0	0	0	0	0	0	0	0	0	0	0	0	0	0	0	0	0	0	0	0
Coprococcus	0	0	0	0	0	0	0	0	0	0	0	0	0	0	0	0	0	0	0	0	0	0	0	0	1	17	29	0	0	0	0	22	0	0	0	0	0	0	0	0	0	0	0	0	0	0	0	0	0	0	0	0	0	0	0	0	0	0	0	0	0	0	0	0
Shuttleworthia	0	0	0	0	0	0	0	0	0	0	0	0	0	0	0	0	0	0	0	0	0	0	0	0	0	0	1	0	0	0	0	0	0	0	0	0	0	0	0	0	0	1	0	0	0	0	0	0	0	0	0	0	0	0	0	0	0	0	0	0	0	0	0	0
Howardella	0	0	0	0	0	0	0	0	0	0	0	0	0	0	0	0	0	0	0	0	0	0	0	0	0	2	0	0	0	0	0	0	0	0	0	0	0	0	0	0	0	0	0	0	0	0	0	0	0	0	0	0	0	0	0	0	0	0	0	0	0	0	0	0
Anaerosporobacter	0	0	0	0	0	0	0	0	0	0	0	0	0	0	0	0	0	0	0	0	0	0	0	0	0	0	0	0	0	0	0	0	0	0	0	0	0	0	0	0	0	5	0	0	0	0	0	0	0	0	0	0	0	0	0	0	0	0	0	0	0	0	0	0
Acetatifactor	0	0	0	0	0	0	0	0	0	0	0	0	0	0	0	0	0	0	0	0	0	0	0	0	0	0	0	0	0	0	0	0	0	0	0	0	0	0	0	0	0	0	0	0	0	0	0	0	0	0	0	0	0	0	0	0	0	0	0	0	0	0	0	0
Syntrophococcus	0	0	0	0	0	0	0	0	0	0	0	0	0	0	0	0	0	0	0	0	0	0	0	0	0	0	0	0	0	0	0	0	0	0	0	0	0	0	0	0	0	0	0	0	1	0	0	0	0	0	0	0	0	0	0	0	0	0	0	0	0	0	1	0
Eubacterium	0	19	0	0	0	0	0	0	0	0	0	0	0	0	0	0	0	0	0	0	0	0	0	0	0	5	0	0	0	0	0	0	0	0	0	0	0	0	0	0	0	0	0	0	0	0	0	0	0	0	0	0	0	0	0	0	0	0	0	0	0	0	7	0
Anaerofustis	0	0	0	0	0	2	0	0	0	0	0	0	0	0	0	0	0	0	0	0	0	0	0	0	0	0	0	0	0	0	0	0	0	0	0	0	0	0	0	0	0	0	0	0	0	0	0	0	0	0	0	0	0	0	0	0	0	0	0	0	0	0	0	0
Pseudoramibacter	0	0	0	0	0	5	0	0	0	0	0	0	0	0	0	0	0	0	0	0	0	0	0	0	0	0	0	0	0	0	0	0	6	0	0	0	0	0	0	0	0	0	0	0	0	0	0	0	0	0	0	0	0	0	0	0	0	0	0	0	0	0	0	0
Peptococcus	0	1	0	0	0	0	0	0	0	0	0	0	0	0	0	0	0	0	0	0	0	0	0	0	0	0	0	0	0	0	0	0	0	0	0	0	0	0	0	0	0	0	0	0	0	0	0	0	0	0	0	0	0	0	0	0	0	0	0	0	0	0	0	0
Desulfosporosinus	0	0	0	0	0	0	0	0	0	0	0	0	0	0	0	0	0	0	0	0	0	0	0	0	0	0	0	0	0	0	0	0	0	0	0	0	0	0	0	0	0	0	0	0	0	0	0	0	0	0	0	0	0	0	0	0	0	0	0	0	0	0	3	0
Geosporobacter	0	0	0	0	0	0	0	0	0	0	0	0	0	0	0	0	0	0	0	0	0	0	0	0	0	0	0	0	0	0	0	0	0	0	0	0	0	0	0	0	0	0	0	0	0	0	0	0	0	0	0	0	0	0	0	0	0	0	0	0	0	0	0	0
Anaerosolibacter	0	0	0	0	0	0	0	0	0	0	0	0	0	0	0	0	0	0	0	0	0	0	0	0	0	0	0	0	0	0	0	0	0	0	0	0	0	0	0	0	0	6	0	0	0	0	0	0	0	0	0	0	0	0	0	0	0	0	0	0	0	0	0	0
Brassicibacter	0	0	0	0	0	0	0	1	0	0	0	0	0	0	0	0	0	0	0	0	0	0	0	0	0	0	0	0	0	0	0	0	0	0	0	0	0	0	0	0	0	0	0	0	0	0	0	0	0	0	0	0	0	0	0	0	0	0	0	0	0	0	0	0
Proteinivorax	1	0	0	0	0	0	0	0	0	0	0	0	0	0	0	0	0	0	0	0	0	0	0	0	0	0	0	0	0	0	0	0	0	0	0	0	0	0	0	0	0	0	0	0	0	0	0	0	0	0	0	0	0	0	0	0	0	0	0	0	0	0	0	0
unclassified_B	15891	16526	1484	8497	2519	11579	9694	11931	7042	5922	4761	8075	9340	3320	5501	7968	8476	11502	318	4283	9724	6072	2855	4596	4841	7319	7663	9565	11198	7319	8915	10229	2812	11362	5240	6253	4434	5357	9433	5652	5081	9997	7053	6038	2628	3316	4969	7224	2052	3613	5312	6464	6875	10535	9949	10257	13295	10423	4515	3353	8745	9152	18018	17150

F8_S14	F9_S22	G10_S31	G7_S7	G8_S15	G9_S23	H10_S32	H7_S8	H8_S16	H9_S24	R10	R11	R12	R13	R14	R15	R1	R2	R3	R4	R5	R6	R7	R8	R9
826	17	48	286	21	206	11	95	66	55	3	24	23	13	7	0	1	23	12	5	17	11	0	16	3
1	1	0	41	0	1	0	0	8	13	0	0	1	4	2	0	0	1	2	0	2	1	1	0	0
0	9	0	0	0	38	0	0	65	0	0	10	0	0	0	0	0	8	0	3	31	0	0	0	0
0	0	0	0	0	0	0	0	0	0	0	0	0	0	0	0	8	0	114	0	10	0	0	0	0
88	63	618	261	162	859	193	525	3009	1562	167	91	220	236	60	10	22	353	429	24	326	11	19	671	326
45	84	598	3268	246	3049	191	659	2455	424	9	343	76	83	27	23	31	1530	182	30	606	47	119	1201	63
0	10	27	0	0	0	0	132	46	42	2	17	1	10	0	2	22	1	69	67	0	29	4	779	23
0	0	0	0	0	0	0	2	2	0	1	0	0	0	0	0	0	0	0	0	0	0	0	0	0
4	1	0	30	2	3	4	33	35	6	0	0	0	18	1	0	0	8	66	3	11	5	0	35	3
49	0	114	0	6	178	1	0	0	0	0	0	2	0	0	2	7	0	0	0	0	0	2	0	0
0	0	0	0	0	0	0	0	0	0	0	0	0	0	0	0	0	0	0	0	0	0	0	0	0
0	0	0	4	0	0	0	0	0	0	0	0	0	0	0	0	0	0	0	0	0	0	0	0	0
0	0	0	0	0	0	0	0	0	0	0	0	0	0	0	0	0	0	0	0	0	0	0	0	0
0	0	0	0	0	0	0	0	0	0	0	0	0	0	0	0	0	0	0	0	0	0	0	0	0
0	0	0	0	0	0	0	0	0	0	0	0	0	0	0	0	0	0	0	0	0	0	0	0	0
0	0	0	0	0	0	0	0	0	0	0	0	0	0	0	0	0	0	0	0	0	0	0	0	0
0	0	0	0	0	0	0	0	0	0	0	0	0	1	0	0	0	0	0	0	0	0	0	0	0
0	0	0	0	27	0	0	53	0	0	0	0	0	2	0	0	1	9	0	0	0	5	0	17	8
40	9	3	148	20	155	23	131	3	142	7	0	10	5	41	4	391	9	0	0	0	4	2	0	0
0	0	0	0	40	0	0	55	70	0	0	0	0	0	0	0	0	16	41	0	0	5	0	0	0
0	0	0	0	0	0	0	0	0	0	0	0	0	0	0	0	0	0	0	0	0	0	0	0	0
0	0	0	0	0	0	0	2	0	0	0	0	0	0	0	0	0	0	0	0	0	0	0	0	0
0	0	0	0	1	0	0	0	0	0	0	0	0	0	0	0	0	0	0	0	0	0	0	0	0
0	0	0	0	0	0	0	0	0	0	0	0	0	0	0	0	0	0	0	0	0	0	0	0	0
13	5	69	9	344	59	16	94	65	25	1	3	20	10	0	1	0	16	13	0	25	8	0	21	0
0	0	0	9	0	0	0	14	0	0	0	0	0	0	0	0	0	8	0	0	0	0	0	0	17
2	2	9	0	29	0	0	125	34	0	0	3	5	0	0	0	0	7	0	0	5	0	0	0	0
0	0	0	0	0	0	0	0	0	0	0	0	0	0	0	0	0	0	0	0	0	0	0	0	0
0	0	0	15	6	0	0	0	0	0	0	1	0	0	0	0	0	0	0	0	0	0	0	0	0
0	0	0	0	0	0	0	0	0	0	0	0	0	0	0	0	0	0	0	0	0	0	0	0	0
0	0	0	0	0	0	0	0	0	0	0	0	0	0	5	2	0	83	0	0	0	0	0	0	0
0	0	0	0	0	0	0	0	0	0	0	0	0	0	0	0	0	1	0	0	0	0	0	0	0
0	0	0	1	0	0	0	43	5	0	0	0	0	0	0	0	0	0	0	0	0	0	0	0	0
0	0	0	11	0	0	0	0	0	0	0	0	0	0	0	0	0	0	0	0	0	0	0	0	0
0	0	0	0	0	0	0	0	0	0	0	0	0	0	0	0	0	0	0	0	0	0	0	0	0
0	0	0	0	0	0	0	0	0	0	0	0	0	0	0	0	0	0	0	0	0	0	0	0	0
0	0	0	0	0	0	0	0	25	0	0	0	2	0	0	0	0	0	0	0	0	0	0	0	0
0	0	0	0	0	0	0	4	0	0	0	0	0	0	0	0	0	0	0	0	0	0	0	0	0
0	0	0	0	0	0	0	0	0	0	0	0	0	0	0	0	0	0	0	15	0	0	0	0	0
0	0	0	0	0	0	0	0	0	0	0	0	0	0	0	0	0	0	0	0	0	0	0	0	0
0	0	0	0	0	0	0	0	0	0	0	0	0	0	0	0	0	0	0	0	0	0	0	0	0
0	0	0	0	0	0	0	0	0	0	0	0	0	0	0	0	0	0	3	0	0	0	0	0	0
0	0	0	1	0	0	0	0	0	0	0	0	0	0	0	0	0	0	0	0	0	0	0	0	0
8	9	12	76	0	23	10	122	89	0	0	0	0	11	0	0	0	19	0	0	0	0	0	0	0
74	22	35	14	21	42	1	142	219	26	0	5	4	8	39	3	6	11	32	181	44	11	2	0	6
2	18	17	123	25	68	108	234	443	82	1	21	24	17	11	0	3	30	0	0	10	0	1	0	3
0	0	0	0	0	0	0	0	16	0	0	0	251	0	3	0	0	0	0	0	0	0	1	0	0
1	11	0	0	22	0	0	75	2	0	0	40	0	0	3	0	1	0	0	0	0	0	0	0	0
0	0	1	0	8	28	0	1098	13	22	0	0	2	0	0	0	0	0	0	0	0	1	0	0	0
0	0	0	0	0	0	0	7	0	0	0	0	0	0	0	0	0	0	0	0	0	0	0	0	0
0	0	0	0	0	0	0	1	9	0	0	0	0	0	0	0	0	0	0	0	0	0	0	0	0
0	0	0	0	0	0	0	0	0	0	0	0	0	0	0	0	0	0	0	0	0	0	0	0	0
0	0	8	0	0	0	0	0	0	0	0	0	0	0	0	0	0	0	0	0	0	0	0	0	0
0	0	0	0	0	0	0	0	0	0	0	0	0	0	0	0	0	0	0	0	0	0	0	0	0
0	0	0	0	0	0	0	0	11	0	0	0	0	0	0	0	0	0	0	0	0	0	0	0	0
0	0	11	0	0	0	0	24	0	0	0	0	0	0	0	0	0	0	0	0	0	0	0	0	0
0	0	0	0	0	0	0	0	0	0	0	0	0	0	0	0	0	0	0	0	0	0	0	0	0
0	0	0	0	0	0	0	0	0	0	0	0	0	0	0	0	0	0	0	0	0	0	0	0	0
0	0	0	0	0	0	0	0	0	0	0	0	0	0	0	0	0	0	0	0	0	0	0	0	0
0	0	514	0	23	0	11	233	0	0	0	0	0	0	0	0	0	0	0	0	0	7	0	0	0
0	0	0	0	0	18	0	2	5	0	0	0	0	0	0	0	0	0	0	0	0	0	0	0	0
18	16	0	37	14	393	10	41	71	0	0	20	5	6	91	7	14	9	69	57	19	7	1	3	60
0	1	0	0	0	0	18	0	22	0	0	5	4	8	0	0	0	43	0	0	0	0	2	18	0
0	0	0	0	0	0	0	0	0	0	0	0	0	0	0	0	0	0	0	0	0	0	0	0	0
5	0	0	0	0	0	0	0	0	0	0	0	0	0	0	0	0	0	0	0	0	0	0	0	0
0	0	0	0	0	0	0	1	0	0	0	0	0	0	0	0	0	0	0	0	0	0	0	0	0
0	0	0	0	0	0	0	0	0	0	0	0	0	0	0	0	0	2	0	0	0	0	0	0	0
0	3	0	0	0	56	17	3	16	0	0	0	0	13	0	0	0	0	0	0	0	0	0	0	3
0	0	0	0	0	0	3	0	8	0	0	1	0	0	0	0	0	0	0	0	0	0	0	0	0
0	0	0	0	0	1	0	1	0	0	0	0	0	0	0	0	0	0	0	0	0	0	0	0	0
0	0	0	0	0	0	0	0	0	0	0	0	0	0	0	0	0	0	0	0	0	0	0	0	0
0	0	0	0	0	0	0	0	0	0	0	0	0	0	0	0	0	0	0	0	0	0	0	0	0
0	0	0	14	0	34	0	0	0	0	0	0	0	0	4	0	0	3	0	0	0	0	0	0	0
0	0	0	0	0	0	0	0	0	0	0	0	0	0	0	0	0	4	0	0	0	0	0	0	0
0	0	0	0	0	0	0	0	0	0	0	0	0	0	0	0	0	0	0	0	0	0	0	0	0
0	0	0	0	0	0	10	2	31	0	0	3	0	0	0	0	0	0	0	0	0	0	0	0	0
0	0	0	0	0	0	0	0	0	0	0	0	2	0	1	0	0	62	0	0	0	0	0	0	16
0	0	0	0	0	0	0	0	0	0	0	0	0	0	0	0	0	0	0	0	0	0	0	0	0
31	76545	12524	104	185	6298	76828	213	140	2718	5145	13709	83	18471	21873	36	19506	352	10026	18577	3554	1240	14	76	21983
0	1	16	0	20	9	32	263	24	7	0	0	3	14	0	2	0	0	0	0	0	0	0	6	0
0	0	0	0	0	0	0	0	24	0	0	0	0	0	0	0	0	0	0	0	0	0	0	0	0
0	0	0	0	0	0	0	0	9	45	0	7	1	0	0	0	0	0	0	0	0	0	0	0	0
3	0	23	0	0	0	0	322	0	0	0	0	44	0	1	0	0	0	0	0	0	0	0	0	0
0	1	22	0	0	25	2	121	6	1	0	0	3	0	0	0	0	0	0	0	11	0	0	0	0
0	0	0	0	0	0	0	1	0	0	0	0	6	0	0	0	0	0	0	0	0	0	0	0	2
0	0	0	0	0	0	0	0	0	14	0	0	0	0	0	0	0	1	0	0	0	0	0	0	0
0	0	0	0	0	0	0	0	0	0	0	0	0	0	0	0	0	0	0	0	0	0	0	0	0
0	2	0	0	23	0	0	0	5	0	0	0	2	1	0	5	0	7	0	0	0	0	0	0	0
0	0	0	0	0	0	0	31	0	0	1	0	0	0	0	0	0	0	0	0	0	0	0	0	0
0	0	0	0	0	0	0	0	0	0	0	0	0	0	22	1	0	0	0	0	0	0	0	0	0
0	0	0	0	0	0	0	0	0	0	0	0	0	0	0	0	0	0	0	0	0	0	0	0	0
0	0	0	0	0	0	0	0	0	0	0	0	0	0	0	0	0	0	0	0	0	0	0	0	0
0	0	0	0	0	0	0	0	0	0	0	0	0	0	0	0	0	0	0	21	0	0	0	0	0
7	9	221	19	293	97	26	197	131	196	0	11	7	19	5	1	0	7	2	6	22	1	0	3	8
0	0	0	0	15	0	2	0	1	0	0	0	1	0	0	0	1	0	0	0	0	0	1	0	0
0	4	0	108	1	106	21	113	93	11	1	30	22	10	1	1	3	8	0	1	1	1	0	10	0
0	0	0	0	3	0	0	0	1	10	0	3	0	0	0	0	0	0	0	0	0	0	0	0	0
0	0	0	0	0	0	0	0	0	0	0	0	0	0	0	0	0	0	0	0	0	0	0	0	0
285	1333	2631	6844	685	1705	326	9407	7982	3477	984	1817	22081	2766	159	187	548	729	6411	1089	1946	880	29629	3120	2478
0	0	0	0	0	0	26	1	0	0	0	0	0	0	0	0	0	0	0	0	0	0	0	0	0
2	23	78	94	4	107	28	295	125	75	1	35	3	2	2	2	40	2	0	6	23	0	0	0	7
0	35	0	0	0	8	0	590	72	7	3	37	2	0	2	2	0	4	0	42	0	0	0	0	0
3	0	18	0	3	0	1	20	66	15	0	0	0	3	0	0	0	0	36	0	4	0	0	0	0
0	0	0	0	0	0	0	25	9	0	0	0	0	0	0	3	0	0	0	0	0	0	0	0	31
0	0	0	0	0	0	0	0	9	0	0	1	0	0	0	0	0	0	0	0	0	1	0	0	0
0	0	0	0	0	0	0	0	2	0	0	0	0	0	0	0	0	0	0	0	0	0	0	0	0
0	0	0	0	0	0	0	1	26	0	0	0	0	0	2	0	0	0	0	0	0	0	0	0	0
66	6	179	88	131	50	34	33	90	27	1	10	6	0	0	1	0	21	0	2	0	0	0	2	0
0	1	0	0	0	0	0	0	22	0	0	0	0	0	0	0	0	0	0	0	0	0	0	0	0
0	0	0	0	0	1	0	1	2	0	0	0	0	0	0	0	0	0	0	0	0	0	0	0	0
0	17	350	88	21	36	20	167	336	22	0	44	51	19	0	0	1	4	0	3	5	2	1	6	0
0	17	0	0	0	97	0	71	22	0	0	7	0	0	1	0	1	0	0	1	1	0	0	8	0
0	0	0	0	0	0	0	0	0	0	0	0	0	0	0	0	0	0	0	0	0	0	0	0	0
0	0	0	0	0	0	0	0	0	0	0	0	0	0	0	0	0	0	0	0	0	0	0	0	0
0	0	0	0	0	0	0	1	0	0	0	0	0	0	0	0	0	0	0	0	0	0	0	0	0
0	0	0	0	0	0	0	0	0	0	0	0	0	0	0	0	0	0	0	0	0	0	0	0	0
0	0	0	0	0	0	0	0	5	0	0	0	0	0	0	0	0	0	0	0	0	0	0	0	0
0	0	0	0	0	0	0	0	0	0	0	0	0	0	0	0	0	0	0	0	0	0	0	0	0
0	0	47	0	0	38	0	0	0	8	0	0	0	0	0	0	0	0	0	0	0	0	0	0	0
2	14	27	17	4	0	5	37	70	12	0	0	9	1	0	1	1	0	1	0	8	0	0	0	0
0	2	0	0	13	49	0	9	27	63	0	0	0	0	0	0	0	0	0	0	0	0	0	0	0
0	0	0	0	0	0	0	0	0	0	0	0	0	0	0	0	0	0	0	0	0	0	0	0	0
0	0	0	0	0	0	0	0	0	0	0	0	0	0	0	0	0	0	0	0	0	0	0	0	0
0	0	0	0	0	0	0	0	0	0	0	0	0	0	0	0	0	0	0	0	0	0	0	0	0
0	0	0	0	0	0	0	0	0	0	0	0	0	0	0	0	0	0	0	0	0	0	0	0	0
0	0	0	0	0	0	0	19	4	0	0	0	0	0	0	0	0	0	0	0	0	0	0	0	0
4	1	108	13	0	60	0	19	32	43	0	0	0	0	0	0	0	0	0	0	0	0	0	0	0
0	0	0	0	0	0	0	26	18	3	0	0	0	0	0	0	0	0	0	0	0	0	0	0	10
0	0	46	0	0	229	3	0	66	0	0	0	0	0	0	0	0	0	0	0	0	0	0	0	0
1	0	0	6	0	38	6	19	31	20	0	0	1	0	1	0	0	0	0	0	3	2	0	0	20
0	0	0	0	0	0	0	0	0	0	0	0	0	0	0	0	0	0	0	0	0	0	0	0	0
0	0	0	0	0	0	0	0	2	0	0	0	0	0	0	0	0	0	0	0	0	0	0	0	0
0	0	0	0	0	0	0	0	0	33	0	0	0	0	0	0	0	0	0	0	0	0	0	0	0
0	0	0	0	0	0	0	0	0	0	0	0	0	0	0	0	0	0	0	0	0	0	0	0	0
0	0	0	0	0	0	0	0	0	0	0	0	0	0	0	0	0	0	0	0	0	0	0	0	0
0	0	0	0	0	0	0	0	0	0	0	0	0	0	0	0	0	0	0	0	0	0	0	0	0
0	0	0	0	0	0	0	0	0	0	0	0	0	0	0	0	0	0	0	0	0	0	0	0	0
0	0	0	0	0	0	0	0	5	0	0	0	0	0	0	0	0	0	0	0	0	0	0	0	0
0	0	0	0	0	0	0	0	0	0	0	0	0	0	0	0	0	0	0	0	0	0	0	0	0
0	0	0	0	0	0	0	0	11	0	0	0	0	0	0	0	0	0	0	0	0	0	0	0	0
0	0	0	0	6	0	0	0	0	0	0	0	0	0	0	0	0	0	0	0	0	0	0	0	0
0	0	0	0	0	0	0	1	0	0	0	0	0	0	0	0	0	0	0	0	0	0	0	0	0
0	0	0	0	0	43	0	0	0	0	0	0	0	0	0	0	0	0	0	0	0	0	0	0	0
0	0	0	2	0	0	0	2	0	0	0	0	4	0	0	0	0	0	0	0	0	0	0	0	0
0	0	0	0	0	0	0	17	0	0	0	0	0	0	0	0	0	0	0	0	0	0	0	0	0
0	1	0	0	0	0	0	0	0	0	0	0	0	0	0	0	0	0	0	0	0	0	0	0	0
0	0	0	0	0	0	1	0	17	0	0	0	0	0	0	0	0	0	0	0	0	0	0	0	0
0	0	0	0	0	0	0	0	0	0	0	0	0	0	0	0	0	0	0	0	0	0	0	0	0
0	0	0	0	0	0	0	0	0	0	0	0	0	0	0	0	0	0	0	0	0	0	0	0	0
0	0	0	0	0	0	0	0	0	0	0	0	0	0	0	0	0	0	0	0	0	0	0	0	0
0	0	0	0	0	0	0	0	0	0	0	0	0	0	0	0	0	0	0	0	0	0	0	0	0
0	0	0	0	0	0	0	0	0	0	0	0	0	0	0	0	0	0	0	0	0	0	0	0	0
0	0	0	0	0	0	0	0	0	0	0	0	0	0	0	0	0	0	0	0	0	0	0	0	0
0	0	0	0	0	0	0	0	0	0	0	0	0	0	0	0	0	0	0	0	0	0	0	0	0
0	0	0	4	0	0	6	0	0	0	0	0	0	0	0	0	1	0	0	0	0	0	0	0	0
0	0	0	0	0	0	0	0	0	0	0	0	0	0	0	0	0	0	0	0	0	0	0	0	0
0	1	0	5	0	41	0	4	33	21	0	0	0	0	0	0	1	0	0	0	0	0	0	0	0
0	0	0	0	0	0	0	0	0	0	0	0	0	0	0	0	0	0	0	0	0	0	0	0	0
0	0	0	0	0	3	0	0	0	0	0	0	0	0	0	0	0	0	0	0	0	0	0	0	0
0	1	0	0	0	3	0	39	22	26	0	0	0	0	0	0	0	0	0	0	0	0	0	0	0
0	0	0	0	0	0	0	0	0	0	0	0	0	0	0	0	0	0	0	0	0	0	0	0	0
0	0	0	0	0	0	0	0	0	0	0	0	0	0	0	0	0	0	0	0	0	0	0	0	0
0	0	0	0	0	0	0	0	0	0	0	0	0	0	0	0	0	0	0	0	0	0	0	0	0
0	0	0	0	0	0	0	0	0	0	0	0	3	0	0	0	0	0	0	0	0	0	0	0	0
0	0	0	0	0	0	0	8	0	0	0	0	0	0	0	0	0	0	0	0	0	0	0	0	0
0	0	0	0	0	0	0	0	0	0	0	0	0	0	0	0	0	0	0	0	0	0	0	0	0
0	0	0	0	0	0	0	0	0	0	0	0	0	0	0	0	0	0	0	0	0	0	0	0	0
0	0	0	0	0	0	0	2	0	0	0	0	0	0	0	0	0	0	0	0	0	0	0	0	0
0	0	0	0	0	0	0	3	0	0	0	0	0	0	0	0	0	0	0	0	0	0	0	0	0
0	0	0	0	0	0	0	0	0	0	0	0	4	0	0	0	0	0	0	0	0	0	0	0	0
0	0	0	0	0	0	0	0	0	0	0	0	0	0	0	0	0	3	0	0	0	0	0	0	0
0	2	0	0	0	0	0	0	0	0	0	0	0	0	0	0	0	0	0	0	0	0	0	0	0
0	0	0	0	0	0	0	24	5	12	0	0	0	0	0	0	0	1	0	0	0	0	0	0	0
0	0	0	0	0	0	0	0	0	0	0	0	0	0	0	0	0	0	0	0	0	0	0	0	0
0	0	0	0	0	0	0	0	0	0	0	0	0	0	0	0	0	0	0	0	0	0	0	0	0
0	0	0	68	0	0	0	0	0	0	0	0	0	0	0	0	0	0	0	0	0	0	0	0	0
0	0	0	0	0	0	0	0	6	0	0	0	0	0	0	0	0	0	0	0	0	0	0	0	0
0	0	0	0	0	0	0	0	0	0	0	0	0	0	0	0	0	0	0	0	0	0	0	0	0
0	0	0	0	0	0	0	0	0	0	0	0	0	0	0	0	0	0	0	0	0	0	1	1	0
0	0	0	0	0	0	0	0	0	0	0	0	0	0	0	0	0	0	0	0	0	0	0	0	0
0	0	0	0	0	0	0	0	0	0	0	0	0	0	0	0	1	0	0	0	0	0	0	0	0
0	0	0	0	0	0	0	0	0	0	0	0	0	0	0	0	0	0	0	0	0	0	0	0	0
0	6	0	24	0	0	11	4	0	0	0	5	0	0	1	0	0	1	0	0	0	0	0	0	0
0	0	0	0	0	1	1	1	3	1	0	0	0	0	0	0	0	1	0	0	0	0	0	0	0
0	4	0	0	0	0	0	20	15	0	0	0	0	0	0	0	3	0	0	0	0	0	1	0	0
0	0	0	0	0	2	0	0	2	0	0	0	0	1	0	0	0	0	0	0	0	0	0	0	0
1	3	34	32	4	21	0	20	23	0	0	0	2	10	2	0	0	0	0	0	0	0	0	0	0
0	0	0	0	0	9	0	9	0	0	0	0	2	0	0	0	3	0	0	0	0	1	0	0	0
0	0	0	0	0	0	0	2	0	6	0	3	4	0	0	0	0	8	0	0	0	0	0	0	0
0	0	0	0	0	13	0	13	0	23	0	0	0	0	0	0	0	0	0	0	0	0	0	0	0
0	0	0	0	0	0	0	0	1	0	0	0	0	0	0	0	0	0	0	0	0	0	0	0	0
0	0	0	0	0	0	1	0	1	0	0	0	0	0	0	0	0	0	0	0	0	0	0	0	0
0	0	0	12	0	0	10	2	2	6	0	0	0	0	0	0	0	0	0	0	0	0	0	0	0
0	0	0	0	0	0	0	0	37	0	0	0	0	0	0	0	0	0	0	0	0	0	0	0	0
0	0	21	7	0	0	0	0	4	0	0	2	0	0	0	0	0	0	0	0	0	0	0	0	0
0	0	0	0	0	0	0	0	20	0	0	0	0	0	0	0	0	0	0	0	0	0	0	0	0
0	0	0	0	0	0	0	0	0	0	0	0	0	0	0	0	0	0	0	0	0	0	0	0	0
0	0	0	0	0	0	16	0	13	0	0	0	0	0	0	0	0	0	0	0	0	0	0	0	0
0	0	0	0	0	0	0	0	0	0	0	0	0	0	0	0	0	0	0	0	0	0	0	0	0
0	0	0	0	0	0	25	6	0	0	0	0	0	0	0	0	0	0	0	3	0	0	0	0	0
0	0	0	10	0	0	0	0	5	0	0	0	0	0	0	0	0	0	0	0	0	0	0	0	0
0	0	0	0	0	1	0	0	0	0	0	0	0	0	0	0	0	0	0	0	0	0	0	0	0
0	0	0	0	0	0	17	0	1	0	0	79	2	0	0	0	0	23	0	0	0	0	0	0	16
0	2	0	0	0	0	0	0	0	0	0	0	0	0	0	0	0	0	0	0	0	0	0	0	0
0	0	0	0	0	0	0	1	3	0	0	0	0	0	0	0	0	0	0	0	18	0	0	0	0
0	0	18	0	4	0	0	95	0	0	0	0	0	0	0	0	0	0	0	0	0	0	0	0	0
0	0	0	0	0	0	0	0	0	0	0	0	0	0	0	0	0	0	0	0	0	0	0	0	0
0	0	0	117	0	0	0	0	0	0	0	0	0	0	0	0	0	0	1	0	0	0	0	0	0
0	0	0	0	0	0	0	0	13	0	0	0	0	0	0	0	0	0	0	0	0	0	0	0	0
0	0	0	0	0	0	0	0	0	0	0	0	0	0	0	0	0	0	0	0	0	0	0	0	0
0	0	0	0	0	0	0	0	0	0	0	0	0	0	0	0	0	0	0	0	0	0	0	0	0
0	0	0	0	0	0	0	0	0	0	0	0	0	0	0	0	0	0	0	0	0	0	0	0	0
0	0	0	0	0	0	0	0	0	0	0	0	0	0	0	0	0	0	0	0	0	0	0	0	0
0	0	0	0	0	0	0	0	509	0	0	0	0	0	0	0	0	0	0	0	0	0	0	0	0
0	0	0	0	0	0	0	0	3	0	0	0	0	0	0	0	0	0	0	0	0	0	0	0	0
7	0	0	0	0	0	0	17	0	0	0	0	0	0	0	0	0	0	0	0	0	0	0	0	0
0	0	0	0	0	1	0	0	3	0	0	0	0	0	0	0	0	0	0	0	0	0	0	0	0
0	0	0	0	0	0	0	0	0	0	0	0	0	0	0	0	0	0	0	0	0	0	0	0	0
0	0	0	0	0	0	0	0	0	0	0	0	0	0	0	0	0	0	0	0	0	0	0	0	0
0	0	0	0	0	0	0	0	0	0	0	0	0	0	0	0	0	0	0	0	0	0	0	0	0
0	0	0	0	0	0	0	0	8	0	0	0	0	0	0	0	0	0	0	0	0	0	0	0	0
0	0	0	0	0	0	0	0	0	0	0	0	0	0	0	0	0	0	0	0	0	0	0	0	0
0	0	0	0	0	0	0	0	0	0	0	0	0	0	0	0	0	0	0	0	0	0	0	0	0
0	0	0	0	0	0	0	0	13	0	0	0	0	0	0	0	0	0	0	0	0	0	0	0	0
0	0	0	0	0	18	0	0	5	0	0	0	0	0	0	0	0	0	0	0	0	0	0	0	0
0	0	0	0	0	0	0	0	0	0	0	0	0	0	0	0	0	0	0	0	0	0	0	0	0
0	0	0	0	0	0	1	5	1	0	0	0	0	0	0	0	1	7	0	0	0	0	0	0	0
0	0	0	0	0	0	0	0	0	0	0	0	0	0	0	0	0	0	0	0	0	0	0	0	0
0	0	0	0	0	0	0	0	0	0	0	0	0	0	0	0	0	0	0	0	0	0	0	0	0
0	0	0	0	0	0	0	0	2	0	0	0	0	0	0	0	0	0	0	0	0	0	0	0	0
0	0	0	0	0	0	2	0	0	0	0	0	0	0	0	0	0	0	0	0	0	0	0	0	0
0	0	0	0	0	0	0	0	0	0	0	0	0	0	0	0	0	0	0	0	0	0	0	0	0
1	7	35	0	6	7	14	278	7	0	2	5	3	58	6	0	0	4	23	0	5	10	0	0	35
0	0	0	0	0	0	0	0	0	0	0	0	0	0	0	0	0	0	0	0	0	0	0	0	0
0	0	2	0	6	1	6	69	3	16	0	0	0	1	3	0	0	0	7	0	0	0	0	0	1
2	0	55	0	4	13	10	0	1	0	0	5	0	57	0	2	1	98	23	0	0	0	1	0	3
0	5	245	100	0	10	34	103	180	37	0	0	1	18	0	0	0	0	0	0	0	0	0	0	0
0	2	1	0	1	0	0	0	1	0	3	0	0	0	0	0	0	0	3	0	0	0	0	0	0
0	0	0	30	0	27	0	0	28	0	0	0	2	5	0	0	0	1	0	0	0	0	0	0	0
1	0	2	0	0	1	0	0	0	0	0	0	0	10	0	0	0	10	3	0	0	0	0	0	2
0	0	0	21	0	0	0	0	0	0	0	0	0	0	0	0	0	0	0	0	0	0	0	0	0
0	0	2	0	0	1	0	0	0	0	0	0	0	10	0	0	0	0	0	0	0	0	0	0	0
0	0	0	120	0	0	35	0	0	0	0	0	34	0	0	0	0	0	0	8	0	0	2	0	0
0	0	0	0	0	0	0	0	0	0	0	0	0	2	1	0	0	0	0	0	0	0	0	0	0
0	0	0	0	0	0	0	0	0	0	0	0	0	0	0	0	0	0	0	0	0	0	0	0	0
0	0	0	0	0	0	0	0	0	0	0	0	0	0	0	0	0	0	0	0	0	0	0	0	0
0	1	0	0	0	0	0	0	0	0	0	0	0	0	0	0	1	0	0	0	0	0	0	0	0
2	0	0	0	0	0	0	0	0	0	0	0	0	0	0	0	0	0	0	0	0	0	0	0	0
0	0	0	0	0	0	0	0	0	0	0	0	0	0	0	0	0	0	0	0	0	0	0	0	0
0	0	0	0	0	0	0	0	0	0	0	0	0	0	0	0	0	0	0	0	0	0	0	0	0
0	0	0	0	0	0	0	0	0	0	0	0	0	0	0	0	0	0	0	0	0	0	0	0	0
0	0	0	0	0	0	0	0	0	0	0	0	0	0	0	0	0	0	0	0	0	0	0	0	0
0	0	0	0	0	0	0	0	3	0	0	0	0	0	0	0	0	0	0	0	0	0	0	0	0
0	0	0	0	0	0	0	0	2	0	0	0	0	0	0	0	0	0	0	0	0	0	0	0	0

0	0	0	0	0	0	5	25	22	0	0	0	0	0	0	0	0	0	0	0	0	0	0	0	0
2	29	0	51	14	18	76	108	131	10	6	8	0	0	1	0	0	0	3	0	1	1	3	0	0
0	8	10	0	0	0	0	0	4	0	0	0	0	0	1	0	0	0	1	0	0	0	0	0	0
0	0	0	0	0	0	0	0	0	25	0	0	0	0	0	0	0	0	0	0	0	0	0	0	0
0	0	0	0	0	1	8	3	4	0	0	0	0	1	0	0	0	0	0	0	0	0	1	0	0
2	32	150	0	1	0	0	28	44	0	1	0	1	0	1	0	1	0	3	0	0	0	0	1	1
0	0	0	0	0	0	0	0	0	0	0	0	0	0	0	0	0	0	0	0	0	0	0	0	0
0	0	0	0	0	0	0	0	0	0	0	0	0	0	0	0	0	0	0	0	0	0	0	0	0
0	0	0	0	0	0	0	0	0	0	0	0	0	0	0	0	0	0	0	0	0	0	0	0	0
0	0	0	0	0	0	0	0	0	0	0	0	0	0	0	0	0	0	0	0	0	0	0	0	0
0	0	0	0	0	0	0	0	0	0	0	0	0	0	0	0	0	0	0	0	0	0	0	0	0
0	0	0	0	0	0	0	0	0	0	0	0	0	0	0	0	0	0	0	0	0	0	0	0	0
0	0	0	0	0	0	1	0	0	0	0	0	0	0	0	0	0	0	0	0	0	0	0	0	0
0	0	0	0	0	0	0	0	0	0	0	0	0	0	0	0	0	0	0	0	0	0	0	0	0
0	3	0	0	0	0	0	2	2	0	0	0	3	0	0	0	0	0	0	0	0	0	0	0	0
0	0	0	0	0	0	0	0	0	0	0	0	0	0	0	0	0	0	0	0	0	0	0	0	0
0	0	0	0	0	0	0	0	0	0	0	0	0	0	0	0	0	0	0	0	0	0	0	0	0
0	1	0	0	0	0	0	0	0	0	0	0	0	0	0	0	0	0	0	0	0	0	0	0	0
0	0	0	126	0	13	3	524	0	0	0	8	0	1	2	0	1	0	0	1	3	0	0	11	38
0	0	0	15	0	0	0	0	5	0	0	0	4	0	3	0	0	0	0	0	0	0	0	0	0
0	0	0	0	0	0	0	5	0	0	0	0	0	0	0	0	0	0	0	0	0	0	0	0	0
0	0	0	0	0	0	0	0	1	0	0	3	0	0	0	0	0	0	0	0	0	0	0	0	0
0	0	0	0	0	0	0	0	0	0	0	0	0	0	0	0	0	0	0	0	0	0	0	0	0
0	0	6	0	0	0	0	0	0	0	0	0	0	0	0	0	0	0	0	0	0	0	0	0	0
0	0	0	0	0	0	0	0	0	0	0	0	0	0	0	0	0	0	0	0	0	0	0	0	0
0	0	0	0	0	0	0	0	0	0	0	0	0	0	0	0	0	0	0	0	0	0	0	0	0
0	0	0	0	0	0	0	10	6	5	0	7	8	57	0	0	0	0	0	0	0	0	0	0	0
0	0	0	0	0	0	0	0	3	0	0	0	0	28	0	16	1	0	0	0	0	0	6	44	0
2	1	0	0	0	0	0	2	24	0	0	0	0	0	0	0	0	0	0	0	0	0	0	0	0
0	0	0	0	0	0	0	0	0	49	0	0	0	0	0	0	0	0	0	0	0	0	0	0	0
0	0	0	0	0	0	0	0	0	0	0	0	0	0	0	0	0	0	0	0	0	0	0	0	0
1	0	0	0	0	0	0	0	0	0	0	0	0	0	0	0	0	0	0	0	0	0	0	0	0
0	0	0	0	0	0	0	0	0	0	0	8	0	0	0	0	0	0	0	0	0	0	0	0	0
0	0	0	0	0	0	0	0	0	0	0	0	0	0	0	0	0	0	0	0	0	0	0	0	0
0	0	0	0	0	0	0	4	2	0	0	0	0	0	0	0	0	0	0	0	0	0	0	0	0
0	0	0	0	0	0	0	0	0	0	0	0	0	0	0	0	0	0	0	0	0	0	0	0	0
0	0	0	0	0	0	0	2	0	0	0	0	0	0	0	0	0	0	0	0	0	0	0	0	0
0	0	0	0	0	0	0	0	0	0	0	0	0	0	0	0	0	0	0	0	0	0	0	0	0
0	0	0	0	0	0	0	0	0	0	0	0	0	0	0	0	0	0	0	0	0	0	0	0	0
0	0	0	0	0	0	0	0	0	0	0	0	0	0	0	0	0	0	0	0	0	0	0	0	0
0	0	0	0	0	0	0	0	0	0	0	0	0	0	0	0	0	0	0	0	0	0	0	0	0
0	0	0	0	0	11	9	0	0	0	0	0	0	0	0	0	0	0	0	0	0	0	0	0	0
0	0	0	0	0	0	0	0	0	0	0	0	0	0	0	0	0	0	0	0	0	0	0	0	0
0	0	0	0	0	0	0	1	0	0	0	0	0	0	0	0	0	0	0	0	0	0	0	0	0
0	0	0	0	0	0	0	14	1	0	0	0	0	0	0	0	0	0	0	0	0	0	0	0	0
0	0	0	0	0	0	0	0	0	0	0	0	0	0	0	0	0	0	0	0	0	0	0	0	0
0	0	0	1	0	0	0	0	13	0	0	0	0	0	0	0	0	0	0	0	0	0	0	0	0
0	0	0	0	0	0	0	0	0	0	0	0	0	0	0	0	0	0	0	0	0	0	0	0	0
0	0	0	0	0	0	0	0	0	0	0	0	0	0	0	0	0	0	0	0	0	0	0	0	0
3095	398	4568	443	9374	4300	517	3518	11939	6996	2	1054	366	2026	51	15	40	342	407	484	613	70	40	131	98
3	19	54	0	69	264	24	99	202	162	1	22	12	0	6	2	1	9	0	0	10	9	1	0	0
3	1	0	81	10	27	1	278	19	25	0	0	97	16	2	1	7	17	0	0	3	0	0	0	0
6	2	34	1	4	33	2	19	93	22	0	5	9	12	0	0	11	30	2	15	13	0	0	4	4
0	14	0	5	0	20	0	0	9	48	0	0	0	0	22	13	20	8	81	223	54	0	0	0	0
39	3	11	2	71	0	6	6	68	6	0	3	0	64	0	0	13	0	13	4	8	0	0	0	0
0	2	0	0	2	37	0	15	82	12	0	2	6	0	0	0	0	0	0	0	0	0	1	0	0
0	0	2	0	7	0	1	1	1	0	0	1	0	1	0	0	0	0	0	0	1	0	0	0	0
0	0	0	0	0	0	0	1	0	0	0	0	0	1	0	0	0	0	0	0	1	0	0	0	0
0	0	1	0	0	0	0	0	2	0	0	0	0	0	0	0	0	0	0	0	0	0	0	0	0
0	0	0	0	0	0	0	0	0	0	0	0	0	0	0	0	0	0	0	0	0	0	0	0	0
1	0	0	0	0	0	0	7	0	0	0	0	0	0	0	1	0	0	0	0	0	0	0	0	0
0	0	0	0	0	0	0	0	5	0	0	1	10	0	0	0	2	0	0	0	0	0	0	0	0
0	0	0	0	0	0	0	6	0	0	0	0	0	0	0	0	0	0	0	0	0	0	0	0	0
0	0	0	0	0	0	0	0	0	0	0	0	0	0	0	0	0	0	0	0	0	0	0	0	0
0	0	1	0	0	0	0	0	0	0	0	0	0	0	0	0	0	0	0	0	0	0	0	0	0
0	0	0	0	6	27	0	0	0	0	0	0	0	0	0	0	0	0	0	0	0	0	0	0	0
0	0	0	0	0	0	0	0	8	0	0	0	0	0	0	0	0	0	0	0	0	0	0	0	0
0	0	0	0	0	0	0	0	0	0	0	0	0	0	0	8	0	0	0	0	0	0	2	0	0
0	0	0	0	0	0	0	0	0	0	0	0	0	0	0	0	0	0	0	0	0	0	0	0	0
0	0	0	25	0	2	0	0	0	0	0	0	0	0	0	0	0	0	0	0	0	0	0	0	0
0	0	0	0	0	1	0	0	0	0	0	0	9	0	0	0	0	0	0	0	0	0	0	0	0
0	0	21	3	0	0	1	16	0	0	0	0	0	0	0	0	0	0	0	0	0	0	0	0	0
0	0	0	0	0	0	0	1	0	0	0	0	0	0	0	0	0	0	0	0	0	0	0	0	0
0	0	0	0	0	0	0	0	0	0	0	0	0	0	0	0	0	0	0	0	0	0	0	0	0
0	0	0	0	0	0	0	14	0	0	0	0	0	0	0	0	0	0	0	0	0	0	0	0	0
3	16	0	79	6	46	34	25	53	20	1	6	5	1	0	0	1	3	0	7	15	0	1	10	12
0	2	96	15	0	77	23	61	98	25	0	0	16	12	2	0	0	0	354	14	0	0	2	0	0
0	0	0	1	0	0	5	1	19	2	0	0	0	0	0	0	0	0	0	0	3	1	0	2	14
0	1	0	0	0	0	0	2	0	1	0	0	10	0	0	0	6	0	0	0	0	0	0	0	0
0	3	0	0	0	0	1	6	0	22	0	0	3	3	0	0	0	0	0	0	1	0	0	0	0
0	0	0	0	0	0	0	2	0	0	0	0	0	2	1	0	0	2	2	0	2	0	0	6	0
0	0	0	0	0	0	0	0	0	0	0	0	1	0	0	0	0	0	0	0	0	0	0	0	0
0	0	0	0	0	0	0	6	0	0	0	0	0	0	0	0	0	0	0	0	0	0	0	0	0
0	0	0	0	1	0	0	0	0	0	0	0	0	0	0	0	0	0	0	0	0	0	0	0	0
0	4	0	0	0	1	17	102	53	233	0	33	65	19	12	8	1	30	144	26	19	11	1	40	35
0	0	0	0	0	0	0	0	0	0	0	0	1	0	1	0	0	0	0	0	0	0	0	0	0
2	0	0	60	0	6	17	10	7	0	0	2	0	0	0	0	0	0	0	0	0	0	1	0	0
2	0	0	1	0	19	1	0	2	2	0	0	1	0	0	0	0	0	0	0	0	0	1	0	0
0	0	0	0	0	33	0	10	0	0	0	0	0	0	0	0	0	0	0	0	0	2	0	0	0
0	0	0	0	0	0	0	0	0	0	0	0	0	0	0	0	0	0	0	0	0	0	0	0	0
0	0	0	0	0	0	0	0	0	0	0	0	0	0	0	0	0	0	0	0	0	0	0	0	0
0	0	0	0	0	0	0	0	0	0	0	0	0	0	2	0	0	0	0	0	0	0	0	0	0
0	0	0	0	0	0	14	17	12	0	0	0	1	0	0	0	3	1	0	0	2	0	0	0	0
0	0	28	31	0	0	0	0	8	19	0	0	0	0	0	0	1	0	0	0	1	2	0	0	0
0	0	0	0	0	0	0	0	1	0	0	0	0	0	0	0	0	0	0	0	0	0	0	0	0
0	0	0	0	0	0	0	0	0	0	0	0	0	0	0	0	0	0	0	0	0	0	0	0	0
0	0	0	0	0	0	0	0	0	0	0	0	0	0	0	0	0	0	0	0	0	0	0	0	0
5	0	0	0	0	0	0	0	0	0	0	0	0	0	0	0	0	0	0	0	0	0	0	0	0
0	0	0	0	0	0	0	0	0	0	0	0	0	0	0	3	0	0	0	2	0	0	0	0	0
0	0	0	0	0	0	0	0	21	0	0	0	0	0	0	0	0	0	0	0	0	0	0	0	0
0	3	52	122	0	0	0	73	0	0	0	1	8	0	0	0	0	0	0	0	0	1	0	0	0
0	0	0	0	0	5	0	0	0	0	0	18	9	2	0	0	0	0	0	0	0	2	1	0	8
1	0	31	1	2	72	18	9	21	3	0	9	3	4	3	6	4	3	40	0	1	10	13	0	11
0	0	0	0	0	21	0	0	0	0	0	3	1	0	0	0	0	0	0	0	0	0	0	0	6
0	0	0	0	0	0	0	0	0	0	0	0	2	5	0	0	0	0	0	0	0	0	0	0	0
0	0	0	0	0	0	1	0	0	0	0	0	0	0	0	0	0	0	0	0	0	0	0	0	0
0	2	0	0	0	0	13	0	46	158	0	2	0	0	18	2	8	0	5	5	14	0	0	0	0
0	0	0	0	0	0	1	0	0	4	0	0	0	0	6	0	68	0	0	0	6	0	0	0	0
0	0	0	0	0	0	0	0	2	0	0	0	0	0	0	0	0	0	0	0	0	0	0	0	0
0	0	0	0	7	0	0	3	1	0	0	0	0	1	0	26	1	3	0	34	0	0	0	0	2
0	0	0	0	0	0	0	0	0	0	0	0	0	0	0	0	0	0	0	0	0	0	0	0	0
0	0	0	0	0	0	0	0	0	0	0	1	0	1	4	0	0	0	0	0	7	0	0	0	0
0	0	0	0	0	0	0	0	0	2	0	0	3	0	0	0	0	0	0	0	0	0	0	0	0
0	0	90	0	0	0	0	0	0	0	0	0	0	0	0	0	0	0	0	0	0	0	0	0	0
0	0	0	0	0	0	0	0	7	0	0	0	3	0	0	0	1	0	0	0	0	0	0	0	0
0	0	0	0	0	0	0	0	1	0	0	0	1	0	0	0	0	0	0	0	0	0	0	0	0
0	0	0	0	0	0	0	0	0	0	0	0	0	0	0	0	0	0	0	0	0	0	0	0	0
0	7	0	0	0	0	0	0	0	0	0	0	0	0	0	0	0	0	0	0	0	0	0	0	0
0	0	0	0	0	0	0	0	0	0	0	0	0	0	0	0	0	0	0	0	0	0	1	0	0
0	0	18	27	0	0	0	0	0	0	0	0	0	8	0	0	0	0	0	0	0	0	0	0	0
0	0	0	0	0	0	0	0	0	0	0	0	0	0	0	0	0	0	0	0	0	0	0	0	0
0	0	0	0	0	0	0	0	0	0	0	0	0	0	0	0	0	0	0	0	0	0	0	0	0
0	0	0	0	0	0	10	0	0	0	0	0	0	0	4	0	0	0	0	0	0	0	0	0	0
0	0	0	0	0	0	0	0	0	0	0	0	0	0	0	0	0	0	0	0	0	0	0	0	0
0	0	0	0	0	0	0	0	0	0	0	0	0	0	0	0	0	0	0	0	0	0	0	0	0
0	0	0	0	0	0	0	0	0	0	0	0	0	0	0	0	0	0	0	4	0	0	0	0	0
0	0	0	23	0	3	0	0	0	0	0	0	0	0	0	0	0	0	0	0	0	0	0	0	0
0	0	0	0	0	24	0	0	0	0	0	0	0	0	0	0	0	0	0	0	0	0	0	0	0
0	0	0	0	0	0	0	0	15	0	0	0	0	0	0	0	0	0	0	0	0	0	0	0	0
0	0	0	0	0	0	0	0	0	0	0	0	0	0	0	5	0	0	0	0	0	0	0	0	0
2	0	5	6	0	12	1	26	31	0	0	1	13	0	0	0	0	0	0	0	0	0	0	0	6
0	5	33	1	0	32	15	3	66	77	0	6	1	2	0	0	0	5	0	1	0	0	0	0	0
0	0	0	0	0	0	0	0	16	13	0	0	0	0	0	0	0	0	0	0	0	0	0	0	0
0	0	0	0	0	0	0	0	0	0	0	0	0	0	0	0	0	0	0	0	0	0	0	0	8
0	0	0	0	0	1	0	1	1	0	0	0	0	0	0	0	0	0	0	0	0	0	0	0	0
2	5	94	51	7	124	7	50	27	34	0	26	2	4	0	0	1	0	13	0	7	0	1	0	1
0	1	1	57	0	4	0	9	4	0	0	6	0	7	1	0	0	0	1	0	0	5	0	0	0
0	0	15	8	0	23	1	96	120	115	1	70	1	2	1	0	0	1	56	2	0	0	0	2	0
0	0	0	0	0	0	0	0	0	1	0	3	1	0	0	0	0	0	1	0	0	0	0	0	0
0	0	0	68	2	42	0	0	0	0	0	27	0	0	0	1	0	0	19	0	0	0	0	0	0
0	0	0	0	0	0	0	0	0	0	0	0	0	0	0	0	0	0	0	0	0	0	0	0	0
1	0	0	75	2	32	0	26	4	0	0	2	2	1	0	0	0	0	0	0	1	0	0	0	2
0	0	0	0	0	0	0	0	0	0	0	0	0	0	0	0	2	0	0	0	0	0	0	0	0
0	0	0	0	0	0	0	0	0	0	0	0	0	0	0	0	0	0	0	0	0	0	0	0	0
0	0	0	0	0	0	0	0	0	0	0	0	0	0	0	0	0	0	0	0	0	0	0	0	0
0	0	0	0	0	0	0	2	0	0	0	0	0	0	0	0	0	0	0	0	0	0	0	0	0
0	0	0	0	0	0	0	0	1	1	0	0	0	0	0	0	0	0	0	0	0	0	0	0	0
0	0	70	0	0	98	0	91	196	0	0	10	0	23	6	0	1	0	0	5	4	0	0	2	19
10	27	106	242	22	284	44	279	135	142	1	4286	16	16	556	8	21	4	112	106	35	26	2	52	65
0	0	9	1	7	1	0	0	8	1	0	10	0	0	2	0	0	1	0	0	0	0	0	0	0
0	0	0	0	0	0	1	0	12	0	0	4	0	0	0	0	0	0	0	0	0	0	0	0	0
0	6	16	0	11	12	23	4	81	88	0	377	1	70	5	7	1	2	97	6	0	2	0	1	6
0	0	0	0	0	0	17	0	0	62	0	6	0	18	2	0	1	0	44	0	0	0	0	2	0
0	0	0	0	2	0	0	0	3	0	0	0	0	0	4	0	0	3	0	0	0	0	0	0	3
0	0	0	0	0	0	0	0	5	1	0	4	0	1	0	0	0	0	1	0	0	0	0	0	0
0	0	0	0	0	0	2	0	0	0	0	0	0	0	0	0	0	0	0	0	0	0	0	0	0
0	0	0	0	5	0	0	0	0	0	0	19	0	51	0	0	0	2	0	0	0	0	0	40	0
0	0	0	0	0	0	0	0	0	0	0	0	0	0	0	0	0	0	0	0	0	0	0	0	0
0	0	0	0	0	0	0	0	0	0	0	0	0	0	0	0	0	0	0	0	0	0	0	0	0
0	0	0	0	0	0	0	0	0	0	0	0	0	0	0	0	0	0	0	0	0	0	0	0	0
0	0	0	0	0	0	0	0	14	0	0	0	0	0	0	0	0	0	0	0	0	0	0	0	0
0	0	0	0	0	0	0	0	11	0	0	0	0	0	0	0	0	0	0	0	0	0	0	0	0
0	0	0	0	0	0	0	0	0	0	0	0	0	0	0	0	0	0	0	0	0	0	0	0	0
0	0	0	0	0	0	0	0	0	0	0	0	0	0	0	0	0	0	0	0	0	0	0	0	0
0	0	0	1	0	0	0	0	0	0	0	1	0	0	0	0	0	0	0	0	0	0	0	0	0
0	0	0	0	0	0	0	0	0	0	0	0	0	0	0	0	1	0	0	0	0	0	0	0	0
0	8	0	0	30	0	33	66	0	0	1	10	0	233	8	0	0	62	0	0	7	1	0	0	4
0	0	0	0	0	0	0	0	0	0	0	0	0	0	0	0	0	0	0	0	0	0	0	0	0
0	0	0	0	0	0	10	0	7	0	0	0	0	0	0	0	0	0	0	0	0	0	0	0	0
0	0	0	0	0	0	0	0	0	0	0	2	0	0	0	0	0	0	0	0	0	0	0	0	0
0	0	0	0	0	0	0	0	0	0	0	0	0	0	0	0	0	18	0	0	0	0	0	0	0
0	0	0	0	0	0	0	0	0	0	0	0	0	0	0	0	0	0	0	0	0	0	0	0	0
0	0	0	0	0	0	0	0	0	0	0	0	0	0	0	0	0	0	0	0	0	0	0	0	0
0	0	0	0	0	0	0	0	0	0	0	0	0	0	0	0	0	0	0	0	0	0	0	0	0
0	0	0	0	0	0	0	0	0	0	0	0	0	0	0	0	0	0	0	0	0	0	0	0	0
0	0	0	0	0	0	0	0	0	0	0	0	0	0	0	0	0	0	0	0	0	0	0	0	0
0	0	0	0	0	20	0	0	1	0	0	0	0	0	0	0	0	0	0	0	0	0	0	0	0
5	6	61	60	1	81	58	11	257	0	0	0	1	0	0	0	0	0	0	0	0	0	0	0	0
0	0	0	0	0	0	0	0	0	0	0	0	0	0	0	0	0	0	0	0	0	0	0	0	0
0	0	0	0	0	0	0	0	0	0	0	0	0	0	0	0	0	0	0	0	0	0	0	0	0
0	4	0	0	0	0	7	56	30	0	0	0	0	0	0	0	0	0	0	0	0	0	0	0	8
0	0	0	0	0	0	0	13	0	0	0	0	0	0	0	0	0	0	0	0	0	0	0	0	0
19932	15896	61862	40197	32880	46497	46711	48263	25903	48313	1	4	3	10	1	0	1	0	4	1	3	3	0	6	1928
0	1	19	8	0	0	0	0	14	0	0	0	0	0	0	0	0	0	1	0	0	0	0	0	0
3	1	1	0	3	0	0	0	2	0	0	0	0	0	0	0	0	0	0	0	0	0	0	0	0
10	1	1	1	137	1	0	1	16	5	0	0	0	0	0	0	0	0	0	0	0	0	0	0	15
0	4	219	38	2	84	41	261	49	81	0	0	0	0	0	0	0	0	0	0	0	0	0	0	0
16	0	0	0	12	0	0	0	0	0	0	0	0	0	0	0	0	0	0	0	0	0	0	0	0
0	0	15	0	0	1	1	0	0	0	0	0	0	0	0	0	0	0	0	0	0	0	0	0	0
0	0	0	0	0	0	0	0	0	0	0	0	0	0	0	0	0	0	0	0	0	0	0	0	0
0	0	0	0	0	0	0	0	0	0	0	0	0	0	0	0	0	0	0	0	0	0	0	0	0
47325	412	4227	642	55734	273	158	518	2126	867	0	10	5	2	2	0	1	2	7	3	2	5	0	2	1
243	162	119	32	41	585	25	264	840	66	0	27	1	3	0	0	0	0	2	1	0	2	0	3	0
0	0	0	0	1	6	0	10	0	8	0	2	533	0	1	0	0	0	21	32	0	3	7	1	0
12	4	15	3	39	0	0	2	18	4	0	16	0	0	0	0	0	0	0	0	0	0	0	0	0
0	2	0	0	1	0	0	16	3	4	0	0	0	0	0	0	0	0	0	0	0	0	0	0	0
0	0	0	0	0	0	0	20	0	0	0	0	0	0	0	0	0	0	1	0	0	0	0	0	0
4	3	3	131	0	30	17	150	11	0	0	2	6	0	3	0	1	0	0	0	7	0	0	0	0
0	0	2	35	2	0	0	15	1	4	0	0	0	0	0	0	0	0	0	0	0	0	0	0	0
0	0	0	0	4	52	0	82	8	1	0	363	0	4	12	0	96	2	80	8	10	2	0	0	14
0	4	0	14	0	58	0	0	3	0	0	0	0	20	0	0	0	0	0	11	0	0	0	0	0
0	0	0	0	0	0	0	0	0	0	0	0	0	0	0	0	0	0	0	0	0	0	0	0	0
0	2	0	0	2	0	0	0	0	2	0	0	0	0	0	0	0	0	0	0	0	0	0	0	0
0	0	0	0	0	1	0	0	0	0	0	0	0	0	0	0	0	0	0	0	0	0	0	0	0
1	10	109	0	2	0	13	16	11	0	1	0	3	24	1	0	1	3	0	6	27	0	1	57	19
0	0	0	0	0	0	0	0	0	0	0	0	0	0	0	0	0	0	0	0	0	0	0	0	0
0	0	0	0	0	0	0	0	0	0	0	0	0	0	0	0	0	0	0	0	0	0	0	0	0
0	0	10	0	0	5	0	0	4	0	0	0	0	0	0	0	0	0	0	0	0	0	0	0	0
0	0	0	0	0	0	0	0	0	0	0	0	0	0	0	0	0	0	0	0	0	0	0	0	0
0	0	0	0	0	0	0	0	11	0	0	0	0	0	0	0	0	0	0	0	0	0	0	0	0
0	0	0	0	0	0	0	0	0	0	0	0	0	0	0	0	0	0	0	0	0	0	0	0	0
0	0	0	0	0	0	0	0	0	0	0	0	0	0	0	0	0	1	0	0	0	0	0	0	0
0	0	0	0	0	0	0	0	0	0	0	0	0	0	0	0	0	0	0	0	0	0	0	0	0
0	0	0	1	0	0	0	0	0	0	0	0	0	0	0	0	0	0	0	0	0	0	0	0	0
0	0	0	0	0	0	0	0	0	0	0	0	0	0	0	0	0	0	0	0	3	0	0	0	0
0	1	0	0	0	0	0	0	1	0	0	0	0	0	0	0	0	0	0	0	0	0	0	0	0
36	5	399	58	137	571	122	433	778	102	0	0	0	0	0	0	0	0	0	0	0	0	0	0	0
0	0	0	0	0	0	0	6	1	0	0	0	0	0	0	0	0	0	0	0	0	0	0	0	0
1	3	24	0	7	10	2	153	95	0	0	5	0	0	0	0	0	0	0	0	0	0	0	0	0
0	0	0	0	0	0	0	0	0	0	0	0	0	0	0	0	0	0	0	0	0	0	0	0	0
2	3	1	0	24	0	0	0	0	0	0	0	0	0	0	0	0	0	0	0	0	0	0	0	0
0	0	0	0	0	0	0	0	0	0	0	0	0	0	0	0	0	0	0	0	0	0	0	0	0
1	0	2	0	25	0	0	9	9	0	0	7	3	23	11	0	21	1	14	1	0	3	0	15	34
0	0	0	0	4	0	0	0	0	0	0	0	0	0	0	0	0	0	0	0	0	0	0	0	0
0	0	0	0	0	0	0	0	0	0	0	0	0	0	0	0	0	0	0	0	0	0	0	0	0
51	0	0	0	77	0	1	4	2	0	1	0	1	0	778	0	0	0	0	0	0	0	0	1	0
4	0	1	0	309	0	0	1	128	0	0	0	0	5	0	0	3	1	8	1	0	0	0	4	5
1	3	0	0	4489	0	0	10	2	0	0	0	0	0	1	0	70	0	0	0	0	0	0	0	0
0	0	0	0	0	0	0	0	0	0	0	0	0	0	0	0	0	0	0	0	0	0	0	0	0
0	0	0	0	0	0	0	0	0	3	0	41	0	0	0	0	1	0	2	0	0	0	0	42	0
0	0	0	0	2	0	0	0	1	0	0	0	0	2	0	0	0	0	0	0	0	0	0	1	1
0	0	0	0	17	7	0	0	4	4	0	0	1	0	0	0	3109	3	8063	0	0	2	0	0	0
0	0	0	6	0	0	0	0	3	0	0	0	1	0	0	0	0	0	0	0	0	0	0	0	0
0	0	0	0	0	0	0	6	0	0	0	0	0	0	0	0	0	0	0	0	0	0	0	0	0
0	0	0	0	0	0	0	0	0	0	0	0	0	0	0	0	24	0	10	0	0	0	0	0	0
0	1	0	0	5	0	6	10	15	0	0	21	7	45	15	0	2	2	17	0	0	0	0	10	0
0	0	0	0	9	0	0	0	0	0	0	0	0	0	251	0	0	0	0	1	0	0	0	0	0
0	0	0	0	0	0	0	0	0	0	0	0	0	3	0	0	0	0	0	0	0	0	0	0	0
0	0	0	0	1	0	0	0	0	0	0	0	0	0	0	0	0	0	0	0	0	0	0	0	0
0	0	0	0	0	0	10	0	0	0	0	0	0	0	0	0	0	0	0	0	0	0	0	0	0
0	0	0	0	0	0	0	0	0	0	0	0	0	0	0	0	0	0	0	0	0	0	0	0	0
0	0	0	0	0	0	0	0	0	0	0	0	0	0	0	0	0	0	0	0	0	0	0	1	0
0	0	0	0	0	1	0	0	0	0	0	0	0	0	2	0	0	0	0	0	0	0	0	0	0
0	0	0	0	0	0	0	0	0	0	0	1	0	0	4	0	1	0	2	0	0	0	0	0	0
0	0	0	0	1	0	0	0	0	4	0	0	0	0	0	0	0	0	0	0	0	0	0	0	0
0	0	0	0	0	0	0	0	1	0	0	0	0	0	0	0	0	0	0	0	0	0	0	0	0
0	0	0	0	0	0	0	0	0	0	0	0	0	0	0	0	0	0	0	0	0	0	0	0	0
0	0	0	0	0	0	0	0	0	0	0	0	0	0	0	0	0	0	0	0	0	0	0	0	0
0	0	0	0	0	0	0	0	0	0	0	0	0	0	0	0	0	0	0	0	0	0	0	0	0
0	0	0	0	0	0	0	0	0	0	0	0	0	0	0	0	0	0	0	0	0	0	0	0	0
0	0	0	0	2	0	0	0	1	0	0	0	0	0	0	0	0	0	0	0	0	0	0	0	0
0	0	0	0	0	0	0	0	0	0	0	0	0	0	0	0	0	0	0	0	0	0	0	0	0
0	0	0	0	0	0	0	0	0	0	0	0	0	0	0	0	0	0	0	0	0	0	0	0	0
0	0	0	0	0	0	0	0	0	0	0	0	0	0	0	0	0	0	0	0	0	0	0	0	0
0	0	0	0	0	0	0	0	0	0	0	0	0	0	0	0	0	0	0	0	0	0	0	0	0

2	1	90	4	5	38	0	104	1095	15	0	0	0	0	0	0	0	0	0	0	0	0	0	0	0
0	0	0	0	0	0	0	0	0	0	0	0	0	0	0	0	0	0	0	0	0	0	0	0	0
0	0	0	0	0	19	0	8	32	9	0	0	0	0	0	0	0	0	0	0	0	0	0	0	0
0	0	0	0	0	0	0	0	0	0	0	0	0	0	0	0	0	0	0	0	0	0	0	0	0
151	16	0	0	2	8	0	0	6	0	0	0	0	0	0	0	0	0	0	0	0	0	0	0	0
1	0	0	0	0	0	0	0	0	0	0	0	0	0	0	0	0	0	0	0	0	0	0	0	0
0	0	0	0	0	0	0	0	1	0	0	0	0	0	0	0	0	0	0	0	0	0	0	0	0
0	0	0	0	0	0	0	0	0	0	0	0	0	0	0	0	0	0	0	0	0	0	0	0	0
0	0	0	0	0	0	0	0	0	0	0	0	0	0	0	0	0	0	0	0	0	0	0	0	0
0	0	0	0	0	0	0	0	0	0	0	0	0	0	0	0	0	0	0	0	0	0	0	0	0
0	0	0	0	0	0	0	0	3	0	0	0	0	0	0	0	0	0	0	0	0	0	0	0	0
0	3	6	0	1	1	6	5	20	3	0	0	0	0	0	0	0	0	0	0	0	3	0	0	0
0	0	0	0	0	0	0	0	0	0	0	0	0	0	0	0	0	0	0	0	0	0	0	0	0
0	0	0	0	0	0	0	0	0	0	0	0	0	0	0	0	0	0	0	0	0	0	0	0	0
0	0	0	0	0	0	0	0	0	0	0	0	0	0	0	0	0	0	0	0	0	0	0	0	0
0	0	0	0	0	0	0	0	0	0	0	0	0	0	0	0	0	0	0	0	0	0	0	0	0
0	0	0	0	0	0	0	0	0	0	0	0	0	0	0	0	0	0	0	0	0	0	0	0	0
1	4	33	1	6	11	0	14	201	0	0	0	0	0	0	0	0	0	0	0	0	0	0	0	0
0	2	0	0	0	0	0	0	0	0	0	0	0	0	0	0	0	0	0	0	0	0	0	0	0
0	0	0	0	0	0	0	0	0	0	0	0	0	0	0	0	0	0	0	0	0	0	0	0	0
0	0	0	0	0	0	0	5	0	0	0	0	0	0	0	0	0	0	0	0	0	0	0	0	0
0	1	641	6	4	8	0	0	5	0	0	0	0	0	0	0	0	0	2	0	0	0	0	0	0
0	0	13	0	0	0	0	0	0	0	0	0	0	0	0	0	0	0	0	0	0	0	0	0	0
0	0	0	0	0	0	0	0	0	0	0	0	0	0	0	0	0	0	0	0	0	0	0	0	0
0	0	0	0	0	0	0	0	1	0	0	0	0	0	0	0	0	0	0	0	0	0	0	0	0
1	0	0	0	0	0	0	0	0	0	0	0	0	0	0	0	0	0	0	0	0	0	0	0	0
0	42	418	10	11	31	6	883	27	39	0	0	0	0	0	0	0	0	1	0	0	0	0	0	0
0	0	0	0	0	11	7	0	0	0	0	0	0	0	0	0	0	0	0	0	0	0	0	0	0
0	0	0	0	0	0	0	0	0	0	0	0	0	0	0	0	0	0	0	0	0	1	0	0	0
0	0	0	0	0	0	2	0	0	0	0	0	0	0	0	0	0	0	0	0	0	0	0	0	0
0	0	0	0	0	3	0	4	0	1	0	0	0	0	0	0	0	0	0	0	0	0	0	0	1
0	0	0	0	0	0	0	0	0	0	0	0	0	0	0	0	0	0	0	0	0	0	0	0	0
0	0	0	0	0	0	0	0	0	0	0	0	0	0	0	0	0	0	0	0	0	0	0	0	0
0	0	0	0	0	0	0	0	0	0	0	0	0	0	0	0	0	0	0	0	0	0	0	0	0
0	0	0	0	0	0	0	0	0	0	0	0	0	0	0	0	0	0	0	0	0	0	0	0	0
0	0	0	0	0	0	0	0	0	0	0	0	0	0	0	0	0	0	0	0	0	0	0	0	0
0	0	0	0	0	0	0	0	0	0	0	0	0	0	0	0	0	0	0	0	0	0	0	0	0
0	0	0	0	0	0	0	0	0	0	0	0	0	0	0	0	0	0	0	0	0	0	0	0	0
0	0	0	0	0	0	0	0	0	0	0	0	0	0	0	0	0	0	0	0	0	0	0	0	0
0	0	0	0	0	0	0	0	0	0	0	0	0	0	0	0	0	0	0	0	0	0	0	0	0
0	0	0	0	0	0	0	0	0	0	0	0	0	0	0	0	0	0	0	0	0	0	0	0	0
0	0	0	0	0	0	0	0	0	0	0	0	0	0	0	0	0	0	0	0	0	0	0	0	0
0	0	0	0	0	0	0	0	0	0	0	0	0	0	0	0	0	0	0	0	0	0	0	0	0
0	0	0	0	0	0	0	0	5	0	0	0	0	0	0	0	0	0	0	0	0	0	0	0	0
0	0	0	0	0	0	0	0	0	0	0	0	0	0	0	0	0	0	0	0	0	0	0	0	0
3	22	1401	133	2	248	412	208	681	198	0	5	0	8	0	0	0	0	0	5	11	6	0	0	41
2	10	32	46	15	54	8	34	17	116	0	0	0	0	0	1	1	0	0	2	0	1	0	0	22
0	0	0	0	0	0	0	293	11	29	0	0	13	0	0	0	0	1	1	30	0	7	0	8	18
0	0	0	0	0	0	0	0	0	0	0	0	0	0	0	1	0	0	0	0	0	0	1	0	0
0	10	251	41	13	0	46	0	0	0	0	0	0	0	0	2	0	0	0	0	0	0	0	0	0
0	0	0	0	0	0	0	0	0	0	0	0	0	1	0	0	0	0	20	0	0	0	0	0	0
0	0	0	0	0	0	0	2	34	0	0	0	0	0	0	0	0	0	0	0	0	0	0	0	0
0	0	0	0	0	0	0	5	6	11	0	0	0	0	0	0	0	0	0	0	0	0	0	0	0
3	26	464	0	3	0	15	26	39	26	0	3	4	46	0	0	0	10	15	1	0	0	1	0	19
0	7	0	0	0	0	1	58	16	0	0	0	9	0	0	0	0	0	9	0	0	0	0	0	0
2	0	10	0	9	0	27	7	128	0	0	15	2	0	0	0	0	0	0	0	0	6	1	0	0
0	3	0	0	0	0	0	0	0	0	0	0	0	0	0	0	0	0	0	0	0	0	0	0	0
0	0	0	1	0	8	2	0	1	0	0	0	0	0	0	0	0	0	0	0	0	0	0	0	0
1	0	0	0	4	0	1	1	35	0	0	7	0	0	0	0	0	0	1	0	0	0	0	0	0
1	0	0	0	0	0	0	0	0	0	0	0	0	0	0	0	0	0	0	0	0	0	0	0	0
0	0	0	0	0	0	0	0	5	0	0	0	0	0	0	0	0	0	0	0	0	0	0	0	0
0	0	0	0	11	0	1	0	1	0	0	0	0	0	0	0	0	0	0	0	0	0	0	0	0
0	8	24	2	12	2	10	17	4	47	0	0	0	0	0	0	0	0	0	0	0	0	1	0	0
0	0	0	0	0	0	10	1	4	0	0	0	0	0	0	0	0	0	0	0	0	0	0	0	0
0	0	0	0	0	0	0	0	0	0	0	0	0	0	0	0	0	0	0	0	0	0	0	0	0
0	1	0	0	0	0	0	11	0	0	0	0	0	0	0	0	0	0	0	0	0	0	0	0	0
0	0	0	0	0	0	0	0	3	0	0	0	0	0	0	0	0	0	0	0	0	0	0	0	0
0	0	0	0	0	0	0	0	0	0	0	0	0	0	0	0	0	0	0	0	0	0	0	0	0
0	0	0	0	0	0	0	4	0	0	0	0	0	0	0	0	0	0	0	0	0	0	0	0	0
0	0	0	0	1	0	0	0	0	0	0	0	0	0	0	0	0	0	0	0	0	0	0	0	0
0	0	0	0	9	0	0	0	0	0	0	0	0	0	0	0	0	0	0	0	0	0	0	0	0
0	0	0	0	0	0	0	0	0	28	0	0	0	0	0	0	0	0	0	0	0	0	0	0	0
0	0	0	0	0	0	0	0	0	0	0	0	0	0	0	0	0	0	0	0	0	0	0	0	0
0	0	0	4	0	0	0	0	0	0	0	0	0	0	0	0	0	0	0	0	0	0	0	0	0
38	34	4111	449	135	583	1243	3582	1315	214	0	0	0	0	0	0	0	0	0	0	0	0	0	0	0
0	0	0	0	0	0	0	0	0	0	0	0	0	0	0	1	0	0	0	0	0	0	0	0	0
0	2	37	8	2	157	0	26	283	0	0	0	0	5	0	0	0	4	1	0	10	1	0	0	5
0	0	0	0	0	0	0	36	0	0	0	0	2	0	0	0	0	0	0	0	0	0	2	0	0
418	1192	24	8295	1994	0	1	0	0	6235	0	0	0	0	0	0	0	0	0	0	4	0	0	0	0
0	0	0	21	0	0	0	0	0	61	0	0	0	0	0	0	0	0	0	0	0	0	0	0	0
1	0	0	0	9	0	0	0	0	0	0	0	0	0	0	0	0	0	0	0	0	0	0	0	0
0	0	0	0	0	0	0	0	17	0	0	0	0	0	0	0	0	0	0	0	0	0	0	0	0
25	1	12	0	53	56	3	0	59	0	0	0	0	0	1	0	0	0	4	1	0	0	0	1	0
0	0	13	5	0	6	3	2	15	0	0	0	0	0	0	0	0	0	0	0	0	0	0	0	0
0	0	0	37	0	0	0	0	103	0	0	0	0	0	0	0	0	0	0	0	0	0	0	0	0
4	0	0	0	9	0	8	0	8	0	0	0	0	0	0	0	0	0	0	0	0	0	0	0	0
0	0	0	0	0	1	0	0	1	0	0	0	0	0	0	0	0	0	0	0	0	0	0	0	0
0	0	0	18	5	18	17	0	3	1	0	0	0	0	0	0	0	4	0	0	0	0	0	0	0
0	0	3	0	0	0	0	0	0	0	0	0	0	0	0	0	0	0	0	0	0	0	0	0	0
0	1	0	0	0	0	0	5	35	0	0	0	0	0	0	0	0	0	0	0	0	0	0	0	0
0	0	0	0	0	0	0	0	0	0	0	0	0	0	0	0	0	0	0	0	0	0	0	0	0
0	0	0	0	0	0	0	0	0	0	0	0	0	0	0	0	0	0	0	0	0	0	0	0	0
0	0	0	0	0	8	0	0	1	0	0	0	0	0	0	0	0	0	0	0	0	0	0	0	0
0	0	0	0	0	0	0	0	5	0	0	0	0	0	0	0	0	0	0	0	0	0	0	0	0
0	3	56	9	3	12	0	1	7	671	0	42	1	2	1	2	1	18	0	0	0	1	0	0	0
0	1	0	0	0	0	4	1	6	0	0	0	0	0	0	0	0	2	0	0	0	0	0	0	0
0	0	26	0	0	0	0	1	0	0	0	2	0	0	0	0	0	0	9	0	0	0	0	0	0
0	0	0	0	0	36	0	0	22	0	0	0	0	0	0	0	0	0	0	0	0	0	0	0	0
0	0	0	0	0	0	0	0	0	2	0	0	0	0	0	0	0	0	0	0	0	0	0	0	0
0	1	0	0	0	0	0	0	0	0	0	0	0	0	0	0	0	0	0	0	0	0	0	0	0
0	0	0	0	0	0	0	0	0	0	0	0	0	0	0	0	0	0	0	0	0	0	0	0	0
0	0	0	0	0	0	0	0	0	0	0	0	0	0	0	0	0	0	0	0	0	0	0	0	0
0	0	0	0	0	0	0	0	0	0	0	0	0	0	0	2	0	0	0	0	0	0	0	0	0
0	0	0	0	0	0	0	0	0	0	0	0	0	0	0	0	0	0	0	0	0	0	0	0	0
0	2	0	0	22	14	0	0	0	0	0	0	0	0	0	0	0	0	0	0	0	6	0	0	0
0	0	0	0	0	0	0	0	0	0	0	0	0	0	2	0	0	2	0	0	1	0	0	0	0
0	0	0	0	0	0	0	0	0	0	0	0	0	0	0	0	0	0	0	0	0	0	0	0	0
0	0	0	0	0	0	0	0	0	0	0	0	0	0	0	0	0	0	0	0	0	0	0	0	0
0	0	0	0	0	0	0	0	0	0	0	0	0	0	0	0	0	0	0	0	0	0	0	0	0
0	0	0	0	0	0	0	0	0	0	0	0	0	0	0	0	0	0	0	0	0	0	0	0	0
0	0	0	0	0	0	0	0	0	0	0	0	0	4	6	2	0	0	0	0	0	0	0	2	0
0	0	0	0	0	0	0	0	0	0	0	0	0	0	0	0	0	0	0	0	0	0	0	0	0
0	0	0	0	0	0	0	0	0	0	0	0	0	0	0	0	0	0	0	0	0	0	0	0	0
0	0	0	0	0	0	0	0	0	0	0	0	0	0	0	0	0	0	0	0	0	0	0	0	0
0	0	0	59	0	0	0	0	0	0	0	0	0	0	0	0	0	0	0	0	0	0	0	0	0
0	0	0	0	0	0	0	0	0	0	0	0	0	0	0	0	0	0	0	0	0	0	0	0	0
0	0	0	0	0	0	0	0	0	0	0	0	0	0	0	0	0	0	0	0	0	0	0	0	0
2	0	1	0	0	95	0	49	190	11	0	0	0	0	0	0	0	0	0	0	0	0	0	0	0
0	0	0	0	0	6	2	75	2	14	0	0	5	0	0	0	0	1	0	0	0	0	0	0	0
0	0	0	0	0	0	0	0	0	0	0	0	0	0	0	0	0	0	0	0	0	0	0	0	0
2	0	0	0	0	0	0	0	0	0	0	0	0	0	0	0	0	0	0	0	0	0	0	0	0
0	0	0	0	0	0	1	0	0	0	0	0	0	0	0	0	0	0	0	0	0	0	0	0	0
3	0	0	0	0	0	1	0	0	0	0	0	0	0	0	0	0	0	0	0	0	0	0	0	0
0	0	0	0	0	0	0	2	0	0	0	0	0	0	0	0	0	0	0	0	0	0	0	0	0
0	0	0	0	0	0	0	0	0	0	0	0	0	0	0	0	0	0	0	0	0	0	0	0	0
0	0	0	0	0	0	0	0	0	0	0	0	0	0	0	0	0	0	0	0	0	0	0	0	0
0	0	0	0	0	0	0	0	0	0	0	0	0	0	0	0	0	0	0	0	0	0	0	0	0
0	0	0	0	0	0	0	0	0	0	0	0	0	0	0	0	0	0	0	0	0	0	0	0	0
0	0	0	0	0	0	0	1	0	0	0	0	0	0	0	0	0	0	0	0	0	0	0	0	0
0	0	0	0	0	0	0	0	57	0	0	0	0	0	0	0	0	0	0	0	0	0	0	0	0
0	0	0	0	0	0	0	0	0	0	0	0	0	0	0	0	0	0	0	0	0	0	0	0	0
0	0	21	0	0	0	12	0	0	0	0	0	0	0	0	0	0	0	0	0	0	0	0	0	0
0	6	0	1	3	0	5	37	28	7	4	0	0	0	0	0	0	0	2	0	0	0	2	0	0
0	2	0	0	0	0	11	73	15	23	0	2	8	0	0	0	1	1	3	1	0	1	3	1	0
0	0	0	0	0	0	0	0	1	0	0	0	0	0	0	0	0	0	0	0	0	0	0	0	0
0	0	0	0	0	0	0	0	0	0	0	0	0	0	0	0	0	0	0	0	0	0	0	0	0
0	0	0	0	0	0	0	0	0	0	0	0	0	0	0	0	0	0	0	0	0	0	0	0	0
0	0	0	144	0	0	0	55	33	0	0	0	8	0	0	0	0	0	0	0	0	1	0	0	0
0	4	0	0	0	0	0	0	0	0	0	0	0	0	0	0	0	0	0	0	0	0	0	0	0
0	0	0	0	0	0	0	0	0	0	0	0	0	0	0	0	0	0	0	0	0	0	0	0	0
0	0	0	0	0	0	0	0	0	0	0	0	0	0	0	0	0	0	0	0	0	0	0	0	0
0	0	0	0	0	0	0	0	0	0	0	0	0	0	0	0	0	0	0	0	0	0	0	0	0
0	0	0	0	0	0	0	0	0	0	0	0	0	0	0	0	0	0	0	0	0	0	0	0	0
0	0	0	0	0	0	11	0	6	0	0	0	0	0	0	0	0	0	0	0	0	0	0	0	0
0	0	0	0	0	0	9	0	0	0	0	0	0	0	0	0	0	0	0	0	0	0	0	0	0
0	0	0	0	0	0	0	0	0	0	0	0	0	0	0	0	0	0	0	0	0	0	0	0	0
0	0	0	0	0	0	0	0	0	0	16	175	69	818	264	225	107	89	880	146	404	204	343	2495	174
0	0	0	2	0	0	0	0	0	0	0	0	0	0	0	0	0	0	0	0	0	0	0	0	0
0	0	0	0	0	0	0	0	0	0	0	0	0	0	0	0	0	0	0	0	0	0	0	0	0
0	0	0	0	0	0	0	0	0	0	0	0	0	0	0	0	0	0	0	0	0	0	0	0	0
0	0	0	0	0	0	0	0	0	0	0	0	0	0	0	0	0	0	0	0	0	0	0	0	0
0	0	0	0	0	0	12	0	0	0	0	0	0	0	0	0	0	0	0	0	0	0	0	0	0
0	0	0	0	0	0	0	0	0	0	0	0	0	0	0	0	0	0	0	0	0	0	0	0	0
0	0	0	0	0	0	0	0	0	0	0	0	0	0	0	0	0	0	0	0	0	0	0	0	0
0	0	0	0	0	24	2	0	4	0	0	0	0	0	0	0	0	0	0	0	0	0	0	0	0
0	0	0	0	0	0	0	2	0	0	0	0	0	0	0	0	0	0	0	0	0	0	0	0	0
0	0	0	0	2	0	0	0	0	0	0	0	0	0	0	0	0	0	0	0	0	0	0	0	0
0	0	0	0	0	0	1	0	0	0	0	0	0	0	0	0	0	0	0	0	0	0	0	0	0
0	0	0	0	1	0	0	0	0	0	0	0	0	0	0	0	0	0	0	0	0	0	0	0	0
0	0	0	0	1	0	0	0	0	0	0	0	0	0	0	0	0	0	0	0	0	0	0	0	0
0	0	0	0	0	0	0	31	6	0	0	0	0	0	0	0	0	0	0	0	0	0	0	0	0
0	0	0	0	0	0	0	0	0	0	0	0	0	0	0	0	0	1	0	0	0	0	0	0	0
0	0	0	0	0	0	0	0	0	0	0	0	0	0	0	0	0	0	0	0	0	0	0	0	0
0	0	0	0	0	0	1	0	4	0	0	0	0	0	0	0	0	0	0	0	0	0	0	0	0
0	0	0	0	0	0	0	0	1	0	0	0	0	0	0	0	0	0	0	0	0	0	0	0	0
0	0	0	0	0	0	0	0	0	0	0	0	0	0	0	0	0	0	0	0	0	0	0	0	0
0	0	0	0	0	0	0	0	0	0	0	0	0	0	0	0	0	0	0	0	0	0	0	0	0
0	0	0	0	0	0	0	44	0	0	0	0	0	0	0	0	0	0	0	0	0	0	0	0	0
0	0	0	0	0	0	0	0	0	0	0	0	0	0	0	0	0	0	0	0	0	0	0	0	0
0	0	0	0	0	0	0	0	0	0	0	0	0	0	0	0	0	0	0	0	0	0	0	0	0
0	0	0	0	0	0	0	20	11	0	0	0	0	0	0	0	0	0	0	0	0	0	1	0	0
0	0	0	0	0	0	0	0	0	0	0	0	0	0	0	0	0	0	0	0	0	0	0	0	0
0	0	0	0	0	0	0	0	0	20	0	0	0	0	0	0	0	0	0	0	0	39	0	0	0
0	0	0	0	0	0	0	0	0	0	0	0	0	0	0	0	0	0	0	0	0	0	0	0	0
0	0	0	0	0	0	5	6	0	0	0	0	0	0	0	0	0	0	0	0	0	0	0	0	0
0	0	0	0	0	18	0	0	0	0	0	0	1	0	0	0	0	0	0	0	0	0	0	0	0
0	0	0	0	0	0	0	0	0	0	0	0	0	0	0	0	0	0	0	0	0	0	0	0	0
0	0	0	0	0	0	0	0	0	0	0	0	0	0	0	0	0	0	0	0	0	0	0	0	0
0	0	0	0	0	0	0	0	0	0	0	0	0	0	0	0	0	0	0	0	0	0	0	0	0
0	0	0	0	0	0	0	0	0	0	0	0	0	0	0	0	0	0	0	0	0	0	0	0	0
0	0	0	0	0	0	0	0	0	0	0	0	0	0	0	0	0	0	0	0	0	0	0	0	0
0	0	0	0	0	0	0	0	0	0	0	0	0	0	0	0	0	0	0	0	0	0	0	0	0
0	0	0	0	0	0	0	0	0	0	0	0	0	0	0	0	0	0	0	0	0	0	0	0	0
0	0	0	0	0	0	0	0	0	0	0	0	0	0	0	0	0	0	0	0	0	0	0	0	0
0	1	0	0	0	0	14	5	6	0	0	5	0	0	0	0	0	0	0	0	0	0	0	3	1
0	4	0	0	0	1	2	0	0	0	0	0	0	0	0	0	0	0	0	0	0	0	0	0	0
0	0	0	0	0	0	0	0	0	0	0	0	0	0	0	0	0	0	0	0	0	0	0	0	0
0	0	0	0	0	0	0	0	0	0	0	0	0	0	0	0	0	0	0	0	0	0	0	0	0
0	0	0	0	0	0	0	0	0	0	0	0	0	0	0	0	0	0	0	0	0	0	0	0	0
97	11759	792	7103	22887	1883	599	910	623	5083	59684	31158	61389	32374	34846	63183	43415	69725	63144	65777	82848	78028	36506	68093	41077
0	0	0	1	0	84	0	17	10	74	0	0	4	0	0	0	0	0	0	0	0	0	0	0	0
3	2	0	0	0	0	0	28	26	0	0	0	0	0	0	0	0	1	19	0	0	0	0	38	0
0	2	13	0	0	0	0	0	0	0	0	0	3	3	0	0	0	0	0	0	0	0	0	0	0
0	0	0	0	0	0	0	0	0	0	0	0	0	0	0	0	0	0	1	0	0	0	0	0	0
145	50	169	144	63	2609	41	160	216	75	0	19	38	89	4	1	3	4	98	1	9	10	428	36	451
1	0	0	0	0	13	0	9	23	0	0	0	0	3	0	0	0	1	0	0	0	0	0	0	0
0	4	0	0	0	0	0	24	22	8	0	0	0	0	0	0	0	0	0	0	0	0	2	0	1
0	0	2	0	1	0	0	0	0	0	0	0	0	0	0	0	0	0	0	0	0	0	0	0	0
0	0	0	0	1	0	0	0	1	0	0	0	2	0	2	0	2	0	18	0	0	0	0	1	0
0	0	0	0	0	0	0	0	1	0	0	0	0	0	0	0	0	0	0	0	0	0	0	0	0
0	0	0	0	14	0	0	0	0	0	0	0	0	0	0	0	0	0	0	0	0	0	0	0	0
0	0	0	0	0	45	0	0	0	0	0	0	0	0	0	0	0	0	0	0	0	0	0	0	0
0	0	0	0	0	0	0	0	0	0	0	0	0	0	0	0	0	0	0	0	0	0	0	0	0
0	0	0	0	0	9	0	25	0	0	0	0	0	0	0	0	0	0	0	0	0	0	0	0	0
2	0	16	12	0	0	0	17	0	0	0	0	0	0	0	0	4	0	0	0	0	4	0	0	0
0	0	0	0	0	0	0	0	0	0	0	0	0	0	0	0	0	0	0	0	0	0	0	0	0
0	1	0	0	0	29	0	0	0	1	0	0	0	0	0	0	0	0	0	0	0	0	0	0	0
0	0	0	0	0	0	0	0	0	0	0	0	0	0	0	0	0	0	0	0	0	0	0	0	0
0	22	0	138	0	46	84	126	62	72	0	6	0	0	0	0	0	0	18	0	0	0	0	6	20
0	2	0	0	0	0	0	17	16	0	0	0	0	0	0	0	0	0	0	0	0	0	0	0	0
0	0	0	25	9	0	1	29	2	0	0	0	0	0	0	0	0	0	0	0	0	0	0	0	0
0	7	35	0	0	11	11	50	6	2	0	0	1	37	0	0	0	0	0	0	8	0	0	0	0
0	0	0	0	2	0	0	3	13	0	0	0	0	0	0	0	0	0	0	0	0	0	0	0	0
0	0	0	0	0	0	0	47	0	13	0	0	0	0	0	0	0	0	0	0	0	0	0	0	0
0	0	0	3	3	0	16	26	0	0	0	0	1	0	0	0	0	0	0	0	0	0	0	0	0
0	0	0	0	0	0	0	0	0	0	0	0	0	0	0	0	0	0	0	0	0	0	0	0	0
0	5	0	0	0	0	0	64	0	0	0	0	0	0	0	0	1	0	0	0	0	0	0	0	0
0	0	0	0	0	0	0	0	0	0	0	0	0	0	0	0	0	0	0	1	0	0	0	0	0
0	0	0	0	0	0	32	0	0	0	0	0	0	0	1	0	0	0	0	0	0	0	0	0	0
0	0	0	0	0	0	0	2	3	1	0	0	0	0	0	0	0	0	0	0	0	0	0	0	0
0	0	0	0	0	0	0	0	0	0	0	0	1	0	0	0	0	0	0	0	0	0	0	0	0
3	0	0	0	0	0	0	0	0	0	0	0	0	0	0	0	0	0	0	0	0	0	0	0	0
0	0	0	0	0	0	0	0	0	0	0	0	1	0	0	0	0	1	0	0	0	0	0	0	0
0	0	0	0	0	0	0	1	0	0	0	0	0	0	0	0	0	0	0	0	0	0	0	0	0
0	10	39	0	0	0	13	66	18	19	1	6	3	0	0	0	0	0	1	0	0	0	1	0	0
0	49	0	0	0	146	220	79	44	44	0	0	0	0	0	0	0	0	0	0	8	0	0	2	0
0	0	0	0	0	0	74	37	8	0	0	0	0	0	0	0	0	0	0	0	0	0	0	0	0
0	0	0	0	0	0	0	0	0	0	0	0	0	0	0	0	0	0	0	0	0	0	0	0	0
0	0	0	0	0	33	0	0	0	0	0	0	0	0	0	0	0	0	0	0	0	0	0	0	0
0	0	0	0	0	0	0	0	0	0	0	0	0	0	0	0	0	0	1	0	0	0	1	0	0
0	0	0	0	0	0	0	1	0	0	0	0	0	0	0	0	0	0	0	0	0	0	0	0	0
0	0	0	0	0	0	0	0	0	0	0	0	0	0	0	0	0	0	0	0	0	0	0	0	0
0	0	0	0	0	0	1	0	0	0	0	0	0	0	0	0	0	0	0	0	0	0	0	0	0
0	0	0	0	0	0	0	0	0	0	0	0	0	0	0	0	0	0	0	0	0	0	0	0	2
2	0	0	0	0	0	0	0	0	0	0	0	0	0	0	0	0	0	0	0	0	0	0	0	0
0	0	0	0	0	0	0	0	0	0	0	0	0	3	0	0	0	0	0	0	0	0	0	0	0
0	0	0	0	0	0	0	0	0	0	0	0	0	0	0	0	0	0	0	0	0	0	0	0	0
0	0	0	0	0	0	0	0	0	0	0	0	0	0	0	0	0	0	0	0	0	0	0	0	0
0	0	0	0	0	0	0	0	0	0	0	0	0	0	0	0	0	0	0	0	0	0	0	0	0
0	0	0	0	1	0	0	0	0	0	0	0	0	0	0	0	0	0	0	0	0	0	0	0	0
0	0	0	0	0	0	0	0	0	0	0	0	0	0	0	0	0	0	0	0	0	0	0	0	0
0	0	0	0	0	0	0	0	7	0	0	0	0	0	0	0	0	0	0	0	0	0	0	0	0
0	0	0	0	0	0	0	3	0	0	0	0	0	0	0	0	0	0	0	0	0	0	0	0	0
0	0	0	0	0	0	0	0	0	1	0	0	0	0	0	0	0	0	0	0	0	0	0	0	0
0	0	0	0	0	0	0	18	0	0	0	7	0	0	0	0	0	0	0	0	0	0	0	0	0
0	0	0	0	0	0	0	0	0	0	0	0	0	0	0	0	0	0	0	0	0	0	0	0	0
0	0	0	0	8	0	0	25	0	0	0	0	0	0	0	0	0	0	0	0	0	0	0	0	0
0	0	0	0	0	0	0	0	0	0	0	0	0	0	0	0	0	0	0	0	0	0	0	0	0
0	0	0	0	0	0	0	0	0	0	0	0	0	0	0	0	0	0	0	0	0	0	0	0	0
0	0	0	0	0	0	0	0	0	0	0	0	0	0	0	0	0	0	0	0	0	0	0	0	0
0	0	0	0	0	0	0	0	0	0	0	0	0	0	0	0	0	0	0	0	0	0	0	0	0
0	0	0	0	0	0	0	0	3	0	0	0	2	0	0	0	0	0	0	0	15	0	0	0	0
0	0	0	0	0	0	0	0	0	0	0	0	0	0	0	0	0	0	0	0	0	0	0	0	0
0	0	0	0	0	0	0	0	0	0	0	0	0	0	0	0	0	0	0	0	0	0	0	0	0
0	0	0	0	0	0	0	0	0	0	0	0	0	2	0	0	0	0	0	0	0	0	0	0	0

0	0	0	0	0	0	0	0	0	0	0	0	0	0	0	0	0	0	0	0	0	0	0	0	8
0	0	0	0	0	0	0	0	6	21	0	0	0	0	0	0	0	0	0	0	0	0	0	0	0
0	0	0	0	0	0	0	0	0	0	0	0	0	0	0	0	0	0	0	0	0	0	0	0	0
0	89	157	93	0	72	84	342	381	426	15	1	14	2	5	0	0	3	37	17	15	2	1	0	65
0	7	97	0	0	0	11	0	1	94	0	0	0	0	2	0	0	10	0	0	0	0	0	10	0
0	50	19	68	0	77	0	21	88	44	1	0	0	0	1	0	0	0	8	0	0	5	2	0	38
0	0	0	0	0	22	0	0	6	0	0	0	0	0	0	0	0	0	0	0	0	0	0	0	0
0	0	0	0	0	0	7	0	0	0	0	0	0	0	0	0	0	0	0	0	0	0	0	0	0
0	0	0	0	1	0	0	0	0	0	0	0	0	0	0	0	0	0	0	0	0	0	0	0	0
0	0	0	0	0	0	0	0	0	0	0	0	0	0	2	0	0	0	0	0	0	0	0	0	0
0	0	0	0	0	0	0	0	0	0	0	0	0	0	0	0	0	0	0	0	0	0	0	0	0
0	0	0	0	0	0	2	0	0	0	0	0	0	0	0	0	0	0	0	0	0	0	0	0	0
0	0	0	0	0	0	0	0	0	0	0	0	0	0	0	0	0	0	0	0	0	0	0	0	0
0	0	0	0	0	0	0	0	0	0	0	0	0	0	1	0	0	0	0	0	0	0	0	0	0
0	0	0	0	0	0	0	0	0	0	0	0	0	0	0	0	0	0	0	0	0	0	0	0	0
0	0	0	0	0	0	0	0	0	0	0	0	0	0	0	0	0	0	0	0	0	0	0	0	0
0	0	0	0	0	0	0	0	4	0	0	0	0	0	0	0	0	0	0	0	0	0	0	0	0
0	0	0	0	0	0	0	0	0	0	0	0	0	0	0	0	0	0	0	0	0	0	0	0	0
0	0	17	0	0	0	0	0	0	0	0	0	0	0	0	0	0	0	0	0	0	0	0	0	0
0	7	0	0	0	0	0	0	0	0	0	0	0	0	0	0	0	0	0	0	0	0	0	0	0
0	0	0	0	0	0	0	0	0	0	0	0	0	0	0	0	0	0	0	0	0	0	0	0	0
0	0	0	0	0	0	0	0	0	0	0	0	0	0	0	0	0	0	0	0	0	0	0	0	0
17	20	4	258	159	70	138	219	39	2	0	5	0	0	2	0	0	4	28	4	0	0	1	911	0
1	6	0	39	0	0	0	0	0	6	0	4	6	29	0	0	0	1	32	1	0	0	0	0	0
0	30	0	0	0	0	0	62	0	0	0	0	0	0	0	0	0	0	0	0	0	0	0	0	0
0	2	0	0	0	0	0	28	35	0	1	1	0	0	4	1	0	0	0	0	0	0	0	0	0
0	0	0	0	0	0	10	0	0	0	0	0	0	0	0	0	0	0	0	0	0	0	0	0	0
0	49	21	40	0	38	2	0	9	1553	0	0	0	0	0	0	0	31	0	0	0	0	0	0	0
0	12	0	0	0	0	31	0	0	44	0	0	0	0	3	1	0	2	0	0	0	0	0	0	0
0	0	0	0	0	0	0	0	0	0	0	0	0	0	0	0	0	0	0	0	0	0	0	0	0
0	15	0	20	0	33	23	96	4	19	0	0	1	0	0	0	0	13	1	1	6	0	1	1	2
0	0	0	0	0	0	0	0	0	0	0	0	0	0	0	0	0	0	0	0	0	0	0	0	0
0	12	0	9	0	0	23	345	1229	129	0	6	0	8	0	1	0	0	0	0	0	3	0	0	0
0	46	60	34	0	101	15	88	97	357	2	37	11	8	0	3	0	6	0	9	4	1	4	0	15
0	0	0	0	0	0	0	0	0	0	0	0	0	0	0	0	0	0	0	0	0	0	0	0	0
0	0	0	20	0	0	0	0	0	0	0	0	0	0	0	0	0	0	0	0	0	0	0	0	0
0	0	0	0	0	15	0	0	0	0	0	0	0	0	0	0	0	0	0	0	0	0	0	0	0
0	0	0	0	0	0	0	0	0	0	0	0	0	0	0	0	0	0	0	0	0	0	0	0	0
0	4	0	0	0	0	0	15	6	0	0	0	0	0	0	0	0	0	0	5	0	0	0	0	0
0	0	0	9	0	0	0	0	0	0	0	0	0	0	0	0	0	0	0	0	0	0	0	0	0
0	0	0	0	0	0	0	0	0	0	0	0	0	0	0	0	0	0	0	0	0	0	0	0	0
0	1	28	65	0	0	0	117	364	19	0	39	0	12	0	0	2	0	0	5	0	0	0	0	13
0	0	0	0	0	0	2	0	0	0	0	0	0	0	0	0	0	0	0	0	0	0	0	0	0
0	1	0	0	0	0	1	0	0	0	0	0	0	0	0	0	0	0	0	0	0	0	0	0	0
0	0	0	0	0	0	5	0	0	0	0	0	0	0	0	0	0	0	0	0	0	0	0	0	0
0	0	0	2	0	0	0	2	3	0	0	2	0	1	0	0	0	0	0	0	0	0	0	0	1
0	0	9	0	0	0	0	1	44	0	0	2	0	0	0	0	0	0	0	0	0	0	0	0	0
0	0	0	0	0	0	0	0	5	0	0	0	0	0	0	0	0	0	0	0	0	0	0	0	0
0	2	0	0	0	0	0	0	0	0	0	0	0	0	0	0	0	0	0	0	0	0	0	0	0
0	0	0	0	0	0	0	0	0	0	0	0	0	0	0	0	0	0	0	0	0	0	0	0	0
0	0	0	0	0	0	0	0	1	0	0	0	0	0	0	0	0	0	0	0	0	0	0	0	0
0	0	0	0	0	0	0	0	0	0	0	0	0	0	0	0	0	0	0	0	0	0	0	0	0
0	0	0	0	0	0	0	0	4	0	0	0	0	0	0	0	0	0	0	0	0	0	0	0	0
0	0	0	0	0	0	0	29	323	0	0	0	0	0	0	0	0	0	0	0	0	0	0	0	0
0	0	0	0	0	0	0	0	0	0	0	0	0	0	0	0	0	0	0	0	0	0	0	0	0
0	0	0	0	0	0	0	0	0	0	0	0	0	0	0	0	0	0	0	0	0	0	0	0	0
0	0	0	0	0	0	0	0	0	0	0	0	0	0	0	0	0	0	0	0	0	0	0	0	0
0	0	0	0	0	0	0	1	4	0	0	0	0	0	0	0	0	0	0	0	0	0	0	0	0
0	0	0	0	0	0	0	0	9	0	0	0	0	0	0	0	0	0	0	0	0	0	0	0	0
0	0	0	0	0	0	0	0	0	0	0	0	0	0	0	0	0	0	0	0	0	0	0	0	0
0	0	0	0	0	0	0	0	3	0	0	0	0	0	0	0	0	0	0	0	0	0	0	0	0
0	0	0	0	0	0	0	0	1	0	0	0	0	0	0	0	0	0	0	0	0	0	0	0	0
0	0	0	5	0	0	0	0	0	0	0	0	0	0	0	0	0	0	0	0	0	0	0	0	0
0	0	0	0	0	0	0	0	0	0	0	0	0	0	0	0	0	0	0	0	0	0	0	0	0
0	0	0	0	0	0	0	0	0	0	0	0	0	0	0	0	0	0	0	0	0	0	0	0	0
0	0	0	0	0	0	0	0	3	0	0	0	0	0	0	0	0	0	0	0	0	0	0	0	0
0	0	0	0	0	0	0	0	0	0	0	0	0	0	0	0	0	0	0	0	0	0	0	0	0
0	0	0	0	0	0	1	0	0	0	0	0	0	0	0	0	0	0	0	0	0	0	0	0	0
0	0	0	0	0	0	0	0	1	0	0	0	0	0	0	0	0	0	0	0	0	0	0	0	0
0	0	0	0	0	0	0	0	1	0	0	0	0	0	0	0	0	0	0	0	0	0	0	0	0
0	0	0	0	0	0	0	0	5	1	0	1	0	0	0	0	0	0	0	0	0	0	0	0	0
0	0	0	0	0	0	16	6	33	73	0	0	0	0	0	0	0	0	4	0	0	0	0	0	0
0	0	0	0	0	0	0	0	0	0	0	0	0	0	0	0	0	0	0	0	0	0	0	0	0
0	0	0	0	0	0	7	0	0	0	0	0	0	0	0	0	0	0	0	0	0	0	0	0	0
0	0	0	0	0	0	0	0	0	0	0	0	0	0	0	0	0	0	0	0	0	0	0	0	0
0	0	0	0	0	0	0	0	0	0	0	0	0	0	0	0	0	0	0	0	0	0	0	0	0
0	0	0	0	0	0	12	0	0	0	0	0	0	0	0	0	0	0	0	0	0	0	0	0	0
0	7	1	20	0	0	24	111	29	21	0	0	0	0	0	0	0	0	16	5	0	0	1	0	0
0	1	0	1	0	0	0	0	1	0	0	0	0	0	0	0	0	0	0	0	0	0	0	0	0
0	0	0	0	0	0	0	0	23	0	1	0	0	0	0	0	0	0	0	0	0	0	0	0	0
0	0	0	9	0	0	0	1	2	0	0	3	0	0	0	0	0	0	0	0	0	0	0	0	0
0	0	0	0	0	0	0	0	0	0	1	0	0	0	0	0	0	0	0	0	0	0	0	0	0
0	0	0	0	0	2	0	0	1	0	0	0	0	0	0	0	0	0	0	0	0	0	0	0	0
0	1	0	0	4	0	0	0	0	0	0	0	0	0	0	0	0	0	0	0	0	0	0	0	0
0	0	0	0	0	0	7	1	18	0	0	0	0	0	0	0	0	0	0	0	0	0	0	0	0
0	0	0	3	0	6	9	7	8	5	0	0	0	0	0	0	0	0	14	0	0	0	0	0	0
0	0	0	0	0	0	0	12	0	0	0	0	0	0	0	0	0	0	0	0	0	0	0	0	0
0	0	0	0	0	0	0	0	0	0	0	0	0	0	0	0	0	0	0	0	0	0	0	0	0
0	0	0	0	0	0	0	0	0	0	0	0	0	0	0	0	0	0	0	0	0	0	0	0	0
0	0	0	0	0	0	0	1	4	0	0	0	0	0	0	0	0	0	0	0	0	0	0	0	0
0	0	0	0	0	0	1	0	33	0	0	0	0	0	0	0	0	0	0	0	0	0	0	0	0
0	0	0	0	0	0	0	0	0	0	0	0	0	0	0	0	0	0	0	0	0	0	0	0	0
0	0	0	0	0	0	0	0	13	21	0	0	0	0	0	0	0	0	0	0	0	0	0	0	0
0	0	0	0	0	0	0	0	1	0	0	0	0	0	0	0	0	0	0	0	0	0	0	0	0
0	0	0	0	0	0	0	0	3	0	0	0	0	0	0	0	0	0	0	0	0	0	0	0	0
0	0	0	0	0	0	0	0	0	0	0	0	0	0	0	0	0	0	0	0	0	0	0	0	0
0	0	0	9	0	0	0	0	0	0	0	0	0	0	0	0	0	0	0	0	0	0	0	0	0
0	0	0	0	0	0	0	8	0	0	0	0	0	0	0	0	0	0	0	0	0	0	0	0	0
0	0	0	0	0	0	0	20	7	0	0	0	0	0	0	0	0	0	0	0	0	0	0	0	0
0	0	0	0	0	0	0	0	0	0	0	0	0	0	0	0	0	0	0	0	0	0	0	0	0
0	0	0	0	0	0	0	0	0	0	0	0	0	0	0	0	0	0	0	0	0	0	0	0	0
0	0	0	0	0	0	0	0	0	0	0	0	0	0	0	0	0	0	0	0	0	0	0	0	0
0	0	0	0	0	0	0	0	0	0	0	0	0	0	0	0	0	0	0	0	0	0	0	0	0
0	0	0	0	0	0	0	0	0	0	0	1	0	0	0	0	0	0	0	0	0	0	0	0	0
0	0	0	0	0	0	0	0	0	0	0	0	0	0	0	0	0	0	0	0	0	0	0	0	0
0	0	0	0	0	0	0	0	0	0	0	0	0	0	0	0	0	0	0	0	0	0	0	0	0
0	0	0	0	0	0	0	5	0	0	0	0	0	0	0	0	0	0	0	0	0	0	0	0	0
0	0	0	0	0	0	0	0	0	0	0	0	0	0	0	0	0	0	0	0	0	0	0	0	0
0	0	0	0	0	0	0	0	0	0	0	0	0	0	0	0	0	0	0	0	0	0	0	0	0
0	0	0	0	0	0	0	0	0	0	0	0	0	0	0	0	0	0	0	0	0	0	0	0	0
0	0	0	0	0	0	0	0	0	0	0	0	0	0	0	0	0	0	0	0	0	0	0	0	0
0	0	0	0	0	0	0	0	0	0	0	0	0	0	0	0	0	0	0	0	0	0	0	0	0
0	0	0	0	0	0	0	0	0	0	0	0	0	0	0	0	0	0	0	0	0	0	0	0	0
0	0	0	0	0	0	0	0	0	0	0	0	0	0	0	0	0	0	0	0	0	0	0	0	0
0	0	0	0	0	0	0	0	0	0	0	0	0	0	0	0	0	0	0	0	0	0	0	0	0
0	0	0	0	0	0	0	0	0	0	0	0	0	0	0	0	0	0	0	0	0	0	0	0	0
0	0	0	0	0	0	0	0	0	0	0	0	0	0	0	0	0	0	0	0	0	0	0	0	0
0	0	0	0	0	0	0	0	0	0	0	0	0	0	0	0	0	0	0	0	0	0	0	0	0
0	0	0	0	0	0	0	0	0	0	0	0	0	0	0	0	0	0	0	0	0	0	0	0	0
0	0	0	0	0	0	0	0	0	0	0	0	0	0	0	0	0	0	0	0	0	0	0	0	0
0	0	0	0	0	0	0	0	0	0	0	0	0	0	0	0	0	0	0	0	0	0	0	0	3
0	0	0	0	0	0	0	0	0	0	0	11	0	0	0	0	0	0	0	0	0	0	0	0	0
0	0	0	0	0	0	0	0	0	0	0	0	0	0	0	0	0	0	0	0	0	0	0	0	0
0	0	0	0	0	0	0	0	0	0	0	0	0	0	0	0	0	0	3	0	2	0	0	0	0
0	0	0	0	0	0	7	0	43	0	0	0	0	0	0	0	0	0	0	0	0	0	0	0	0
0	0	274	19	0	0	0	50	158	35	0	13	0	0	0	0	0	0	0	5	0	0	0	0	0
0	0	0	0	0	0	0	0	0	0	0	0	0	0	0	0	0	0	0	0	0	0	0	0	0
0	0	0	0	0	0	0	0	0	0	0	0	0	0	0	0	0	0	0	0	0	0	0	0	0
0	8	0	0	0	0	0	0	0	0	0	0	0	0	0	0	1	0	0	0	0	0	0	0	0
0	0	0	0	0	0	0	0	0	0	0	0	0	0	0	0	0	0	0	0	0	3	0	0	0
0	0	0	0	0	0	0	0	0	0	0	0	0	0	0	0	0	0	0	0	0	0	0	0	0
0	0	0	0	0	0	0	0	0	0	0	0	0	0	0	0	0	0	0	0	0	0	0	0	0
0	0	0	0	0	0	0	0	0	0	0	0	0	0	0	0	0	0	0	0	0	0	0	0	0
0	0	0	0	0	0	0	0	0	0	0	0	0	0	0	0	0	0	0	0	0	0	0	0	0
0	1	0	0	0	0	0	0	0	2	0	0	0	0	0	0	0	0	0	0	0	0	0	0	0
0	0	0	0	0	0	0	0	0	0	0	0	0	0	0	0	0	0	1	0	0	0	0	0	1
0	0	0	0	0	0	0	0	0	0	0	0	0	0	0	0	0	0	0	0	0	0	0	0	0
0	21	0	0	9	0	3	11	23	0	0	0	2	0	0	0	0	1	1	0	0	0	0	0	0
0	0	0	0	0	0	0	0	0	0	0	0	0	0	0	0	0	0	0	0	0	0	0	0	0
0	0	0	0	0	6	0	0	1	0	0	0	0	0	0	0	0	0	0	0	0	0	0	0	0
0	0	0	0	0	0	0	0	5	0	0	0	0	0	0	0	0	0	0	0	0	0	0	0	0
0	0	0	0	0	0	0	0	0	0	0	0	0	0	0	0	0	0	0	0	0	0	0	0	0
0	0	0	0	0	0	0	5	1	0	0	0	0	0	0	0	1	0	0	0	0	0	0	0	0
0	0	0	0	0	0	0	0	0	0	0	0	0	0	0	0	0	0	0	0	0	0	0	0	0
0	0	0	0	0	0	0	0	0	0	0	0	0	0	0	0	0	0	0	0	0	0	0	0	0
0	0	0	0	0	0	0	0	0	0	0	0	0	0	0	0	0	0	0	0	0	0	0	0	0
0	0	0	0	0	0	0	0	0	0	0	0	0	0	0	0	0	0	0	0	0	0	0	0	0
0	0	0	0	0	0	0	0	0	0	0	0	0	0	0	0	0	0	0	0	0	0	0	0	0
0	0	0	0	0	0	0	0	0	0	0	0	0	0	0	0	0	0	0	0	0	0	0	0	0
0	0	0	0	0	0	0	0	0	0	0	0	0	0	0	0	0	0	0	0	0	0	0	0	0
0	1	0	0	0	0	2	0	0	0	0	0	0	0	0	0	0	0	0	0	0	0	0	0	0
4	0	0	0	0	0	0	0	9	0	0	0	0	0	0	0	0	0	0	0	0	0	0	0	0
0	0	0	0	0	0	0	0	1	0	0	0	0	0	0	0	0	0	0	0	0	0	0	0	0
0	4	0	0	2	0	0	28	29	0	5	0	0	0	1	0	1	0	15	0	0	1	0	0	0
0	0	0	0	0	0	0	0	0	0	0	9	0	0	0	0	0	0	0	0	0	0	0	0	0
0	0	0	1	5	21	35	95	47	0	0	3	0	9	0	0	0	0	3	12	1	0	0	0	0
0	0	0	0	0	0	0	0	1	17	0	0	0	0	0	0	0	0	0	0	0	0	0	0	1
0	22	138	0	148	1	86	43	19	227	0	0	0	1	1	0	1	1	0	0	11	0	0	0	5
0	0	277	56	0	0	9	96	41	14	0	0	0	29	0	0	0	0	0	0	0	1	0	5	0
0	0	0	0	0	0	0	0	0	0	0	0	0	0	0	0	0	0	0	0	0	0	0	0	0
0	7	0	0	0	0	0	0	12	36	0	0	0	0	0	0	0	0	0	0	0	0	0	0	0
0	0	0	0	0	0	0	0	8	0	0	0	0	0	0	0	0	0	0	0	0	0	0	0	0
0	0	0	0	0	0	0	0	0	0	0	0	0	0	0	0	0	0	0	0	0	0	0	0	0
0	0	21	0	0	0	0	0	0	0	0	0	0	0	0	0	0	0	0	0	0	0	0	0	0
0	0	0	0	0	0	0	0	0	0	0	0	0	0	0	0	0	0	0	0	0	0	0	0	0
1	13	16	11	1	12	35	86	68	40	1	2	1	142	7	0	0	0	0	0	1	0	1	3	6
0	0	41	0	0	0	0	37	5	0	0	0	0	0	0	0	0	0	0	47	0	0	0	0	0
0	0	0	0	0	0	7	0	0	0	0	0	0	0	0	0	0	0	0	0	0	0	0	0	0
1	209	56	40	5	36	73	80	78	406	0	10	0	8	0	1	0	2	0	16	1	0	0	217	6
0	3	34	0	0	15	0	43	32	0	0	0	0	0	0	0	2	0	0	0	0	0	0	0	0
2	6	78	0	2	3	0	36	161	0	0	0	0	0	0	0	0	0	0	0	0	0	0	0	0
19	0	0	0	37	0	0	0	0	0	0	0	0	0	0	0	0	0	0	0	0	0	0	21	0
0	0	0	0	2	0	0	0	0	0	0	0	0	0	0	0	0	0	0	0	0	0	0	0	0
0	0	0	0	0	0	0	0	0	0	0	0	0	5	0	0	0	1	0	0	0	0	0	0	0
0	0	0	0	0	0	0	30	93	1070	0	0	0	0	0	0	0	0	0	0	0	0	0	0	0
0	1	165	7	2	55	0	3	95	0	0	0	17	0	0	0	0	0	0	0	0	0	0	0	0
0	0	0	0	8	3	0	17	11	5	0	0	0	0	0	0	0	0	0	0	0	0	0	0	0
0	0	0	0	0	0	0	0	0	0	0	0	0	0	0	0	0	0	0	0	0	0	0	0	0
0	0	0	0	0	0	0	0	0	0	0	0	0	0	0	0	0	0	0	0	0	0	0	0	0
0	0	0	2	2	0	6	0	0	2	0	0	0	0	0	0	0	0	0	0	0	1	0	15	0
0	0	0	0	0	0	0	0	0	0	0	0	0	0	0	0	0	0	0	0	0	0	0	0	0
0	0	0	0	0	0	1	0	0	0	0	0	0	0	0	0	0	0	0	0	0	0	0	0	0
0	0	0	0	1	0	0	0	2	0	0	0	0	0	0	0	0	0	0	0	0	0	0	0	0
0	0	0	0	8	0	0	0	3	0	0	0	8	0	0	0	0	0	0	0	0	0	0	0	0
0	0	0	0	0	0	0	0	0	0	0	0	0	0	0	0	2	0	0	1	0	0	0	0	0
0	0	0	0	0	0	0	0	0	0	0	0	0	0	0	0	0	0	0	0	0	0	0	0	0
0	0	0	0	0	0	0	0	0	0	0	0	0	0	0	0	0	0	0	0	0	0	0	0	0
0	0	0	0	0	8	0	9	2	0	0	3	0	0	0	0	0	0	0	0	0	0	0	0	0
0	0	0	0	0	0	0	0	0	0	0	0	0	0	0	0	0	0	0	0	0	0	0	0	0
0	0	0	0	0	0	0	0	3	0	0	0	0	0	0	0	0	0	0	0	0	0	0	0	0
0	0	0	0	0	0	0	3	0	0	0	0	0	0	0	0	0	0	0	0	0	0	0	0	0
0	0	0	0	0	0	0	0	0	33	0	0	0	0	0	0	0	0	0	0	0	0	0	0	0
0	0	1	0	0	0	0	0	0	0	0	0	0	0	0	0	0	0	0	0	0	0	0	0	0
0	0	0	0	0	1	0	3	0	0	0	0	0	0	0	0	1	0	0	0	0	0	0	0	0
0	0	0	0	0	0	0	0	1	0	0	0	0	0	0	0	0	0	0	0	0	0	0	0	0
0	0	0	19	0	0	0	0	5	0	0	0	0	1	0	0	0	0	0	0	0	0	0	0	0
0	0	0	0	0	0	0	0	2	0	0	0	0	0	0	0	0	0	0	0	0	0	0	0	0
0	0	0	0	0	0	0	0	0	0	0	0	0	0	0	0	0	0	0	0	0	0	0	0	0
0	0	0	0	0	0	0	0	0	0	0	0	0	0	0	0	0	0	0	0	0	0	0	0	0
0	0	0	0	0	0	0	0	0	0	0	0	0	0	0	0	0	0	0	0	0	0	0	0	0
0	0	0	0	0	3	0	0	0	0	0	0	0	0	0	0	0	0	0	0	0	0	0	0	0
0	0	20	20	0	133	26	107	111	4	0	0	2	0	0	0	3	0	0	1	0	0	0	39	9
0	0	0	0	2	0	0	34	0	0	0	0	0	0	0	0	0	0	0	0	0	0	0	0	0
0	0	0	1	0	2	0	0	1	0	0	0	0	0	0	0	0	0	0	0	0	0	0	0	1
0	0	0	0	2	13	2	37	5	52	0	7	0	0	0	0	1	0	0	0	0	0	0	0	12
0	0	0	0	0	0	1	0	0	0	0	2	3	0	0	0	0	0	0	0	0	0	0	0	0
0	0	0	0	0	0	0	0	4	0	0	0	0	0	0	0	0	0	0	0	0	0	0	0	0
0	0	0	0	0	0	0	0	0	0	0	0	0	0	0	0	0	0	0	0	0	0	0	0	0
0	2	0	0	0	0	0	27	17	0	0	0	0	0	0	0	0	0	0	0	0	0	0	0	0
0	0	0	0	0	0	0	0	0	0	0	0	0	0	0	0	0	0	0	0	0	0	0	0	0
0	0	0	0	0	0	0	0	0	0	0	0	0	0	0	0	0	0	0	0	0	0	0	0	0
0	0	0	0	0	0	0	0	0	0	0	0	0	0	0	0	0	0	0	0	0	0	0	0	0
0	0	0	0	0	5	2	6	2	0	0	0	0	0	0	0	0	0	0	0	0	0	0	0	0
0	1	0	0	0	0	0	0	6	0	0	0	0	0	0	0	0	0	0	0	0	0	0	2	0
0	0	0	0	0	0	0	0	0	0	0	0	0	0	0	0	0	0	0	0	0	0	0	0	0
0	0	0	0	0	0	0	0	3	0	0	0	0	0	0	0	0	0	0	0	0	0	0	0	0
0	0	0	0	0	0	0	0	1	0	0	0	0	0	0	0	0	0	0	0	0	0	0	0	0
0	0	0	0	0	0	0	0	0	0	0	0	0	0	0	0	0	0	0	0	0	0	0	0	0
0	13	5	4	8	0	9	77	19	0	0	5	12	0	3	0	3	1	3	2	0	0	0	0	0
0	4	0	1	50	40	18	133	239	0	0	1	0	2	0	1	0	0	0	0	0	0	0	0	0
0	0	0	0	1	0	0	0	2	0	0	0	0	12	0	0	1	0	0	0	0	0	0	0	0
0	0	0	0	0	0	0	0	0	0	0	0	0	0	0	0	0	0	0	0	0	0	0	0	0
0	0	0	0	0	0	0	0	0	0	0	0	0	0	0	0	0	0	0	0	0	0	0	0	0
0	0	0	2	0	0	0	0	0	0	0	0	0	0	0	0	0	0	0	0	0	0	0	0	0
0	0	0	0	13	0	12	0	9	0	0	1	0	0	0	0	0	0	0	0	0	0	0	0	0
0	0	0	0	0	0	0	1	0	0	0	0	0	0	0	0	0	0	0	0	0	0	0	0	0
0	0	0	0	0	0	0	0	45	0	0	0	0	0	0	1	0	0	0	0	0	0	4	0	0
0	0	0	17	0	0	16	0	2	0	0	0	0	0	0	0	0	0	0	0	0	0	0	0	0
0	0	0	0	0	0	0	0	0	0	0	0	0	0	0	0	0	0	0	0	0	0	0	0	0
0	0	0	0	0	0	0	0	0	0	0	0	0	0	0	0	0	0	0	0	0	0	0	0	0
0	0	0	0	0	0	0	0	0	0	0	0	0	0	0	0	0	0	0	0	0	0	0	0	0
0	0	0	0	0	0	0	0	0	0	0	0	0	0	0	0	0	0	0	0	0	0	0	0	0
0	0	0	0	0	0	0	0	0	0	0	0	0	0	0	0	0	0	0	0	0	0	0	0	0
0	0	0	0	0	0	0	0	0	0	0	0	0	0	0	0	0	0	0	0	0	0	0	0	0
0	0	0	0	0	0	0	36	0	0	0	0	0	0	0	0	0	0	0	0	0	0	0	0	0
0	0	0	0	0	0	0	0	0	0	0	0	0	0	0	0	0	0	0	0	0	0	0	0	0
0	0	0	0	0	0	0	0	0	0	0	0	0	0	0	0	0	0	0	0	0	0	0	0	0
0	0	0	0	0	0	0	0	0	0	0	0	0	0	0	0	0	0	0	0	0	0	0	0	0
0	0	0	0	0	0	0	0	0	0	0	0	0	0	0	0	0	0	0	0	0	0	0	0	0
0	0	0	0	0	0	0	0	0	0	0	0	0	0	0	0	0	0	0	0	0	0	0	0	0
0	0	0	0	0	0	0	0	0	0	0	0	0	0	0	0	0	0	0	0	0	0	0	0	0
0	0	0	0	0	0	0	0	1	0	0	0	0	0	0	0	0	0	0	0	0	0	0	0	0
0	0	0	0	0	0	0	0	0	0	0	0	0	0	0	0	0	0	0	0	0	0	0	0	0
0	0	0	0	0	0	0	0	0	0	0	0	0	0	0	0	0	0	0	0	0	0	0	0	0
0	0	0	0	0	0	0	0	0	0	0	0	0	0	0	0	0	0	0	0	0	0	0	0	0
0	0	0	0	0	0	0	0	3	0	0	0	0	0	1	0	0	0	0	0	0	0	0	0	0
0	0	0	0	0	0	3	0	0	0	0	0	0	0	0	0	0	0	0	0	0	0	0	0	0
0	0	0	0	0	0	0	0	0	0	0	0	0	0	0	0	0	0	0	0	0	0	0	0	0
0	0	0	0	0	0	0	0	0	0	0	0	0	0	0	0	0	0	0	0	0	0	0	0	0
0	0	0	0	0	0	0	0	0	0	0	0	1	0	0	1	0	0	0	0	1	0	0	0	0
0	0	0	0	0	0	0	0	0	0	0	0	0	0	0	0	0	0	0	0	0	0	0	0	0
1280	5057	4877	5587	10615	4450	3927	7049	12516	5205	2854	7484	4983	5677	5659	582	6432	1609	6784	5244	3274	1180	5278	3774	5454
